# Click and Cut: a click chemistry approach to developing oxidative DNA damaging agents

**DOI:** 10.1093/nar/gkab817

**Published:** 2021-09-27

**Authors:** Natasha McStay, Creina Slator, Vandana Singh, Alex Gibney, Fredrik Westerlund, Andrew Kellett

**Affiliations:** School of Chemical Sciences and National Institute for Cellular Biotechnology, Dublin City University, Glasnevin, Dublin 9, Ireland; Synthesis and Solid-State Pharmaceutical Centre, School of Chemical Sciences, Dublin City University, Glasnevin, Dublin 9, Ireland; School of Chemical Sciences and National Institute for Cellular Biotechnology, Dublin City University, Glasnevin, Dublin 9, Ireland; Department of Biology and Biological Engineering, Chalmers University of Technology, Gothenburg, Sweden; School of Chemical Sciences and National Institute for Cellular Biotechnology, Dublin City University, Glasnevin, Dublin 9, Ireland; Synthesis and Solid-State Pharmaceutical Centre, School of Chemical Sciences, Dublin City University, Glasnevin, Dublin 9, Ireland; Department of Biology and Biological Engineering, Chalmers University of Technology, Gothenburg, Sweden; School of Chemical Sciences and National Institute for Cellular Biotechnology, Dublin City University, Glasnevin, Dublin 9, Ireland; Synthesis and Solid-State Pharmaceutical Centre, School of Chemical Sciences, Dublin City University, Glasnevin, Dublin 9, Ireland

## Abstract

Metallodrugs provide important first-line treatment against various forms of human cancer. To overcome chemotherapeutic resistance and widen treatment possibilities, new agents with improved or alternative modes of action are highly sought after. Here, we present a click chemistry strategy for developing DNA damaging metallodrugs. The approach involves the development of a series of polyamine ligands where three primary, secondary or tertiary alkyne-amines were selected and ‘clicked’ using the copper-catalysed azide-alkyne cycloaddition reaction to a 1,3,5-azide mesitylene core to produce a family of compounds we call the ‘Tri-Click’ (TC) series. From the isolated library, one dominant ligand (TC1) emerged as a high-affinity copper(II) binding agent with potent DNA recognition and damaging properties. Using a range of *in vitro* biophysical and molecular techniques—including free radical scavengers, spin trapping antioxidants and base excision repair (BER) enzymes—the oxidative DNA damaging mechanism of copper-bound TC1 was elucidated. This activity was then compared to intracellular results obtained from peripheral blood mononuclear cells exposed to Cu(II)–TC1 where use of BER enzymes and fluorescently modified dNTPs enabled the characterisation and quantification of genomic DNA lesions produced by the complex. The approach can serve as a new avenue for the design of DNA damaging agents with unique activity profiles.

## INTRODUCTION

Nucleic acids are ubiquitous biomolecules chiefly responsible for mediating faithful cellular replication and translation in nearly all forms of cellular life. Both DNA and RNA are therefore subjected to intensive drug discovery efforts so that cancer, monogenetic, and pathogenic diseases can be effectively treated (1). Since double helical DNA encodes genetic information, it serves as a primary target for small molecule binding agents (2). These interactions typically involve molecular recognition such as the curvature of the major or minor groove, the negatively charged phosphate backbone, or the space between specific base pairs (steps) required for intercalation. Binding at the molecular level broadly falls into two main categories: covalent and non-covalent. Covalent binders directly coordinate nucleic acids distorting their shape, structure and function and are often used as first line treatments against cancer; the interaction is considered permanent as it can be removed only by DNA excision repair enzymes. Non-covalent binders interact transiently but in doing so also distort shape and function as the primary structure reorganises to accommodate the ligand (3,4). Characterising purely covalent or non-covalent interactions can be difficult as many covalent binders must firstly interact with DNA preassociativity using non-covalent interactions (kinetically fast) prior to bond formation (kinetically slow). A specific example of this effect can be traced in the interaction of the anticancer drug cisplatin (*cis*-[Pt(NH_3_)_2_Cl_2_]) where non-covalent am(m)ine-phosphate interactions provide initial anchoring of the molecule prior to Pt(II) crosslinking adjacent purine bases (5). Conversely, several important classes of DNA damaging drugs including metallobleomycin and neocarzinostatin are potent non-covalent binding agents. But in their subsequent oxidative DNA damaging phases they activate C–H deoxyribose bonds producing double stand breaks that covalently modify the underlying nucleic acid structure (6).

Several clues for developing new bioactive agents have been taken from naturally occurring molecules that recognise DNA. For instance, antibiotic groove binders, such as netropsin and distamycin A contain heterocyclic pyrrole rings that contort into a crescent shape that mimics the curvature of the A–T rich minor groove (7–9). Alternatively, heterocycles of extended planarity can π-stack between adjacent base pairs. The naturally occurring glycopeptide bleomycin contains one such moiety—a bithiazole group which intercalates duplex DNA via the minor groove and which is proximate to a polyamine chelator that coordinates first row transition metals including copper and iron (10). An activated metallobleomycin intermediate (e.g. bleomycin-Fe(III)-OOH) then promotes H-atom abstraction from deoxyribose to form single strand breaks (SSBs), and more difficult to repair double strand breaks (DSBs) central to its anticancer properties (11). Similar to the bithiazole moiety of bleomycin, a thiazole/5-hydroxyoxazole tail was recently proposed as the intercalating unit of macrocyclic colibactin—a human gut bacterial genotoxin which induces DNA double-strand breaks via copper-mediated oxidative cleavage (12) but which may ostensibly alkylate DNA due to the presence of an electrophilic cyclopropane group (13–15).

Copper complexes have recently emerged as attractive chemotypes for treating human cancer. Part of this interest stems from their redox properties, wide structural variability, and the bioavailability of copper. In recent years several promising preclinical Cu(II) complexes, including the naturally occurring polypeptide ATCUN (amino terminal Cu(II) and Ni(II) binding) motif, (16) were applied as DNA damaging pro-oxidants within human cancer cells. Starting with the Cu(II) *bis*-1,10-phenanthroline chemotype (17)—recognised as the first artificial chemical nuclease—it was shown how introducing designer phenazine ligands with extended planarity enhances DNA binding and cleavage activity (18). More recently, tri- and dipodal caging ligands such as *tris*-(2-pyridylmethyl)amine (TMPA) (19) and *di*-(2-pycolylamine) (DPA) (20) were developed to improve solution stability and allow coordination of designer intercalating groups. Several of these caging complexes displayed encouraging activity against recalcitrant human cancers including neuroblastoma and pancreatic ductal adenocarcinoma (21). Despite this promise, some of the most interesting preclinical agents have emerged from polynuclear metal complex design. Polynuclear systems can mediate DNA damage in the absence of exogenous reductant—a property referred to as ‘self-activation’—with selective interactions such as discrimination between AT/AT and TA/TA base pairs (22) along with discrete binding in the major groove, (23) at helix-coil junctions, (24–26) or at three-way junctions (27) are possible through careful design. The use of polynuclear complexes therefore provides cooperative interactions at the drug–DNA interface that are not possible to achieve with simple mononuclear agents.

We recently reported an unusual *C*_3_-symmetric opioid scaffold with nucleic acid condensation properties and investigated its non-viral transfection properties (28). The preparation of this scaffold relied on coupling three morphine, heterocodeine, oripavine or codeine (29) molecules to a central mesitylene core. Protonation of a tertiary amine (present in the piperidine ring of each opioid) then provided an overall 3+ cationic charge central to DNA charge neutralisation and condensation. In this work, we question if a similar *C*_3_-symmetric approach could be applied to develop therapeutically active DNA oxidants, and if click chemistry could provide a modular strategy for the development of such agents. Although click chemistry has an extensive range of applications in the field of nucleic acid click chemistry, (30–33), it has not yet been widely employed to construct DNA binding metallodrugs (34–36). An exception is polyamine G-quadruplex stabilising compounds where multiple triazole rings were found not only to anchor specific, protonatable, amine side chains but also played crucial roles in π-stacking interactions with terminal G-quartets (34). To facilitate the current strategy, we prepared a 1,3,5-azide mesitylene core and applied copper(I)-catalysed azide-alkyne cycloaddition (CuAAC) click chemistry using primary, secondary and tertiary alkyne-amines to produce a library we call the ‘Tri-Click’ (TC) series. TC compounds were then investigated for their ability to coordinate first row transition metals with one ligand (TC1) emerging as a high-affinity copper(II) binding agent with potent DNA recognition and damaging properties.

## MATERIALS AND METHODS

### General remarks

Chemicals and reagents were sourced from Sigma-Aldrich and Tokyo Chemical Industry (TCI) and were used without any further purification. HPLC grade chloroform, methanol, and acetonitrile were used without further purification. All other solvents were used as supplied. Detailed synthetic schemes are shown in Supplementary Section S-1. All novel compounds were characterised by melting point (MP) (when appropriate), nuclear magnetic resonance (NMR) spectroscopy, attenuated total reflectance (ATR) Fourier transform infrared (FTIR) spectroscopy, and electron spray ionisation mass spectrometry (ESI-MS). Thin layer chromatography was performed on Fluka Silica gel (60 F254) coated on aluminium plates and visualised using UV light. Davisil 60 Å silica gel was used for column chromatography. ^1^H and ^13^C NMR spectra were obtained on a Bruker AC 400 MHz and 600 MHz NMR spectrometer (see supplementary section S-4). FT-IR spectra were collected on Perkin Elmer Spectrum Two spectrometer. Electrospray ionisation mass spectra were recorded using a Thermo Fisher Exactive Orbitrap mass spectrometer coupled to an Advion TriVersa Nanomate injection system with samples prepared in 100% HPLC-grade acetonitrile or methanol. Accurate mass spectrometry was conducted on a MaXis HD quadrupole electrospray time-of-flight (ESI-QTOF) mass spectrometer (Bruker Daltonik GmbH, Bremen, Germany), using a glass syringe (Hamilton) and syringe pump (KD Scientific, Model 781100) for infusions at a flow rate of 3 μl/min.

**Caution!** Sodium azide is acutely toxic and is an explosion hazard. Refer to organic azide stability prior to the preparation of any azido compounds. The total number of nitrogen atoms in a final organic azide should not exceed that of carbon. Organic azides with C/N ratio of <1 should never be isolated. It may be synthesized if the azide is a transient intermediate species and the limiting reagent in the reaction mixture and is limited to a maximum quantity of 1 g. Each azido compound should be individually evaluated.

GenElute-Mammalian Genomic DNA Miniprep Kits, tiron, d-mannitol, sodium pyruvate, APTES, ATMS, β-mercaptoethanol, l-histidine, calf-thymus (ctDNA, Ultra-Pure 15633019) were purchased from Invitrogen while, poly[d(A-T)_2_)] (P0883) and poly[d(G-C)_2_] (P9389) were purchased from Sigma-Aldrich. CutSmart buffers, pUC19 plasmid (N3041) and repair enzymes—Fpg (M0240S), Endo III (M0268S), Endo IV (M0304S), Endo V (M0305S), hAAG (M0313S) and APE1 (M0282S)—were purchased from New England Biolabs. Aminoallyl-dUTP-ATTO-647N and YOYO-1 were purchased from Jena Bioscience and Invitrogen, respectively.

The pH was monitored by a Mettler Toledo InLab Expert Pro-ISM pH probe. DNA binding analysis was conducted on a Bio-Tek, Synergy HT fluorescent microplate reader with Gen5 software. UV-visible spectrometry studies were carried out on a Shimadzu UV-2600. AFM images where captured on Bruker Dimension Icon AFM equipped with super sharp silicon cantilevers (SSS-NCHR, Windsor Scientific Ltd). Zeiss Observer.Z1 equipped with an Andor iXON Ultra EMCCD camera was used to obtain fluorescence images.

### Ligand preparation

**2,4,6-*Tris*-(azidomethyl)-mesitylene (triazide)**. Synthesis was carried out as per literature with slight modifications (37). To a solution of 2,4,6-*tris-*(bromomethyl)-mesitylene (1.025 g, 2.56 mmol) in DMF (25 ml), sodium azide (1.00 g, 15.38 mmol) was added in portions over ice over a period of 20 min. (**Caution!** Sodium azide is acutely toxic and is an explosion hazard. Refer to organic azide stability prior to the preparation of any azido compounds). The reaction was stirred on ice for 1 h prior to stirring at rt for 23 h. The reaction was quenched with 8 ml of H_2_O, and extracted with EtOAc (3 × 20 ml). The combined organic phase was washed with H_2_O (5 × 5 ml) and the organic layer dried over MgSO_4_, filtered and solvents removed by rotary evaporation. The sample was recrystallized from a solvent system of hex:EtOAc (5:1) to afford the triazide (0.677 g, 2.37 mmol, 93%) as a white crystalline solid. ^1^H NMR (600 MHz, CDCl_3_) δ: 4.50 (s, 6H), 2.46 (s, 9H).^13^C NMR (151 MHz, CDCl_3_) δ: 138.15, 130.88, 77.27, 77.06, 76.85, 48.94, 16.50. ^1^H and ^13^C NMR were in agreement with literature data (37). IR (ATR, cm^–1^): 2901, 2085, 1678, 1572, 1449, 1232, 1072, 859, 698, 640, 552.

**3-Ethynyl-*N,N*-dimethylaniline (1)**. Product was prepared according to literature procedures with slight modifications (38). To a solution of 3-ethynylaniline (1.034 g, 8.83 mmol) and caesium carbonate (8.306 g, 25.5 mmol) in dry DMF (50 ml) under nitrogen, methyl iodide was added drop wise over 15 min (1.7 ml, 26.0 mmol). The reaction was heated to 40°C for 24 h and was monitored by TLC (Hex:EtOAc). From the complete conversion by TLC, the reaction was cooled and diluted with H_2_O (100 ml) and extracted with EtOAc (3 × 30 ml). The organic layers were combined and washed with H_2_O (3 × 10 ml) and finally with brine solution. The organic layer was dried over MgSO_4_ and reduced to dryness. Product was purified by column chromatography (SiO_2_, Hex:EtOAc, 9:1) as a yellow liquid (383 mg, 2.64 mmol, 30%).^1^H NMR (400 MHz, CDCl_3_) δ: 7.22–7.16 (m, 1H), 6.90–6.84 (m, 2H), 6.74 (ddd, *J* = 8.4, 2.6, 1.0 Hz, 1H), 3.03 (s, 1H), 2.95 (s, 6H). The ^1^H NMR spectrum agreed with literature data (39).

**4-Ethynyl-*N,N*-dimethylaniline (2)**. Product was prepared according to literature procedures with slight modifications (38). To a solution of 4-ethynylaniline (1.003 g, 8.56 mmol) and cesium carbonate (8.345 g, 25.6 mmol) in dry DMF (50 ml) under nitrogen, methyl iodide was added drop wise over 15 min (1.6 ml, 26.0 mmol). The reaction was heated to 40°C for 24 h and monitored by TLC (Hex:EtOAc). The reaction was cooled and diluted with H_2_O (100 ml) and extracted with EtOAc (3 × 30 ml). The organic layers were combined and washed with H_2_O (3 × 10 ml) and finally with brine solution. The organic layer was dried over MgSO_4_ and reduced to dryness. Product was purified by column chromatography (SiO_2_, Hex:EtOAc, 9:1) as a colourless liquid (383 mg, 2.64 mmol, 31%). ^1^H NMR (400 MHz, CDCl_3_) δ: 7.39 (dt, 2H), 6.65 (dt, 2H), 3.00 (s, 7H). The ^1^H NMR spectrum was in agreement with literature data (38).

***N*-Boc-3-ethynylaniline (3)**. The compound was prepared according to reported methods (40). To a solution of 3-ethynylaniline (5.040 g, 43.02 mmol) in dry THF (80 ml), di-*tert*-butyl-dicarbonate (10.112 g, 46.33 mmol) in THF (10 ml) was added dropwise. Reaction was refluxed for 18 h and monitored by TLC. Solvent was reduced under pressure and the resulting material was purified by column chromatography (Hex:EtOAc) to yield the product as a colourless liquid (8.786 g, 40.44 mmol, 94% yield). ^1^H NMR (600 MHz, CDCl_3_) δ: 7.37 (d, *J* = 7.3 Hz, 1H), 7.24 (t, *J* = 7.8 Hz, 1H), 7.17 (dt, *J* = 7.6, 1.3 Hz, 1H), 6.58 (s, 1H), 3.06 (s, 1H), 1.53 (s, 9H). ^1^H NMR spectrum was in agreement with literature data (41).

**Boc-3-ethynyl*-N-*methylaniline (4)**. The target ligand was prepared by previously reported methods with slight modifications (42). Sodium hydride (3 eq, 14.8 mmol) was prepared in the reaction vessel under nitrogen flux and washed with dry hexane, suspended in dry DMF (40 ml) over ice. A solution of boc-3-ethynylaniline (1.038 g, 4.93 mmol) in DMF (10 ml) was added slowly over 30 min to the reaction under a nitrogen atmosphere and the resulting solution was allowed to stir at rt for 2 h. Methyl iodide (1.54 ml, 5 eq, 24.7 mmol) was added to the reaction slowly over 5 min and reaction left stirring for 4 h. The reaction was quenched slowly with H_2_O at 0°C. The solution was diluted with H_2_O (50 ml) and extracted with EtOAc (4 × 30 ml). The organic layers were combined and further washed with H_2_O (5 × 10 ml) and finally with brine. The organic layer was dried over MgSO_4_, filtered and solvents removed by rotary evaporation. The crude product was purified by column chromatography (SiO_2_, 9.5:0.5 Hex:EtOAc) as an off white solid (850 mg, 3.68 mmol, 75%). ^1^H NMR (400 MHz, CDCl_3_) δ: 7.38 (q, *J* = 1.4 Hz, 1H), 7.31–7.26 (m, 3H), 3.27 (s, 3H), 3.09 (s, 1H), 1.47 (s, 9H). The ^1^H NMR spectrum was in agreement with literature data (42).

### Boc-Click reactions

**Tri-Click boc-3-ethynylaniline (5)**. To a solution of triazide (0.285, 1.00 mmol) and DIPEA (0.174 ml, 1.00 mmol) in degassed ACN (20 ml), CuBr (0.144 g, 1.00 mmol) was added slowly under nitrogen atmosphere. The solution was stirred for 15 min and boc-3-ethynylaniline (**3**, 0.679 g, 3.13 mmol) was added dropwise as a solution in ACN (5 ml) to the reaction. The reaction was refluxed for 50°C for 48 h, until the complete conversion was observed by TLC. The reaction was allowed to cool and ACN removed by reduced pressure. The resulting crude material was suspended in 0.1 M EDTA solution (pH 8) and heated to reflux for 1 h. The solution was allowed to cool and extracted with DCM (3 × 50 ml). The organic layers were combined and washed with H_2_O (3 × 30 ml) and brine. The organic solution was dried over MgSO_4_ and reduced to dryness. The crude product was column purified (SiO_4_ DCM:MeOH, 9:1) resulting in the title product as cream solid (505 mg, 0.538 mmol, 54%). ^1^H NMR (400 MHz, CDCl_3_) δ: 7.77 (s, 3H), 7.57 (s, 3H), 7.51 (dd, *J* = 7.6, 1.4 Hz, 3H), 7.41 – 7.37 (m, 3H), 7.31 (t, *J* = 7.9 Hz, 3H), 6.80 (s, 3H), 5.74 (s, 6H), 2.49 (s, 9H), 1.52 (s, 27H). Amine deprotection detailed below.

**Tri-Click boc-3-ethynyl-*N*-methylaniline (6)**. To a solution of triazide (0.286, 1.00 mmol) and DIPEA (0.174 ml, 1.00 mmol) in degassed ACN (20 ml), CuBr (0.144 g, 1.00 mmol) was added slowly under nitrogen atmosphere. The solution was stirred for 15 minutes and boc-3-ethynyl-*N*-methylaniline (**4**, 0.718 g, 3.11 mmol) was added dropwise as a solution in ACN (5 ml) to the reaction. The reaction was refluxed for 18 h, until the complete conversion was observed by TLC. The reaction was allowed to cool and ACN removed by reduced pressure. The resulting crude was suspended in 0.1 M EDTA solution (pH 8) and heated to reflux for 1 h. The solution was allowed to cool and extracted with DCM (3 × 50 ml). The organic layers were combined and washed with H_2_O (3 × 30 ml) and brine. The organic solution was dried over MgSO_4_ and reduced to dryness. The crude product was column purified (SiO_4_ DCM:MeOH, 9:1) and resulted in a white solid (866 mg, 0.88 mmol, 88%). ^1^H NMR (400 MHz, CDCl_3_) δ: 7.74 (t, *J* = 1.9 Hz, 3H), 7.55 (d, *J* = 10.4 Hz, 6H), 7.35 (t, *J* = 7.8 Hz, 3H), 7.22 (ddd, *J* = 8.0, 2.3, 1.1 Hz, 3H), 5.75 (s, 6H), 3.29 (s, 9H), 2.54 (s, 9H), 1.47 (d, *J* = 2.8 Hz, 27H). Amine deprotection procedure detailed below.

### Tri-Click reactions

**Tri-Click propargylamine (TC1)**. To a solution of triazide (0.214 g, 0.75 mmol) in degassed *t*-BuOH:H_2_O (1:1, 6 ml), propargylamine (147 μl, 2.29 mmol) and Na-l-ascorbate (5%, 0.15 mmol) was added and stirred under a nitrogen atmosphere for 15 min, prior to the addition of a solution of CuSO_4_ (1%, 0.03 mmol) in *t*-BuOH:H_2_O (1:1, 2 ml) which was added dropwise over 10 min. The reaction was stirred at 25°C for 18 h. The solvent was reduced to 3 ml under a stream of nitrogen and cooled on ice. The resulting precipitate was collected by vacuum filtration and washed with ice cold EDTA (0.1 M, 3 × 8 ml) and ice-cold diethyl ether (3 × 10 ml). A yellow solid was recovered in good yield, (284 mg, 0.63 mmol, 84%). MP: 119–120°C. ^1^H NMR (600 MHz, D_2_O) δ: 7.66 (s, 3H), 5.64 (s, 6H), 3.77 (s, 6H), 2.21 (s, 9H). ^13^C NMR (151 MHz, D_2_O) δ: 148.32, 140.12, 130.00, 122.69, 49.03, 35.46, 15.51. IR (ATR, cm^–1^): 3124, 2982, 1666, 1603, 1442, 1381, 1328, 1215, 1120, 1046, 949, 802. ESI-MS *m*/*z*: [M + H]^+^ Cald for C_21_H_31_N_12_^+^: 451.56; found 451.6.

**Tri-Click *N-*methyl-propargylamine (TC2)**. To a solution of triazide (0.430 g, 1.5 mmol) in degassed *t*-BuOH:H_2_O (1:1, 8 ml), *N-*methyl-propargylamine (380 μl, 4.52 mmol) and Na-l-ascorbate (10%, 0.45 mmol) was added and stirred under a nitrogen atmosphere for 15 min. A solution of CuSO_4_ (1%, 0.045 mmol) in *t*-BuOH:H_2_O (1:1, 2 ml) was added dropwise over 10 min and stirred at 25°C for 18 h. The solvent was removed under reduced pressure and the product was suspended in 0.1 M EDTA solution (pH 8) and extracted with DCM (3 × 50 ml). The organic layers were combined and washed with ice cold H_2_O (3 × 20 ml) and brine (3 × 30 ml). The organic solution was dried over MgSO_4_ and solvent removed under reduced pressure. A white solid was recovered (0.548 g, 1.13 mmol, 74%). MP: 197–199°C. ^1^H NMR (600 MHz, CDCl_3_) δ: 7.24 (s, 3H), 5.63 (s, 6H), 3.82 (s, 6H), 2.44 (s, 9H), 2.38 (s, 9H).^13^C NMR (151 MHz, CDCl_3_) δ: 146.83, 139.73, 130.66, 120.91, 48.88, 46.84, 36.16, 16.57. IR (ATR, cm^–1^): 3066, 2981, 1634, 1550, 1447, 1380, 1331, 1261, 1207, 1131, 1047, 811. ESI-MS *m*/*z*: [M + H]^+^ Cald for C_24_H_36_N_12_^+^: 493.33; found 493.2.

**Tri-Click *N,N*-dimethyl-propargylamine (TC3)**. To a solution of triazide (0.286 1.00 mmol) and DIPEA (0.174 ml, 1.00 mmol) in degassed ACN (20 ml), CuBr (0.144 g, 1.00 mmol) was added slowly under nitrogen atmosphere. The solution was stirred for 15 min and *N,N*-dimethyl-propargylamine (0.340 ml, 3.16 mmol) was added dropwise as a solution in ACN (5 ml) to the reaction. The reaction was refluxed for 72 h, until the complete conversion was observed by TLC. The reaction was allowed to cool and ACN removed by reduced pressure. The resulting mixture was suspended in 0.1 M EDTA solution (pH 8) and heated to reflux for 1 h. The solution was allowed to cool and extracted with DCM (3 × 50 ml). The organic layers were combined and washed with H_2_O (3 × 20 ml) and brine (3 × 30 ml). The organic solution was dried over MgSO_4_ and solvent removed under reduced pressure. The resulting product was column purified (SiO_4_ DCM:MeOH, 9:1) and isolated as an orange solid (338 mg, 0.63 mmol, 63%). MP: 227–229°C. ^1^H NMR (600 MHz, (CD_3_)_2_SO) δ: 7.75 (s, 3H), 5.67 (s, 6H), 3.43 (s, 6H), 2.39 (s, 9H), 2.09 (s, 18H). ^13^C NMR (151 MHz, (CD_3_)_2_SO) δ: 144.10, 139.56, 131.42, 123.66, 54.02, 48.75, 45.04, 16.71. Anal. Cal. for C_27_H_42_N_12_: C, 60.65; H, 7.92; N, 31.43. %Found: C 59.34, H 7.72, N 30.45. IR (ATR, cm^–1^): 3111, 3070, 2977, 2939, 2814, 2762, 1455, 1380, 1336, 1297, 1255, 1213, 1174, 1135, 1037, 1016, 840, 812, 798, 704. ESI-MS *m*/*z*: [M]^+^ Cald for C_27_H_42_N_12_: 534.7; found 535.3.

**Tri-Click 3-ethynylaniline (TC4)**. The procedure was adapted from the literature (42). To a solution of Tri-Click boc-3-ethynylaniline (**5**) (0.505 mg, 0.539 mmol) in DCM (10 ml) TFA (0.615 μl, 8 mmol) was added slowly over ice. The solution was allowed to stir over ice and monitored by TLC to ensure complete removal of Boc group. The solvent and excess TFA were removed under a stream of nitrogen, residue was dissolved in HCl (aq) (1.0 M, 10 ml) and washed with DCM (3 × 20 ml). DCM (20 ml) and water (20 ml) was added to the aqueous fraction and the pH was adjusted to 8 with NaOH (1 M) while stirring. The organic fraction was collected, and the aqueous layer was extracted with DCM (2 × 10 ml). The combined organic fractions were dried (MgSO_4_), filtered and the solvent was removed under reduced pressure. The resulting product was column purified (SiO_4_ DCM:MeOH, 9:1) to afford a white solid (0.295 g, 0.464 mmol, 86%). MP: 228–230°C. ^1^H NMR (600 MHz, (CD_3_)_2_SO) δ: 8.24 (s, 3H), 7.12 (t, *J* = 2.0 Hz, 3H), 7.06 (t, *J* = 7.8 Hz, 3H), 6.97 (dt, *J* = 7.5, 1.2 Hz, 3H), 6.54 (ddd, *J* = 8.0, 2.4, 1.1 Hz, 3H), 5.74 (s, 6H), 3.35 (s br, 6H), 2.48 (s, 9H). ^13^C NMR (151 MHz, (CD_3_)_2_SO) δ: 157.87, 157.66, 148.37, 146.77, 139.40, 131.11, 130.81, 129.27, 120.62, 113.88, 113.57, 110.82, 48.59, 16.42.IR (ATR, cm^–1^): 3347, 1674, 1611, 1589, 1430, 1182, 1123, 1045, 836, 802, 782, 722, 691. ESI-MS *m*/*z*: [M + H]^+^ Cald for C_36_H_37_N_12_^+^: 637.3; found 637.3.

**Tri-Click 3-ethynyl-*N*-methylaniline (TC5)**. The removal of the protecting group was carried as stated above. Briefly, to a solution of Tri-Click boc-3-ethynyl-*N*-methylaniline (**6**) (0.850 g, 0.87 mmol) in DCM (15 ml) TFA (1 ml, 13.05 mmol) was added slowly over ice. The solution was allowed to stir over ice and monitored by TLC to ensure complete removal of Boc group. No alterations were made to the workup as detailed previously. The resulting product was column purified (SiO_4_ DCM:MeOH, 9:1). Solvent was removed under reduced pressure and foamed with THF to afford an extremely hygroscopic white solid (0.366 g, 0.539 mmol, 62%). Product is stored under argon. ^1^H NMR (600 MHz, (CD_3_)_2_SO) δ: 8.31 (s, 3H), 7.17 – 7.13 (m, 6H), 7.08 (dt, *J* = 7.6, 1.3 Hz, 3H), 6.59 (ddd, *J* = 8.0, 2.4, 1.0 Hz, 3H), 5.75 (s, 6H), 2.73 (s, 9H), 2.49 (s, 9H). ^13^C NMR (151 MHz, (CD_3_)_2_SO) δ: 146.81, 139.50, 131.32, 130.87, 129.40, 120.87, 119.79, 117.83, 115.86, 114.22, 113.89, 112.54, 109.16, 48.63, 30.42, 16.50, 1.17. IR (ATR, cm^–1^): 3112, 1670, 1435, 1177, 1123, 837, 7800, 722, 710, 599, 518. ESI-MS *m*/*z*: [M + H]^+^ Cald for C_39_H_43_N_12_^+^: 679.4; found 679.4. Note: hygroscopic, requires storage under argon

**Tri-Click 3-ethynyl-*N*,*N*-dimethylaniline (TC6)**. To a solution of triazide (0.286, 1.00 mmol) and DIPEA (0.174 ml, 1.00 mmol) in degassed ACN (20 ml), CuBr (0.144 g, 1.00 mmol) was added slowly under nitrogen atmosphere. The solution was stirred for 15 min and 3-ethynyl-*N*,*N*-dimethylaniline (**1**, 0.450 g, 3.09 mmol) was added dropwise as a solution in ACN (5 ml) to the reaction. The reaction was heated to 50°C under nitrogen for 24 h, until complete conversion was observed by TLC. The reaction was allowed to cool and ACN removed by reduced pressure. The resulting crude was suspended in 0.1 M EDTA solution (pH 8) and heated to reflux for 1 h. The solution was allowed to cool and extracted with DCM (3 × 50 ml). The organic layers were combined and washed with H_2_O (3 × 30 ml) and brine. The organic solution was dried over MgSO_4_ and reduced to dryness. The crude product was column purified (SiO_4_ DCM:MeOH, 9:1) and isolated as beige powder (399 mg, 0.52 mmol, 52%). MP: 259–260°C. ^1^H NMR (600 MHz, CDCl_3_) δ: 7.46 (s, 3H), 7.29 (dd, *J* = 2.7, 1.5 Hz, 3H), 7.21 (t, *J* = 8.2 Hz, 3H), 6.95 (ddd, *J* = 7.5, 1.5, 0.9 Hz, 3H), 6.69 (ddd, *J* = 8.4, 2.7, 0.9 Hz, 3H), 5.71 (s, 6H), 2.98 (s, 18H), 2.51 (s, 9H). ^13^C NMR (151 MHz, CDCl_3_) δ: 151.13, 148.74, 139.96, 131.00, 130.98, 129.58, 118.98, 114.19, 112.69, 109.75, 49.21, 40.78, 16.96. IR (ATR, cm^–1^): 1605, 1582, 1497, 1442, 1350, 1223, 1182, 1042, 986, 857, 780, 692, 461. Anal. Cal. for C_42_H_48_N_12_: C, 69.97; H, 6.71; N, 23.31. %Found: C, 69.30; H, 6.66; N, 22.70. ESI-MS m/z: [M + H]^+^ Cald for C_42_H_49_N_12_^+^: 721.4; found 721.4.

**Tri-Click 4-ethynyl-*N*,*N*-dimethylaniline (TC7)**. To a solution of triazide (0.286, 1.00 mmol) and DIPEA (0.174 ml, 1.00 mmol) in degassed ACN (20 ml), CuBr (0.144 g, 1.00 mmol) was added slowly under nitrogen atmosphere. The solution was stirred for 15 min and 4-ethynyl-*N*,*N*-dimethylaniline (**2**, 0.454 g, 3.13 mmol) was added dropwise as a solution in ACN (5 ml). The reaction was refluxed for 72 h, until the complete conversion was observed by TLC. The reaction was allowed to cool and ACN removed by reduced pressure. The resulting crude was suspended in 0.1 M EDTA solution (pH 8) and heated to reflux for 1 h. The solution was allowed to cool and extracted with DCM (3 × 50 ml). The organic layers were combined and washed with H_2_O (3 × 30 ml) and brine. The organic solution was dried over MgSO_4_ and reduced to dryness. The crude product was column purified (SiO_4_ DCM:MeOH, 9:1) and isolated as a cream solid (249 mg, 0.345 mmol, 35%). MP: 284–286°C. ^1^H NMR (600 MHz, (CD_3_)_2_SO) δ: 8.09 (s, 3H), 7.63 (d, *J* = 8.8 Hz, 6H), 6.71 (d, *J* = 8.9 Hz, 6H), 5.73 (s, 6H), 2.91 (s, 18H), 2.47 (s, 9H).^13^C NMR (151 MHz, (CD_3_)_2_SO) δ: 150.41, 147.27, 139.75, 131.35, 126.58, 119.39, 119.02, 112.66, 49.00, 16.84. ATR-IR (cm^–1^): 2851, 1619,1559, 1443,1348, 1220, 1170, 1040, 947, 812, 695, 533. ESI-MS *m*/*z*: [M + H]^+^ Cald for C_42_H_49_N_12_^+^: 721.4; found 721.4.

### Investigation of the para-substituted 4-ethynylanilines

***N*-Boc-4-ethynylaniline (7)**. The compound was prepared according to literature procedures (43,44). To a solution of 4-ethynylaniline (5 mmol) in dry THF (20 ml), di-tert-butyl dicarbonate (15 mmol) was added dropwise. Reaction was refluxed under nitrogen for 18 h. Monitored by TLC (Hex:EtOAc 9:1) until the complete conversion of product was observed. The reaction was allowed to cool to rt and solvent removed under reduced pressure. Crude yellow oil was subjected to column chromatography (SiO_2_, Hex:EtOAc, 9.5:0.5–9:1) to yield the pure product as a colourless liquid in high yield (0.98 g, 4.51 mmol, 90% yield). ^1^H NMR (400 MHz, CDCl_3_) δ: 7.47–7.31 (m, 4H), 6.64 (s, 1H), 3.04 (s, 1H), 1.53 (s, 9H). The ^1^H NMR spectra was in agreement with literature data (44).

### Tri-Click non-amine controls

**Tri-Click 4-bromo-1-butyne (TC-Br)**. To a solution of triazide (0.100 g, 0.35 mmol) in 10 ml THF:H_2_O (1:1), Na-l-ascorbate (5%) was added followed by CuSO_4_ (1%) under a nitrogen atmosphere. To this solution 4-bromo-1-butyne (0.1 ml, 1.05 mmol) was added. The reaction was allowed to stir at rt for 24 h, a white precipitate was observed after 8 h. On the completion of the reaction, the white precipitate was collected by vacuum filtration and washed with diethyl ether to afford the title compound as a white solid (0.239 g, 0.35 mmol, 85%). MP: decomposed at 269°C. ^1^H NMR (400 MHz, CDCl_3_) δ: 7.10 (s, 3H), 5.58 (s, 6H), 3.56 (t, *J* = 6.7 Hz, 6H), 3.17 (t, *J* = 6.7 Hz, 6H), 2.35 (s, 9H). ^13^C NMR (151 MHz, CDCl_3_) δ: 144.91, 139.70, 130.71, 121.10, 77.24, 77.03, 76.82, 48.93, 31.81, 29.31, 29.28, 16.67, 16.63. Anal. Cal. for C_24_H_30_Br_3_N_9_: C, 42.13; H, 4.42; N, 18.42; Br, 35.03. %Found: C, 42.41; H, 4.38; N, 18.50; Br, 34.65. IR (ATR, cm^–1^): 3065, 1558, 1441, 1259, 1216, 1147, 1046, 925, 880, 812, 667, 553. ESI-MS *m*/*z*: [M + H]^+^ Cald for C_24_H_31_Br_3_N_9_^+^: 682.0247; found 682.0212. [M + 2H]^+2^ Cald for C_24_H_32_Br_3_N_9_^2+^: 341.5; found 341.5.

**Tri-click 2-methyl-3-butyne-2-ol (TC-iPrOH)**. To a solution of triazide (0.430 g, 1.5 mmol) in THF:H_2_O (1:1, 15 ml), Na-l-ascorbate (10%, 0.45 mmol) was added followed by CuSO_4_ (1%, 0.045 mmol) under a nitrogen atmosphere. To this solution 2-methyl-3-butyne-2-ol (0.382 g, 0.44 ml, 4.52 mmol) was added dropwise. The reaction was stirred at 25°C for 24 h. The resulting precipitate was isolated by vacuum filtration and washed with cold EDTA (0.1 M, 3 × 10 ml) and ice-cold water (3 × 10 ml). A white solid was recovered in good yield, (570 mg, 1.06 mmol, 71%). MP: 274–275°C. ^1^H NMR (600 MHz, (CD_3_)_2_SO) δ: 7.65 (s, 3H), 5.66 (s, 6H), 5.08 (s, 3H), 2.44 (s, 9H), 1.42 (s, 18H). ^13^C NMR (151 MHz, (CD_3_)_2_SO) δ: 156.19, 139.61, 131.48, 120.44, 67.56, 48.67, 31.18, 16.87. Anal. Cal. for C_27_H_39_N_9_O_3_: C, 60.32; H, 7.31; N, 23.45. %Found: C, 58.62; H, 7.26; N, 21.71. IR (ATR, cm^–1^): 3057, 1368, 1216, 1175, 1058, 955, 857,827, 806, 714. ESI-MS m/z: [M + Na]^+^ Cald for C_27_H_39_N_9_O_3_Na^+^: 560.3; found 560.3.

### Polyamine controls

**2,4,6-*Tris*-(aminomethyl)-mesitylene (T1)**. 2,4,6-*Tris*-(aminomethyl)-mesitylene was prepared as per literature (37,45). To a solution of triazide (1.00 g, 2.1 mmol) in absolute EtOH (40 ml), Pd/C (10% w/w, 100 mg) was added and the reaction was stirred under a H_2_ balloon for 6 h. It was then filtered through a small pad of celite, which was rinsed with EtOAc (3 × 15 ml). The filtrate was concentrated to afford triamine (1.186 g, quant.) as a white solid. MP: 149–150°C. ^1^H NMR (600 MHz, CDCl_3_) δ: 3.95 (s, 6H), 2.48 (s, 9H). ^13^C NMR (151 MHz, CDCl_3_) δ: 138.13, 133.48, 40.84, 15.42. Melting point and ^1^H NMR spectra were in agreement with literature data (37,46). ATR-IR (cm^–1^): 3342, 2958, 2903, 2218, 1568, 1448, 1379, 1298, 1050, 861,620. ESI-MS *m*/*z*: [M + H]^+^ Cald for C_12_H_22_N_3_^+^: 208.2; found 208.2.

**2,4,6-*Tris*-(1,2-ethanediamine)-mesitylene (T2)**. The target amine scaffold was prepared by previously reported methods with slight modifications (47). To a solution of 2,4,6-*tris-*(bromomethyl)-mesitylene (1.200 g, 3.0 mmol) in dry THF (50 ml), ethylenediamine (8.04 ml, 120 mmol) was added. The resulting solution was stirred at 25°C until complete conversion was observed by ^1^H NMR (24 h). Solvent and excess diamine were removed under reduced pressure. The resulting oil was solubilized in MeOH (30 ml) and KOH (0.340 g, 6 mmol) was added and the inorganic salts were precipitated by the addition of diethyl ether and removed my filtration. The solvent was removed under reduced pressure and product dried under schlenk line. The resulting amine scaffold was identified as a thick oil (0.673 g, 1.99 mmol, 67%). ^1^H NMR (600 MHz, CDCl_3_) δ: 3.76 (s, 6H), 2.84–2.75 (m, 12H), 2.42 (s, 9H). ^13^C NMR (151 MHz, CDCl_3_) δ: 135.03, 135.00, 52.84, 48.47, 41.68, 15.48. ^1^H NMR was in agreement with literature data (47). ATR-IR (cm^–1^): 3266, 2852, 1651, 1568, 1450, 1338, 1105, 1030, 814, 747. ESI-MS *m*/*z*: [M + H]^+^ Cald for C_18_H_37_N_6_^+^: 337.3; found 337.3.

**2,4,6-*Tris*-(1,3-propanediamine)-mesitylene (T3)**. The target amine scaffold was prepared by previously reported methods with slight modifications (47). To a solution of 2,4,6-*tris-*(bromomethyl)-mesitylene (1.200 g, 3.0 mmol) in dry THF (50 ml) 1,3-diaminopropane (10.02 ml, 120 mmol) was added. The resulting solution was stirred at 25°C until the complete conversion was observed by ^1^H NMR (24 h). The product was worked up as previously stated and the resulting amine scaffold was identified as a thick oil (1.003 g, 2.65 mmol, 88%). ^1^H NMR (600 MHz, CD_3_OD) δ: 3.86 (s, 6H), 2.98 (t, *J* = 7.1 Hz, 6H), 2.89 (t, *J* = 7.0 Hz, 6H), 2.46 (s, 9H), 1.87 (p, *J* = 7.0 Hz, 6H). ^13^C NMR (151 MHz, CD_3_OD) δ: 137.15, 135.46, 40.56, 29.53, 16.46.The ^1^H NMR spectra was in agreement with literature data (47). ATR-IR (cm^–1^): 3256, 2922, 2856, 1567, 1452, 1377, 1327, 1103, 1026, 969, 815, 752. ESI-MS m/z: [M + H]^+^ Cald for C_21_H_43_N_6_^+^: 379.3544; found 379.3542. [M + 2H]^+2^ Cald for C_21_H_43_N_6_^+2^: 190.2; found 190.2. [M + 3H]^+3^ Cald for C_21_H_43_N_6_^+3^ Cald 127.1; found 127.1.

### DNA recognition procedures

#### Preparation of solutions

Tri-Click samples were initially prepared in DMF and further diluted in HEPES buffer (80 mM). Metal complexes of the Tri-Click series were prepared *in-situ* by co-incubating with copper(II) nitrate trihydrate, manganese (II) chloride tetrahydrate or zinc(II) acetate dihydrate for 30 min at 37°C prior to DNA addition.

#### DNA nuclease and condensation studies

Procedures were adapted from previously published protocols, (28) where reactions were carried out in 80 mM HEPES (pH 7.4) unless otherwise stated and followed the general procedure: a total volume of 20 μl with 25 mM NaCl, 400 ng pUC19 at varying concentrations of test compounds (0.25–500 μM) were incubated at 37°C for either 90 min, 6, 12 or 24 h. Reactions were quenched by adding 6× loading buffer (Fermentas) containing 10 mM Tris–HCl, 0.03% bromophenol blue, 0.03% xylene cyanole FF, 60% glycerol, 60 mM EDTA and samples were loaded onto an agarose gel (1.2%) containing 4 μl EtBr. Electrophoresis was run at 70 V for 1 h in 1× TAE buffer. DNA studies of TC1 (2.5–10 μM) and various ratios of copper (Cu(II):TC1 0:1–8:1) were conducted in the presence of reductant were supplemented with 1 mM Na-l-ascorbate and incubated at 37°C for 30 min. Electrophoresis was carried out at 70 V for 90 min in 1× TAE buffer and photographed using a UV transilluminator.

### DNA damage studies of Cu(II)–TC1

#### Competitive ethidium bromide displacement assay

The DNA binding affinity of TC1 was determined over a 2 h time period using calf-thymus DNA and synthetic alternating co-polymers poly[d(A-T)_2_)] and poly[d(G-C)_2_] by ethidium bromide fluorescence quenching in a similar manner to the high throughput method previously reported by Kellett *et al.* (48). TC1 (10 μM) was treated with varying ratios of copper(II) nitrate 0:1–8:1 in ctDNA, after which DNA was treated with 3:1 Cu(II)–TC1 at various concentrations. Each drug concentration was measured in triplicate, on at least two separate occasions, and the apparent binding constants were calculated using *K*_app_ = *K*_e_ × 12.6/C_50_ where *K*_e_ = 9.5 × 10^6^ M^−1^.

#### High resolution ESI-MS

Accurate mass spectrometry analyses were conducted using a MaXis HD quadrupole ESI-QTOF mass spectrometer. In a final volume of 1 ml, a solution of TC1 (4 mM) was taken and mixed with various equivalents of copper(II) nitrate trihydrate (0–32 mM) to give a series of Cu(II):TC1 solutions ranging between 0:1 and 8:1 mM. Further dilution of these samples were made as required to perform ESI-MS analysis. The relative intensities of peaks in each pattern were combined and expressed as a percentage of the total relative intensity given by the three metal complex fragmentation patterns that were identified. Analyses were performed in ESI positive mode with the capillary voltage was set to 4500 V, nebulizing gas at 0.6 bar, drying gas at 4 l/min at 180°C in each case. The TOF scan range was from 75 to 1600 mass-to-charge ratio (*m/z*). Data processing was performed using the Compass Data Analysis software version 4.3 (Bruker Daltonik GmbH, Bremen, Germany).

#### Atomic force microscopy (AFM)

AFM samples were prepared according to previously reported methods(28) and follow the general procedure detailed below in the presence and absence of exogenous reductant. *In the presence of reductant:* A total volume of 20 μl containing pUC19 (200 ng), MgCl_2_ (10 mM), varying concentrations of test compound Cu(II)–TC1 (0.5, 1.0, 7.5 and 10 μM) in the presence of Na-L-ascorbate (1 mM) were incubated for 25 min at 37°C. Samples were further diluted to a DNA concentration of 2–3 ng/μl in a final volume of 10 μl with nuclease free H_2_O, pipetted directly onto freshly cleaved mica and incubated for a further 5 min. *In the absence of reductant*: A total volume of 10 μl final concentrations of 3 ng/μl of pUC19 (NEB, N3041), 5 mM MgCl and varying concentrations of test compound Cu(II)–TC1 (5, 10 and 30 μM) samples were incubated at 37°C for 1 h. Mica was freshly cleaved prior to incubating with samples (10 μl, 5 min). All samples were rinsed thoroughly with nuclease free water (500 μl) and dried under compressed air. AFM images were acquired in ambient air with a commercial microscope, in tapping-mode, using super sharp silicon cantilevers with a 40 N/m force constant. Topographic images were recorded at a scanning rate of >1 Hz, and a resonance frequency of about 300 kHz (nominal value). Images were processed using the WSxM software (49).

#### DNA cleavage in the presence of ROS scavengers

The assay was conducted according to recently reported methods (19,50). Briefly, to a final volume of 20 μl, 80 mM HEPES, 25 mM NaCl, 1 mM Na-l-ascorbate, and 400 ng of pUC19 DNA were treated with varying drug concentrations Cu(II)–TC1 (2.5–10.0 μM) in the presence ROS scavengers; sodium azide (NaN_3,_ 10 mM), 4,5-dihydroxy-1,3-benzenedisulfonic acid (tiron, 10 mM), d-mannitol (10 mM), *N,N′-*dimethylthiourea (DMTU, 10 mM), and l-methionine (10 mM). Solutions were briefly vortexed and incubated at 37°C for 30 min and electrophoresis was carried out at 70 V for 1 h in 1× TAE.

#### DNA cleavage in the presence of repair enzymes

The assay was conducted according to methods recently reported by Fantoni *et al.* (19) with slight modifications. Supercoiled pUC19 DNA (400 ng) in 4 mM HEPES, 25 mM NaCl, 1 mM Na-l-ascorbate in a final volume of 20 μl nuclease free H_2_O, was pre-incubated with Cu(II)–TC1 for 30 min at 37°C at 2.5–10.0 μM. The following reactions were supplemented with associated buffers as per manufacturer's recommendations. Repair enzymes Fpg, endonuclease (Endo) III, Endo IV, Endo V and hAAG (2 units) were added to the reaction mixture and incubated for 30 min at 37°C. Samples were denatured with 0.25% SDS, 250 μg/ml proteinase K and heated to 50°C for 20 min. Reactions were quenched by 6× loading buffer, loaded onto agarose gels and subjected to electrophoresis as stated above.

### Intracellular DNA damage

The protocol was adapted from Singh *et al.* (51).

#### Blood sample collection

Blood samples from healthy volunteers without having any pathology were collected from the Hematology Lab at the Clinical Chemistry Department at Sahlgrenska University Hospital, Gothenburg, Sweden. Gradient centrifugation using Lymphoprep (Axis-Shield PoC AS, Oslo, Norway) was used to collect peripheral mononuclear blood cells (PBMCs).

#### Treatment of PBMCs with TC1 and copper (II) nitrate trihydrate

100 mM stock solutions of TC1 and copper (II) nitrate trihydrate was prepared in DMSO and Milli Q^®^ (MQ) water respectively and stored at -20°C until further use. Co-incubation of TC1 at three different concentrations, 75, 100 and 300 μM with a Cu(II) concentration of 300 μM was performed for 30 min at 37°C in 1× RPMI. 5 × 10^5^ PBMCs were introduced to the Cu(II)–TC1 complex and incubated for 1 h on a thermal block at 37°C. Final DMSO concentrations did not exceed 0.1% (v/v).

#### Treatment of PBMCs with antioxidants

100 mM stock solutions of sodium pyruvate, tiron, l-histidine, and d-mannitol were prepared in MQ water. Cells were pre-treated with 1 mM scavengers for 2 h prior to Cu(II)–TC1 exposure.

#### Extraction of DNA

After drug treatment and antioxidant studies, DNA was extracted using GenElute-Mammalian Genomic DNA Miniprep Kit and eluted in 10 mM Tris–Cl (pH 8.5). DNA concentrations were measured using a NanoDrop 1000 spectrophotometer. Care was taken to avoid loss of genomic integrity by using wide-bore tips.

#### Fluorescent labeling of Cu(II)–TC1 induced DNA damage

DNA (100 ng) was incubated with APE1, Fpg or Endo III (2.5 U) in 1× CutSmart Buffer for 1 h at 37°C. The *in vitro* DNA repair was followed by 1 h incubation at 20°C with dNTPs (1 μM of dATP, dGTP, dCTP, 0.1 μM dTTP and 0.1 μM aminoallyl-dUTP-ATTO-647N, 1× NEBuffer 2 and DNA polymerase 1 (2.5 U) at 20°C. Subsequently, the reaction was terminated with 2.5 μl of 0.25 M EDTA.

#### Silanization of coverslips

Two silane molecules (3-aminopropyl) triethoxysilane (APTES), allyltrimethoxysilane (ATMS), and acetone were used to silanize standard 22 × 22 mm coverslips (52). The coverslips were carefully put into an acetone solution containing 1% APTES and 1% ATMS (v/v) and coated for 1 h. Coated coverslips were rinsed with acetone and MQ water to remove residues. The air dried silanized coverslips were stored in a parafilm sealed petri dishes and used within a week.

#### Staining and instrument

The Aminoallyl-dUTP-ATTO-647N labelled DNA samples were diluted with 0.5× TBE and were stained with 320 nM YOYO-1 (Invitrogen). 2 μl β-mercaptoethanol was added in total volume of 100 μl. The DNA samples were extended by placing the 3.8 μl solution at the interface of the silanized coverslip and a microscopy slide (VWR Frosted). Zeiss Observer.Z1, equipped with an Andor iXON Ultra EMCCD camera and a Colibri 7 LED illumination system was used to obtain the fluorescence images. Each image consisted of two colours, YOYO-1 (green), and aminoallyl-dUTP-ATTO-647N (red) using appropriate filters band-pass excitation filters (475/40 and 640/30 nm) and bandpass emission filters (530/50 and 690/50 nm), respectively with exposure times of 10 ms and 500 ms.

### Software analysis

AFM images were processed using the WSxM software to remove the background slope and normalize the z-scale across all images, no additional filtering was performed (49). Microscopy images were analysed using a custom-made software. The total number of colocalized aminoallyl-dUTP-ATTO-647N labels in an image set was divided by the total DNA length in pixels to get the ratio as DNA damage/length. The final values were depicted as Dots/MBp. It was calculated by stretching lambda DNA molecules (48 502 bp) in similar buffered conditions where we observed that 1 μm = ∼3000 bp. Band densitometry was quantified using the Image J software package were each reaction was carried out in triplicate.

### Statistics

Intracellular DNA damage and band densitometry studies were performed in triplicate and analyzed in GraphPad Prism using un-paired *T* tests. The data was considered statistically significant with *P ≤ 0.05; **P ≤ 0.01; ***P ≤ 0.001.

## RESULTS AND DISCUSSION

### Tri-Click ligand development

**Figure 1.**
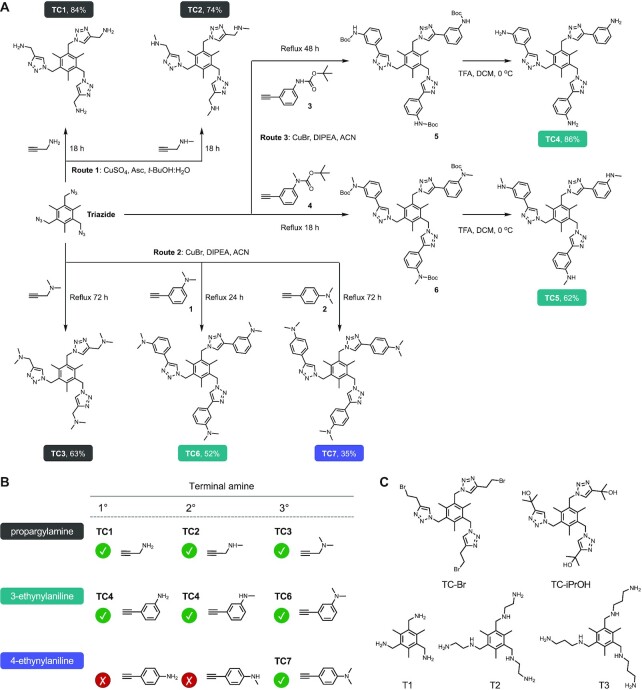
(**A**) General synthetic routes for the Tri-Click series. **Route 1**: alkyne functionalised primary and secondary amines were coupled to triazide using the CuAAC reaction. **Route 2**: tertiary amines where clicked with Cu(I). **Route 3**: Cu(I) click conditions of Boc-protected benzyl substituted amines followed by deprotection. (**B**) Structures of successful and attempted click rections of alkyne-amines. (**C**) Tri-Click SAR controls. Clicked derivatives with no terminal amines TC-Br and TC-*t*-BuOH and simplified polyamine analogues T1-3.

We employed the CuAAC click reaction to generate TC compounds (TC1-7) described in Figure 1A and B. The parent 1,3,5-*tris*(azidomethyl)-2,4,6-trimethylbenzene (triazide) was prepared using 2,4,6-*tris*(bromomethyl)mesitylene in the presence of excess sodium azide (Supplementary Figure S1a). Initial experiments to identify suitable conditions to click alkyne substrates to each site of triazide focused on studying a simple 2-methyl-3-butyn-2-ol substrate with CuSO_4_ (1%) in the presence of ascorbate (10%). This reaction proceeded at room temperature with the product (TC-iPrOH, Figure 1C) precipitating in reasonable yield. Following this reaction, nine alkyne-amines varying from simple propargylamine to aromatic ethynylanilines, were identified as potential substrates (Figure 1B). Each group contains either a primary, secondary or tertiary amine and our overall aim was to develop nine compounds. The first set of TC agents (TC1-3) was prepared using propargylamine (1°), *N*-methylpropargylamine (2°), and *N,N*-methylpropargylamine (3°). For TC1, initial experiments focused on treating triazide with propargylamine using typical catalytic conditions required for click chemistry, including CuSO_4_ or CuBr with added ascorbate but in all cases no product was isolated. Further attempts using anaerobic conditions under reflux and with excess copper also proved unsuccessful. Similar difficulties were encountered during the attempted preparation of TC2, suggesting the use of primary or secondary amines inhibit this particular CuAAC reaction (53). To probe this further, the reaction involving the tertiary *N,N*-methylpropargylamine was investigated and this afforded the desired TC3 click product at the first attempt. Amine protection chemistry was then investigated where Boc-protected propargylamine and *N*-methylpropargylamine were examined in the CuAAC reaction with triazide. These reactions were successful but required purification by column chromatography and subsequent difficulties were encountered in the removal of the Boc-protecting groups from both products. In keeping with the scope of this study, the click reaction was revisited using a broader range of conditions. The solvent mixture was changed from THF to *t*-BuOH—a more polar protic solvent used in the first click chemistry reactions reported by Sharpless (30)—and the preparation of TC1 and TC2 was then achieved with propargylamine and *N*-methylpropargylamine. These reaction conditions were further optimised through use of a *t*-BuOH:H_2_O (1:1) solvent mixture (supplementary section S-1).

The next group of alkyne-amines investigated was the *ortho*-substituted 3-ethynylanilines (Figure 1B). Similar to the propargylamine series, 3-ethynylaniline (1°) and 3-ethynyl-*N*-methylaniline (2°) did not yield the desired Tri-Click products (TC4 and TC5) but 3-ethynyl-*N*-methylaniline (3°) was successful without the use of protection chemistry and provided a straightforward route to TC6. Ideally, click chemistry reactions should be performed in aqueous conditions without the need for protecting group but since the 1° and 2° ethynylanilines are water insoluble, an organic solvent was required which resulted in the use of Boc-protection chemistry to prepare TC4 and TC5 (Figure 1A, route 3 and Supplementary Figure S1). In this case, deprotection was easily achieved through excess TFA with the desired Tri-Click products isolated by flash chromatography.

To complete the series, the *para*-substituted 4-ethynylanilines (Figure 1B) were investigated as a possible route toward developing the final three compounds. In parallel to earlier observations, 4-ethynyl-*N*,*N*-dimethylaniline (3°)—generated by the methylation of 4-ethynylaniline (Supplementary Figure S1c)—provided a straightforward route for generating TC7 (Figure 1A, route 2). However, 4-ethynylaniline could not be clicked to triazide and so the Boc-protected 4-ethynylaniline was prepared but no conditions—including long reaction times and high catalyst loading—were found to promote the click reaction (Supplementary Figure S2 and Supplementary Table S1). Methylation of the Boc-protected 4-ethynylaniline did not proceed in order to generate 4-ethynyl-*N*-methylaniline and the subsequent 2° Tri-Click product. The final Tri-Click library therefore contained seven compounds: the propargylamine series (TC1-3) that contain simple amine handles linked by one carbon spacer to the triazole ring; the 3-ethynylanilines (TC4-6); and 4-ethynylaniline (TC7). Prior to DNA screening experiments, a series of controls were prepared based on (a) the Tri-Click scaffold lacking an amine handle (TC-Br and TC-iPrOH) and (b) simplified tripodal amines lacking the triazole group (T1–T3) (Figure 1C and Supplementary Figure S3). The purpose of these controls was to ensure any novel nucleic acid interactions observed were not arising from either the amine or the triazole scaffold alone.

### Screening for innate DNA binding interactions

**Figure 2.**
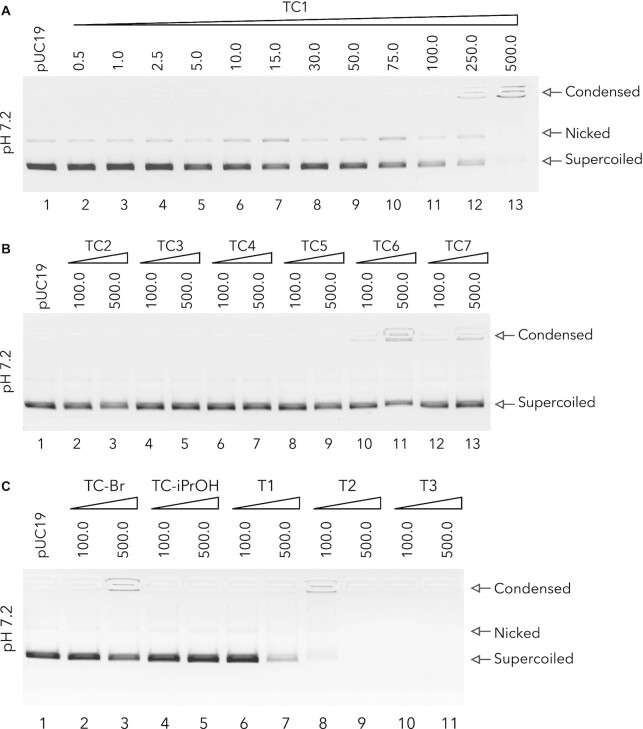
DNA condensation of supercoiled pUC19 exposed to increasing concentrations of (**A**), TC1 (0.5 – 500 μM) (**B**), Tri-Click samples TC2-7 and (**C**), controls at 100 and 500 μM. All reactions were carried out in neutral HEPES buffer (80 mM, pH 7.2) at 37°C for 90 min.

The DNA recognition properties of the TC series, along with control compounds, were probed using electrophoresis experiments with supercoiled pUC19 DNA. TC1 was examined across a wide concentration range and found to condense DNA at high concentrations between 250 and 500 μM (Figure 2A, lanes 11–13). TC2-7 were then examined at high loading only (100 and 500 μM) with slight aggregation effects observed for TC6 and TC7 (Figure 2B, lanes 10–13). The control agent TC-Br had only a mild condensation effect at 500 μM while TC-iPrOH had no effect on pUC19 (Figure 2c). The tripodal amine controls were more active at condensing DNA; T1 was observed to mildly condense pUC19 while T2 and T3 were highly active at 100 μM which was not surprising since both agents can carry a 6+ cationic change and are flexible. Taken together, these results indicate that electrostatic interactions can play a role in DNA aggregation but the TC series are not potent DNA condensation agents compared to simple polyamines or *C*_3_-symmetric opioids (28).

### Nuclease activity in the presence of first row metal ions

**Figure 3.**
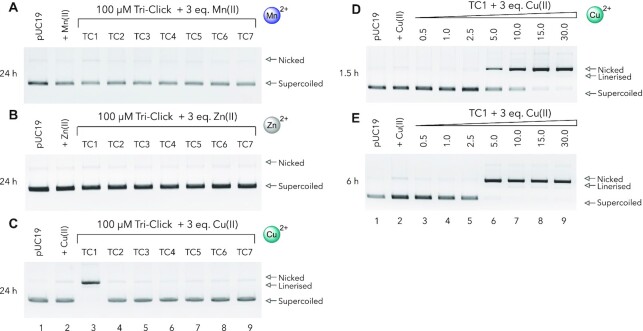
Nuclease activity on supercoiled pUC19. DNA cleavage in the presence of 3 equivalents (**A**), Mn(II), (**B**), Zn(II) and (**C**) Cu(II) over 24 h. Cleavage reactions with increasing concentrations of TC1 with 3 equivalents of copper(II) nitrate over (**D**), 1.5 h and (**E**), 6 h. All reactions were carried out in HEPES (80 mM) with no reductant.

The interactions of the TC series was next examined in the presence of first row metal ions. Given the presence of three potential am(m)ine metal ion binding sites, Tri-Click scaffolds were pre-incubated with three molar equivalents of either Cu(II), Mn(II) or Zn(II) ions—all of which are known to catalyse DNA strand scission when complexed to specific ligands (2,54–56)—prior to incubation with DNA for a period of 24 h at pH 7.2 (Figure 3A–C). Of all the reactions studied, the 3:1 Cu(II):TC1 complex (hereafter referred to as Cu(II)–TC1) was the only complex to display DNA damaging properties (Figure 3C). Here, supercoiled pUC19 was completely relaxed to its open circular form (OC) indicating the formation of single strand breaks (SSBs). Since these reactions were conducted in the absence of exogenous reagents (i.e. added reductant or peroxide) the Cu(II)–TC1 complex appears capable of ‘self-activating’ chemical nuclease activity—an effect previously observed for several polynuclear Cu(II) complexes (22) and mononuclear Cu(II) complexes of marine alkaloids (57,58). To establish the role of am(m)ine coordination in SSB formation, the reactions of TC ligands with Cu(II) were repeated in acidic buffer (pH 4.0) (Supplementary Figure S4). In these conditions the chemical nuclease activity of Cu(II)–TC1 was negligible indicating that metal ion binding, and thus chemical nuclease activity, is inhibited due to the protonation of each primary am(m)ine site in TC1. Finally, the activity of Cu(II)–TC1 was monitored at lower concentrations and shorter timeframes (again in the absence of exogenous reagents) at neutral pH. After 90 min, the presence of 5 μM of Cu(II)–TC1 initiates SSB formation with complete conversion to the nicked form at 50 μM exposure (Figure 3D). Extending the incubation time to 6 h showed that more efficient DNA cleavage could be obtained with 5 μM of TC1 (Figure 3E).

### Copper(II) ion DNA damage dependency

**Figure 4.**
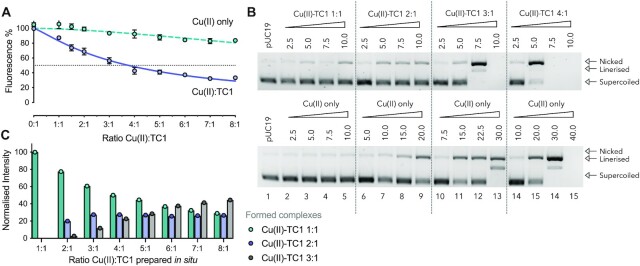
Identification of copper(II) ion DNA recognition and damage dependency. (**A**) Competitive EtBr displacement in ctDNA by mixtures of Cu(II):TC1. The concentration of Cu(II) nitrate was varied against a fixed concentration of TC1 (10 μM). (**B**) Nuclease activity of Cu(II):TC1 and a copper(II) nitrate control. Samples were incubated with supercoiled pUC19 for 30 min in the presence of 1 mM ascorbate. (**C**) Accurate mass spectrometry analyses of Cu(II):TC1 solutions (expressed as normalised percentage of the total relative intensity).

To determine the optimum copper binding ratio required for DNA damage, a series of experiments were designed to identify the DNA binding and nuclease activity at different ratios of Cu(II):TC1 ranging from 1:1 to 8:1. In parallel, high resolution ESI-MS analyses were conducted using the same Cu(II):TC1 ratios. Firstly, DNA binding was examined through an ethidium bromide (EtBr) competitive displacement assay with calf thymus DNA (ctDNA). Here, the percentage displacement of bound EtBr was plotted as a function of Cu(II):TC1 (10 μM TC1) and data were compared to a Cu(II) nitrate control in the absence of TC1 (Figure 4A). It is clear the DNA binding activity of TC1 is dependent on copper(II) with the percentage of bound EtBr diminishing as a function of metal ion concentration. DNA cleavage activity was then investigated using pUC19 plasmid DNA. Here, solutions of Cu(II):TC1 (again ranging between 1:1 to 8:1) were taken and exposed to the plasmid across a 2.5–10 μM [TC1] concentration range in the presence of added ascorbate (Figure 4B). Results were then compared to control experiments of copper(II) nitrate in the absence of TC1. No significant nuclease activity was evident at ratios of 1:1 and 2:1 of Cu(II):TC1. However, higher equivalents of 3:1 and 4:1 Cu(II):TC1 were active with the formation of SSBs and DSBs emerging along with complete degradation at higher concentrations. Complex ratios exceeding 4:1 Cu(II):TC1 displayed rampant DNA degradation (Supplementary Figure S5). Copper-mediated DNA damage in the absence of TC1 revealed significantly lower levels of activity; here, the onset of nicking was detected at 15 μM with higher levels of damage occurring at 30 μM and above. Finally, to identify the predominant Cu(II):TC1 species present at each of the tested ratios, ESI-MS analysis was performed. From the Cu(II):TC1 ratios prepared (1:1 to 8:1), attempts were made to identify three complexes corresponding to mono-cationic forms of mono-, di-, and tri-nuclear species: [Cu(TC1)(NO_3_)]^+^; [Cu_2_(TC1)(NO_3_)_3_]^+^ and [Cu_3_(TC1)(NO_3_)_5_]^+^ respectively. The distribution of these three fragments were then normalised as a percentage of the total relative intensity of all three fragments (Figure 4C and Supplementary Table S2). This analysis revealed simultaneous yet independent 1:1, 2:1, and 3:1 complex formation *in situ* upon increasing addition of copper(II) nitrate. Significantly, this pattern revealed a sequential and proportional transition from an initially predominant mononuclear species to a mixture of complexes dominated by the trinuclear agent. Comparing these results with the activity observed in Figure 4A and B reveals an interesting correlation. The 1:1 complex is inactive and while the emergence of the 2:1 species enhances DNA binding, its DNA damaging properties remain low. Thus, it is not until the 3:1 complex emerges that a potent combination of DNA binding and cleavage activity is produced.

### DNA binding and time-course cleavage interactions

**Figure 5.**
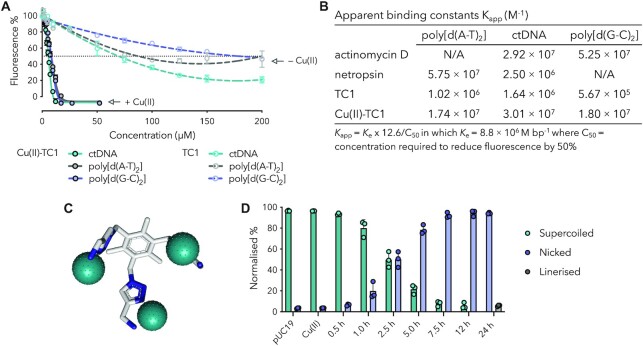
(**A**) Competitive EtBr displacement in canonical dsDNA and alternating co-polymers. (**B**) Apparent DNA binding constants calculated by 50% reduction in EtBr fluorescence (actinomycin D and netropsin values were earlier reported in (59)). (**C**) Proposed simplified structure of Cu(II)–TC1 with 3 equivalents of copper(II) (counterions and charge omitted). (**D**) Band densitometry of Cu(II)–TC1 nuclease activity and three forms of plasmid DNA: supercoiled (form I), SSBs (form II) and DSBs (form III).

To establish how copper(II) binding influences DNA recognition, apparent DNA binding studies were conducted with calf-thymus DNA (ctDNA) along with alternating co-polymers poly[d(A-T)_2_] and poly[d(G-C)_2_]. These experiments determine the indirect binding constant based on the ejection of bound EtBr, which serves as an intercalating reporter. The TC1 ligand alone demonstrated moderate DNA binding constants of ∼10^6^ M^–1^ with ctDNA and A-T duplex polymers while a lower value of ∼10^5^ M^–1^ was observed with poly[d(G-C)_2_]. However, in the presence of three copper(II) nitrate ions, binding increased by 10- and 100-fold (∼10^7^ M^–1^) in all three of the duplexes (Figure 5A–C). Although the binding constants of Cu(II)–TC1 did not show specificity for A–T or G–C rich DNA, they were nonetheless comparable to the high-affinity intercalator actinomycin D and the minor groove binder netropsin (59).

Next, DNA cleavage by Cu(II)–TC1 was mapped using a time-course study (30 min to 24 h) with the Cu(II)–TC1 (25 μM) in the absence of reductant (Figure 5d and Supplementary Figure S6). Band densitometry analysis showed almost 50% SSB formation after 2.5 h and >95% of the plasmid was nicked by 7.5 h. The appearance of double strand breaks (DSBs) could be detected after 24 h but only at limited concentration (∼5%) and after the appearance of SSBs indicating they originate from two independent nicking events. In the absence of reductant, Cu(II)–TC1 (i.e. the 3:1 Cu(II):TC1 complex) clearly produces SSBs but to better understand how this complex might behave biologically, the addition of endogenous reducing agents were employed to mimic physiological conditions. Here, strand scission was catalysed in the presence of the natural reductant, Na-l*-*ascorbate, where SSBs and DSBs occur at low concentration (2.5 μM of TC1) and short reaction times (30 min, Figure 4B), thereby suggesting an oxidative cleavage mechanism is at play. Another reducing agent, 3-mercaptopropionic acid (MPA), (60,61) was examined under identical conditions, however no apparent cleavage of the plasmid was observed (data not shown). This phenomenon has previously been observed where exchanging redox initiators strongly influences DNA cleavage (61,62).

### Atomic force microscopy (AFM) analysis of DNA damage

**Figure 6.**
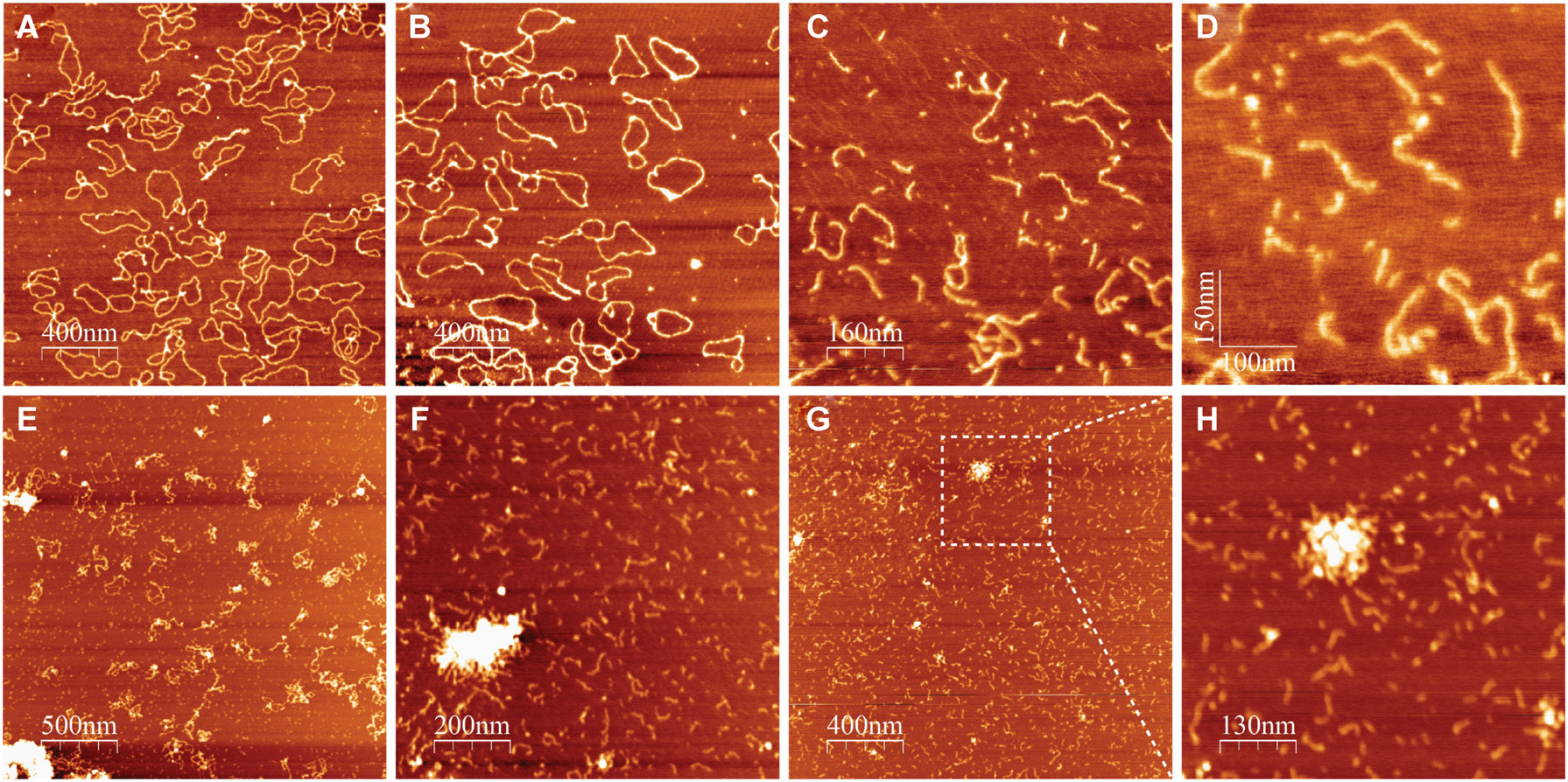
Atomic force microscopy (AFM) images of Cu(II)–TC1 treated supercoiled pUC19 (3 ng/μl) in the presence of reductant (**A**–**D**: 0.5, 1.0, 7.5 and 10 μM of Cu(II)–TC1) and absence of reductant (**E**–**G**: 5, 10 and 30 μM of Cu(II)–TC1; **H**: zoom of G).

Using gel electrophoresis we have shown that Cu(II)–TC1 is capable of cleaving supercoiled plasmid DNA to open circular and linear forms via SSB and DSB formation. To study this conformational change in more detail, AFM measurements were undertaken with pUC19 exposed to Cu(II)–TC1. In the presence of excessive reductant (Na-l-ascorbate), DNA remained predominantly in its supercoiled state, however the addition of 500 nM of Cu(II)–TC1 initiated single strand breaks that were visualised (Figure 6A). Points where ligand-DNA binding occur were identified as increases in the height profile of circular structures (Figure 6B). An increase in complex concentration (1.0 μM) rendered plasmid DNA predominantly in its open circular form (Figure 6B) with complete degradation reached at 7.5–10 μM exposure (Figure 6C and D). DNA damage was also visualised in the absence of reductant at a low concentration (5 μM) of the complex (Figure 6E). Small compact clusters of fragmented DNA were intermediately observed and these small aggregates are evident in Figure 6F and G where the complex concentration (again in the absence of reductant) was increased to 10 and 30 μM, respectively. On closer inspection of the cluster present in Figure 6G, it can be clearly seen that small portions of fragmented DNA surround the compact particle (Figure 6H). These compacted DNA fragments could not be visualised through gel electrophoresis due to their small size, however AFM analysis provides evidence of this interaction by the moderate condensation effects of the complex.

### Probing the DNA cleavage mechanism with radical scavengers and BER enzymes

**Figure 7.**
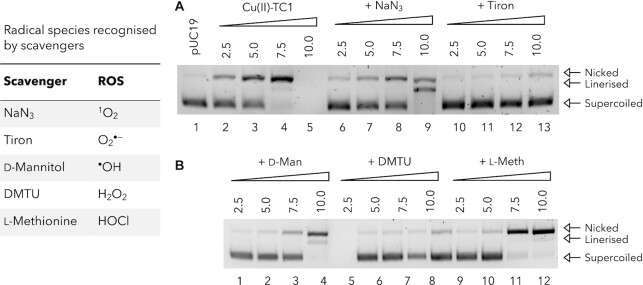
Probing the DNA damage mechanism of Cu(II)–TC1 using ROS scavengers. DNA cleavage reactions in the presence of (**A**), NaN_3_ and tiron (**B**), d-mannitol, DMTU and L-methionine.

The Cu(II)–TC1 DNA damage mechanism was probed *in vitro*, where the role of radical species were analyzed with ROS-specific scavengers and spin-trapping agents: (19,22) NaN_3_, tiron, d-mannitol, *N*,*N*′-dimethylthiourea (DMTU) and L-methionine. These antioxidants can sequester singlet oxygen (^1^O_2_), superoxide (O_2_^•−^), the hydroxyl radical (^•^OH), hydrogen peroxide (H_2_O_2_) and hypochlorous acid (HOCl) or a combination thereof (see inset table Figure 7). In these experiments, the delayed onset of SSB formation and the inhibition of DSBs were evident (Figure 7). H_2_O_2_ was identified as the most prevalent radical species required in the cleavage mechanism as DMTU (*N*,*N*'-dimethylthiourea) inhibited the complete transformation of supercoiled DNA to nicked open circular DNA. The ^•^OH scavenging agent d-mannitol had almost no influence on DNA cleavage, but l-methionine inhibited the formation of DSBs which may be attributed to scavenging of H_2_O_2_. Finally, the superoxide radical also appears to be important in the DNA cleavage mechanism since the introduction of tiron prevented DSB formation and delayed the onset of SSBs. Taken together, these results suggest the DNA damaging mechanism by Cu(II)–TC1 does not follow classic Fenton chemistry (which is dependent on diffusible ^•^OH radical generation), but instead follows a superoxide dismutase (SOD) type mechanism, whereby electron transfer from a reduced Cu(I) centre promotes the formation of O_2_^•−^ (or a metal-superoxo species), which is then converted to H_2_O_2_ by a second Cu(I) metal centre. Therefore, it might be the case that O_2_^•−^ does not promote DNA damage directly, but that tiron simply impedes the downstream formation of the peroxide (or metal-peroxo Cu(II)–OOH type) cleavage species.

**Figure 8.**
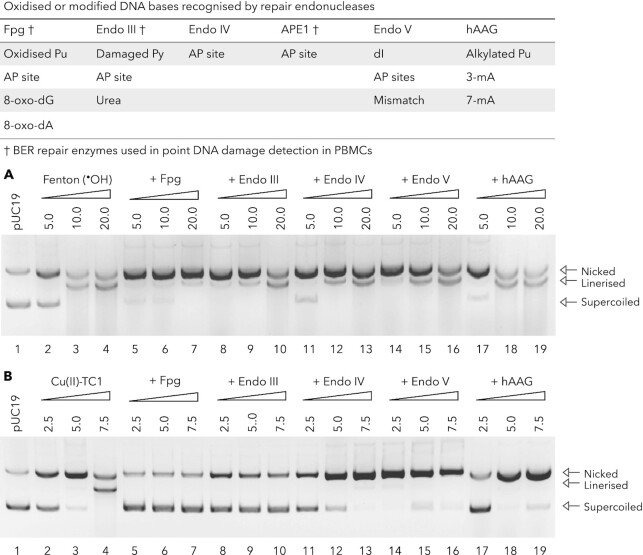
Probings DNA damage mechanism through repair endonuclease to identify damage lesions. (**A**) Fenton reagents, Cu(II) + H_2_O_2_ produce ^•^OH and (**B**) Cu(II)–TC1. pUC19 DNA was preincubated with Cu(II)–TC1 or Fenton reagents for 30 min at 37°C prior to repair enzyme addition and further incubated for 30 min. Enzymes were denatured for 20 min (50°C) and subjected to electrophoresis.

In order to examine the oxidative damage mechanism in greater detail, base excision repair (BER) enzymes, which can recognise specific lesion-specific modifications associated with oxidative damage were employed. This method was adapted from Fantoni *et al.* (19) with slight modifications. Repair proteins that recognise base-specific DNA lesions—or a small a small set of lesions with a common structural motif—are known as glycosylases and carefully remove their cognate damaged bases to yield abasic sites. Specific repair endonucleases can then recognise these AP (apurine/apyrimidine) sites and mediate strand nicking adjacent to the base-free lesion to create a single nucleotide gap that is filled by the insertion of a new base. The BER and endonuclease enzymes employed in this study are listed in Figure 8 (table inset) and initial experiments involved treating pUC19 with each enzyme to ensure that the plasmid was free of existing lesions (Supplementary Figure S7). Experiments were then designed to initiate DNA cleavage using a control hydroxyl radical (^•^OH) generated from a Fenton Cu^2+^/H_2_O_2_ reaction alongside the Cu(II)–TC1 complex. After 30 min of continuous exposure to the complex (used to initiate DNA damage), specific BER enzymes were introduced and the reactions were allowed to incubate for a further 30 min with results compared to control experiments lacking repair enzymes. In the copper-mediated Fenton reaction, DNA damage was inhibited by Fpg and Endo III, which indicates strand breaks are mediated by oxidised purine and pyrimidine bases generated by the hydroxyl radical (Figure 8A). Reaction conditions for the Cu(II)–TC1 (2.5–7.5 μM) were optimised to initiate SSB and DSB cleavage of supercoiled pUC19 (Figure 8B, lanes 2–4). In all cases, the presence of specific BER enzymes inhibited DSB formation since no linearised DNA was detected. In the presence of Fpg and Endo III, a significant reduction in SSB formation occurred thereby providing protection of intact plasmid DNA. In conjunction with the inhibition profile of Endo IV (and to a lesser extend Endo V), these results suggest the Cu(II)–TC1 complex can oxidise both purine and pyrimidine bases and promote the formation of abasic sites.

### APE1 selectively repairs Cu(II)–TC1 mediated single-strand breaks in PBMCs

**Figure 9.**
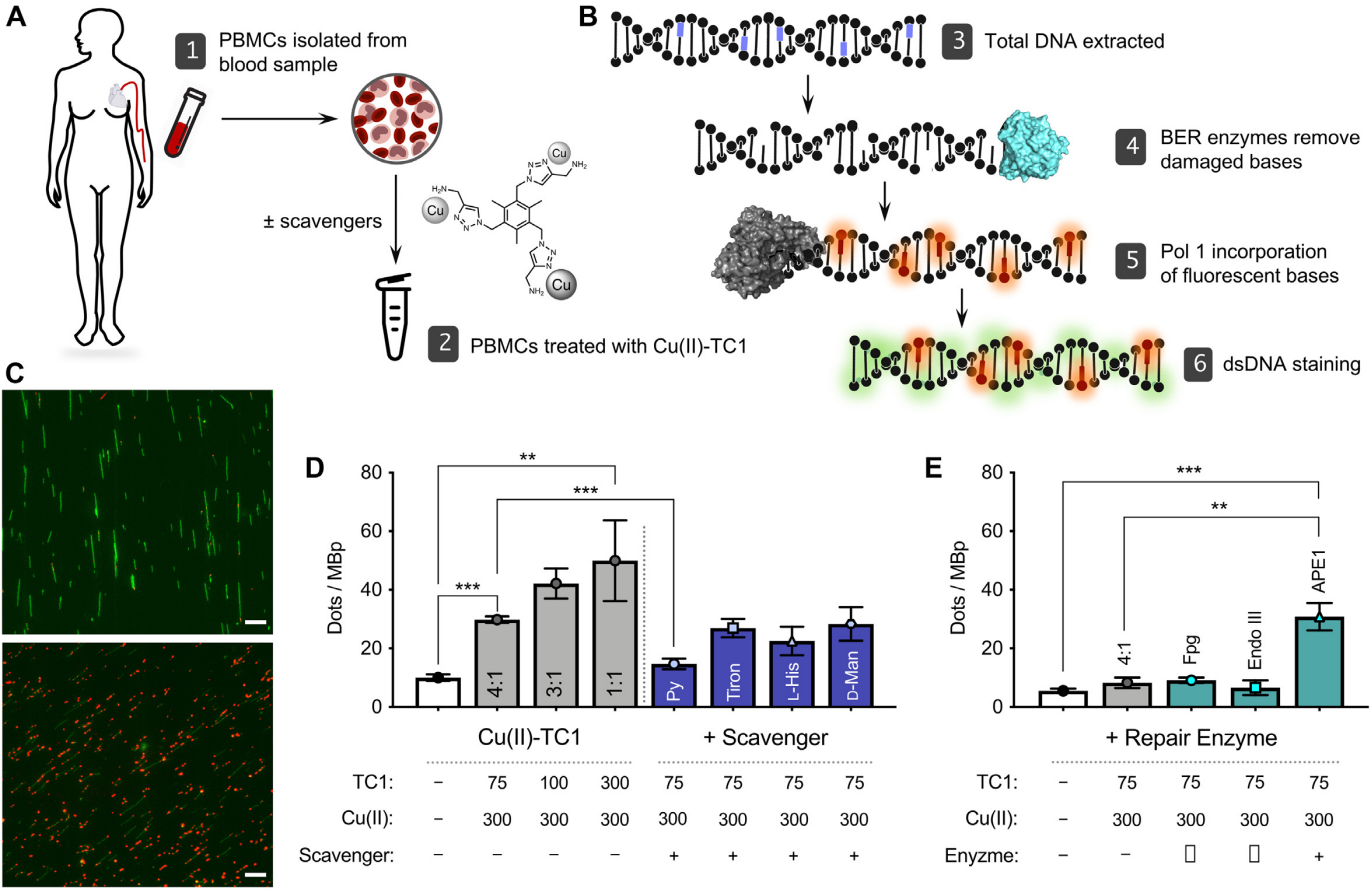
DNA damaging potential and effect of antioxidants in isolated PBMCs. (**A**) Illustration of sample collection and treatment: 1. PBMCs isolated from blood samples taken from healthy volunteers; 2. isolated cells treated with Cu(II)–TC1 in the presence or absence of ROS scavengers (pyruvate, tiron, L-histidine, D-mannitol). (**B**) Schematic of DNA labelling process: 3. DNA with damaged bases (purple) was extracted from PBMCs; 4. BER enzymes (Fpg, Endo III or APE1, cyan) remove oxidized bases; 5. fluorescent dNTPs (aminoallyl-dUTP-ATTO-647N, red) were incorporated by DNA polymerase 1 (grey); 6. dsDNA was labelled with YOYO-1 (green). (**C**) Representative microscopic images of labelled DNA from non-treated PBMCs (top) and Cu(II)–TC1 treated cells (bottom, Cu(II):TC1 ratio 1:1, 300:300 μM). Scale bar = 10 μm. Extended panel shown in Supplementary Figure S8. (**D**) DNA damage at varying TC1 concentrations with fixed Cu(II) concentration with and without radical scavengers (Cu(II):TC1 ratio 4:1, 300:75 μM). (**E**) Identification of specific DNA damage lesions with individual BER enzymes (Cu(II):TC1 ratio 4:1, 300:75 μM). Enzyme and scavenger controls are shown in Supplementary Figure S9.

Intracellular DNA damage mediated by Cu(II)–TC1 was examined using peripheral blood mononuclear cells (PBMCs). PBMCs were selected for this study as they are primary, non-immortalised cells and therefore provide a suitable representative model for accurate DNA repair mechanisms. An assay was designed (Figure 9A and B) whereby DNA extracted from metal complex-treated cells was initially exposed to specific BER enzymes and in a second step, DNA polymerase 1 was introduced with a fluorescent dNTP (aminoallyl-dUTP-ATTO-647N). Later, a dye (YOYO-1) was used to stain the DNA backbone and individual DNA molecules were stretched on silanized glass coverslips, where image analysis determined the extent of DNA damage by quantifying the incorporation of repaired bases on the single DNA molecule level (*cf*. Figure 9C) (51,63–65). PBMCs were exposed to Cu(II)–TC1 and intracellular SSBs were quantified (Figure 9d). In these experiments, the concentration of Cu(II) was kept constant (300 μM) while the TC1 concentration was varied to examine TC1:Cu(II) ratios from 1:1 to 1:4. Compared to untreated control cells, SSB formation increased ∼5.0-fold as the concentration of Cu(II)–TC1 increased to 300 μM. Next, PBMCs were incubated prophylactically with specific antioxidants—sodium pyruvate (H_2_O_2_), tiron (O_2_^•−^), l-histidine (^1^O_2_) or d-mannitol (^•^OH)—prior to the introduction of the Cu(II)–TC1 complex (Figure 9d). With sodium pyruvate, the decrease in Cu(II)–TC1-mediated SSBs was highest indicating that H_2_O_2_ is required in the DNA cleavage mechanism, in agreement with the *in vitro* experiments. Overall, the inhibition of SSB formation follows the trend: sodium pyruvate >> l-histidine > tiron > d-mannitol, indicating again that superoxide is not primary to SSB formation. Next, three different BER enzymes, Fpg, Endo III, and APE1 (derived from an AP endonuclease family similar to Endo IV) (66) were employed to quantify the selective repair of SSBs. In PBMCs treated with Cu(II)–TC1, a SSB fold increase of ∼1.1 (FpG), ∼0.8 (Endo III) and ∼3.7 (APE1) was detected, when compared to only DNA polymerase 1 treated Cu(II)–TC1 samples (Figure 9E). That APE1 was the most efficient enzyme in repairing the SSBs indicates that the majority of lesions generated by this complex are apurinic/apyrimidinic sites. In summary, these results strongly overlap with *in vitro* analysis and demonstrate abasic sites generated by a TC1-Cu-peroxide mediated mechanism leading to the formation of intracellular SSBs.

## CONCLUSION

The discovery of new metallodrugs with alternative modes of action is important not only to overcome resistance factors, but also in treating recalcitrant cancers where no (or severely limited) treatment options remain. In this work, we identified a click chemistry-based strategy to produce bioactive polynuclear complexes. A series of alkyl and aromatic amines with alkyne groups were developed and clicked to a 1,3,5-triazide mesitylene core to produce a library of polyamine ligands we called the Tri-Click (TC) series. Although several examples of DNA reactive polynuclear copper(II) complexes have been reported (67–69)—including di- and tri-nuclear species containing a central aryl core(25,60)—click chemistry has not been employed to furnish any of these existing libraries. Indeed, with the exception of G-quadruplex stabilising agents, (34) click chemistry has not been widely employed to design small molecule metallodrugs with nucleic acid recognition properties. From the isolated library, the simplest compound (TC1) was identified as a copper(II) binding ligand with potent chemical nuclease activity. TC1 was produced by reacting a primary amine, propargylamine, with mesitylene triazide and it appears this specific combination is required for copper-mediated nuclease activity. Significantly, when secondary and tertiary amine tripodal derivatives (TC2 and TC3) were investigated no DNA damage was observed. Aryl amines were also found to be inactive (including the primary amine ethynyl-aniline) suggesting that a conserved arrangement between the primary amine and the triazole group is required for copper-mediated DNA cleavage. Similar to classical chelators that mediate DNA damage in the presence of copper ions, (17) TC1 contains N,N donors that are capable of forming a 5-membered ring with copper (one from the primary amine and a second from the 3′  imine of the triazole ring). Coordination of copper is essential for DNA oxidation since protonation of the am(m)ine group in acidic buffer (pH 4.0) provides no associated cleavage activity. *In situ* ESI-MS analysis of Cu(II):TC1 solutions (1:1 to 8:1) identified the presence of three mono-cationic species corresponding to mono-, di-, and tri-nuclear forms of the complex: [Cu(TC1)(NO_3_)]^+^; [Cu_2_(TC1)(NO_3_)_3_]^+^; and [Cu_3_(TC1)(NO_3_)_5_]^+^, respectively. Correlating ESI-MS results with the biological activity of each Cu(II):TC1 ratio revealed the 3:1 complex as the active agent which produces a potent combination of DNA binding and cleaving activity.

The binding affinity of TC1 to duplex DNA is moderate (10^5^ – 10^6^ M^–1^) but in the presence of titrated copper(II) activity increases to 10^7^ M^–1^ for calf thymus and AT and GC co-polymers. This affinity places it in line with classical DNA recognition agents such as netropsin, dactinomycin and several state-of-art intercalating metallodrugs (18) but it appears Cu(II)–TC1 does not interact purely by groove binding or intercalation. Subsequent AFM analysis of Cu(II)–TC1 with plasmid DNA in the absence of reductant demonstrated the emergence of compact clusters surrounded by small portions of fragmented DNA. Based on this evidence it appears Cu-TC1 interacts electrostatically with DNA and initiates condensation that involves aggregation of sheared nucleic acid fragments that arise from SSBs. In a broadly similar vein, phosphate clamping polynuclear platinum complexes such as TriplatinNC which contain three *trans*-symmetric Pt(II) ions—and which also carry a net +6 cationic charge—demonstrate high DNA binding affinities and are potent condensation agents of DNA duplexes and nucleic acids in general (70–72). Polynuclear copper complexes are known to interact electrostatically with the phosphate group with a recent example [Cu_2_(*tetra*-(2-pyridyl)-NMe-naphthalene)]^2+^ (Cu_2_TPNap) demonstrating combined major groove residency and metal ion-mediated phosphate binding (23).

Cu(II)–TC1 demonstrates ‘self-activating’ DNA cleavage and is capable of mediating single strand breaks in the absence of an external (exogenous) reducing agent. A limited number of other copper(II) complexes—including tambjamine E and prodigiosin polypyrrole-based marine alkaloids, (57,58) macrocyclic colibactin, (12) and polynuclear agents including Cu_2_TPNap (23) and Cu-Oda ([{Cu(Phen)_2_}_2_(μ-Oda)]^2+^) (73)—can also damage DNA under these conditions with several demonstrating potent anticancer and antimicrobial properties (Supplementary Figure S10). These complexes are hypothesised to produce Cu(I) via autocatalysis involving one electron transfer from either a neighbouring metal ion (in polynuclear systems) or directly from coordinated polypyrrole ligands that oxidise to π-radical cations. In the presence of added ascorbate, the Cu(I)–TC1 complex is generated and is a potent DNA oxidant inducing SSBs and DSBs (arising from proximate SSBs or clustered damage) at low micromolar concentrations. This enhanced activity was corroborated by AFM imaging in the presence of ascorbate where SSBs and DSBs were identified across a 0.5–10 μM concentration gradient. DNA damage assays, performed both *in vitro* and in peripheral blood mononuclear cells (PBMCs), were then applied to probe the mechanism of copper-mediated DNA damage. Firstly, hydrogen peroxide appears crucial for strand breakages as sequestering this *in vitro* with DMTU and intracellularly with sodium pyruvate impedes DNA damage. The superoxide anion was also implicated since pUC19 DNA was protected in the presence of tiron, however, negligible protection was afforded to PBMCs pre-treated with tiron indicating a mechanism independent of diffusible superoxide. *In vitro* experiments with BER enzymes revealed the primary lesions generated are oxidised purine and pyrimidine bases recognised by Fpg and Endo III and, to a lesser extent, abasic sites that were recognised by Endo IV. This activity was then compared to intracellular results from PBMCs exposed to Cu(II)–TC1 where BER enzymes, together with fluorescently modified dNTPs, identified abasic sites—specifically recognised by APE1—as the major cellular lesion produced by this complex. Finally, the nuclease activity produced by Cu(II)–TC1 appears quite different to that of established polyamines (e.g. spermine, putrescine), which are known to modulate DNA damage and repair at abasic sites (74–77).

Several conclusions can be drawn by comparing *in vitro* quenching data and results from intracellular trapping experiments (where available) to other ‘self-activating’ copper complexes. Firstly, polypyrrole-based marine alkaloids prodigiosin and tambjamine E along with macrocyclic colibactin all cleave plasmid DNA *in vitro* without exogenous reductant using a peroxide-dependant mechanism inhibited by the catalase enzyme (12,57,58). All three natural products maintain their *in vitro* activity in the presence of superoxide dismutase (SOD) and hydroxyl radical scavengers (D-mannitol or *tert*-butyl alcohol) indicating a mechanism that is independent of diffusible superoxide and hydroxyl radicals. The major groove binder Cu_2_TPNap also displays self-activation and it appears diffusible free radicals are similarly not involved in the cleavage process since 8-oxo-dG is not generated (23). However, in contrast to naturally occurring systems, strand breaks were completely inhibited *in vitro* by sequestering superoxide with tiron and SSBs and DSBs attenuated in the presence of pyruvate. DNA cleavage by Cu-Oda also requires superoxide with *in vitro* and intracellular activity impeded by tiron (22). However, unlike Cu_2_TPNap and naturally occurring polypyrroles, DNA cleavage is not affected by hydrogen peroxide scavengers. Instead, the complex mediates intracellular singlet oxygen production in its doubly reduced form (i.e. where both copper ions are in the + 1 oxidation state) (73). Considering these classes of ‘self-activated’ systems, the DNA damaging activity of Cu(II)–TC1 appears closely related to the copper(II) complexes of polypyrrole-based alkaloids and macrocyclic colibactin since: (a) activity is dependent on hydrogen peroxide; (b) the superoxide radical is not involved during the cellular cleavage process and (c) singlet oxygen and Fenton-type products are not involved in the DNA damage mechanism.

In summary, we identified and characterised a new click chemistry method to produce novel polynuclear DNA damaging metallodrugs. Conclusive evidence was provided demonstrating that a lead agent from this series directly mediates oxidative DNA damage in the presence of copper(II) using a ‘self-activating’ mechanism. There is good agreement between the *in vitro* and cellular DNA damaging modes supporting a mechanism closely related to copper complexes of naturally occurring marine alkaloids along with macrocyclic colibactin. Therefore, our work shows by applying this particular synthetic strategy, it is possible to simulate the activity of state-of-art DNA damaging natural products. This discovery now provides a framework for future investigators to develop enhanced DNA damaging agents with unique chemotherapeutic properties that may circumvent innate cellular DNA repair machinery.

## Supplementary Material

gkab817_Supplemental_File
