# Supplementary material for: Click and Cut: a click chemistry approach to developing oxidative DNA damaging agents
Source: Nucleic Acids Res. 2021 Sep 27;49(18):10289–308. doi: 10.1093/nar/gkab817 (PMC8501983; doi:10.1093/nar/gkab817)
Supplement: gkab817_Supplemental_File [file gkab817_supplemental_file.pdf]

Click and Cut: A Click Chemistry Approach for Developing Therapeutic DNA Oxidants

Natasha McStay[1,3,†], Creina Slator[1,†], Vandana Singh[2], Alex Gibney[1,3], Fredrik Westerlund[2] and Andrew Kellett*[1,3]

[1] School of Chemical Sciences and National Institute for Cellular Biotechnology, Dublin City University, Glasnevin, Dublin 9, Ireland.
[2] Department of Biology and Biological Engineering, Chalmers University of Technology, Gothenburg, Sweden.
[3] Synthesis and Solid-State Pharmaceutical Centre, School of Chemical Sciences, Dublin City University, Glasnevin, Dublin 9, Ireland.

S-1. Detailed reaction schemes and Tri-Click controls

S-2. Electrophoresis gels and ESI-MS analysis

S-3. Point DNA damage detection

S-4. NMR characterisation

S-1. Detailed reaction schemes and Tri-Click controls

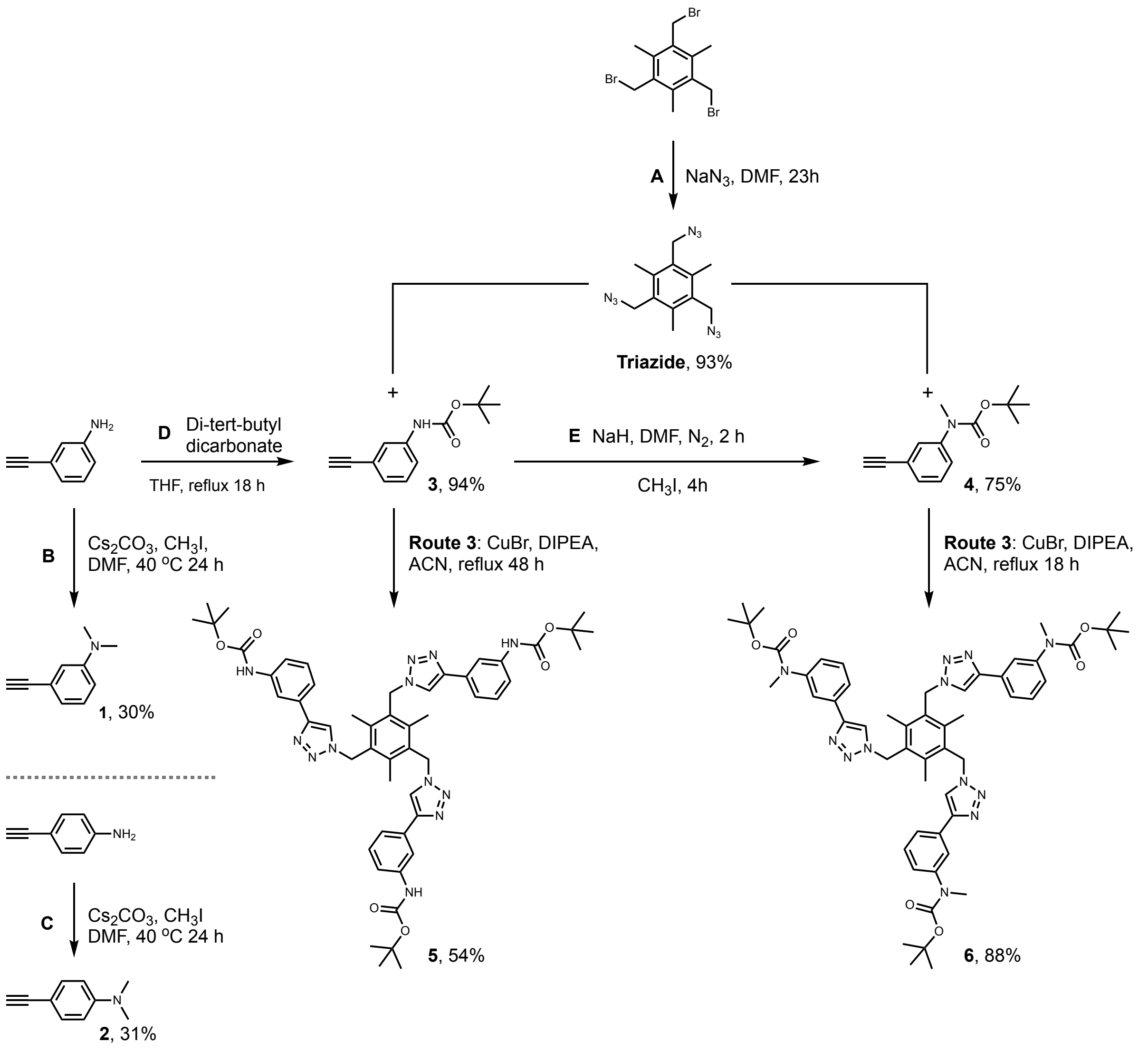

Figure S-1. Reaction routes to generate ligand intermediates and triazide scaffold. **A**, S_N2 substitution to give central triazide core. **B** and **C**, Aniline dialkylation to procedure 3-ethynyl-*N,N*-dimethylaniline (**1**) and 4-ethynyl-*N,N*-dimethylaniline (**2**). **D**, Protected boc-3-ethynylaniline (**3**) and **E**, methylated equivalent boc-3-ethynyl-*N*-methylaniline (**4**). Click reaction **route 3** to generate Tri-Click boc-3-ethynylaniline (**5**) and Tri-Click boc-3-ethynyl-*N*-methylaniline (**6**).

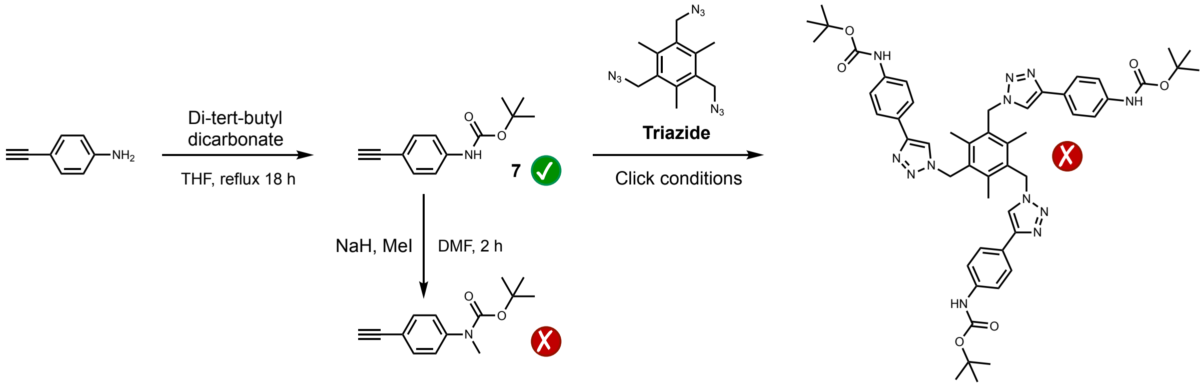

Figure S-2. Attempted reactions of 4-ethynylaniline series. Successful Boc-protection of 4-ethynylaniline (**7**), failed methylation of **7** and attempted ‘click’ reactions as detailed in Table S-1.

Table S-1. Reaction conditions attempted with boc-4-ethynylaniline.

Rxn	Conditions	Success
1	To a solution of triazide (1 mmol), DIPEA (1 mmol) and CuBr (1 mmol) in ACN (25 mL), boc-4-ethynylaniline (3.1 mmol) was added as a solution in ACN (5 mL). The reaction was carried out under N_2 at rt, 24 h.	X
2	To a solution of triazide (1 mmol), DIPEA (1 mmol) and CuBr (1 mmol) in ACN (25 mL), boc-4-ethynylaniline (3.1 mmol) was added as a solution in ACN (5 mL) over 10 min. The reaction was carried out under N_2 at 50°C, 24 h.	X
3	To a solution of triazide (1 mmol), DIPEA (1 mmol) and CuBr (1 mmol) in ACN (25 mL), boc-4-ethynylaniline (3.1 mmol) was added as a solution in ACN (5 mL) over 15 min. The reaction was carried out under N_2 at rt, 144 h.	X
4	To a solution of triazide (0.7 mmol), Na-L-ascorbate (20%), $CuSO_4$ (10%), THF/H_2O (1:1, 10 mL). boc-4-ethynylaniline (2.2 mmol) was added and reaction left to stir at rt, 16 h.	X
5	To a solution of triazide (1 mmol), DIPEA (1 mmol) and CuBr (1 mmol) in degassed ACN (25 mL), boc-4-ethynylaniline (3.1 mmol) was added as a solution in ACN (5 mL) over 10 min. The reaction was carried out under argon at 50°C, 72 h.	X
6	To a solution of triazide (1 mmol), DIPEA (1 mmol) and CuBr (1 mmol) in degassed ACN (15 mL), boc-4-ethynylaniline (3.1 mmol) was added as a solution in ACN (5 mL). The reaction was carried out under N_2 at reflux, 24 h.	X
7	To a solution of triazide (1 mmol) and boc-4-ethynylaniline (3.1 mmol) in THF/H_2O (1:1, 10 mL), in the presence of $CuSO_4$ (5%) and Na-L-ascorbate (10%) at 30°C, 48 h.	X
8	To a solution of triazide (0.7 mmol) and boc-4-ethynylaniline (2.17 mmol) in *t*-BuOH/H_2O (1:1, 10 mL), in the presence of $CuSO_4$ (1%) and Na-L-ascorbate (10%) at 50°C, 24 h.	X
9	To a solution of triazide (1 mmol), TEA (1 mmol) and CuI (1 mmol) in dry DMF (20 mL) a solution of boc-4-ethynylaniline was added slowly over 20 min in a solution of DMF (5 mL), reaction was carried out under N_2 at rt, 24 h.	X

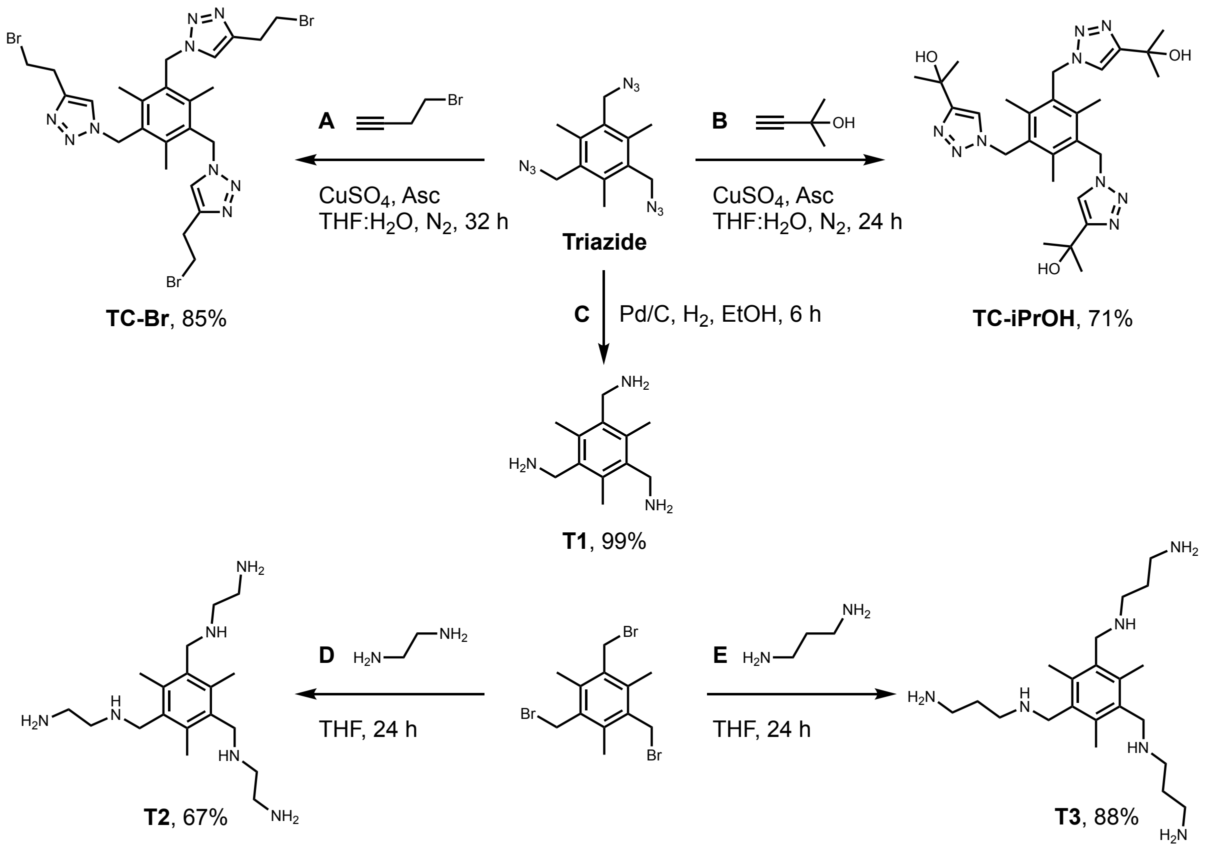

Figure S-3. Synthetic routes for Tri-Click SAR controls. **A** and **B**, Click reactions to generate TC-Br and TC-iPrOH. **C**, Azide reduction to form triamine, T1. **D** and **E**, Nucleophilic substitution to produce polyamines T2 and T3.

S-2. Gel electrophoresis.

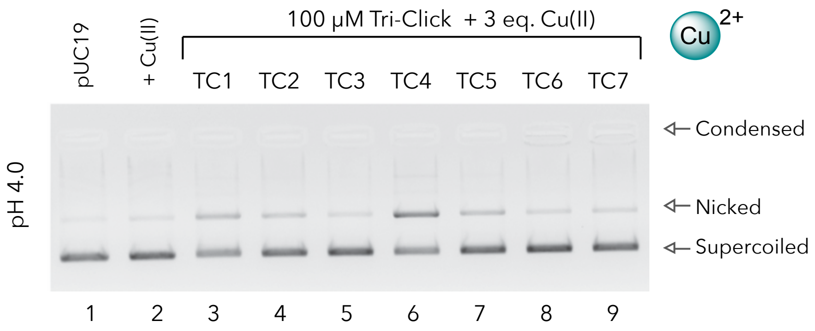

Figure S-4. Supercoiled (400 ng) pUC19 exposed to Tri-Click series (100 µM) and 3 equivalents of Cu(II) in acidic buffer (NaOAc, 80 mM, pH 4.0) over 24 h.

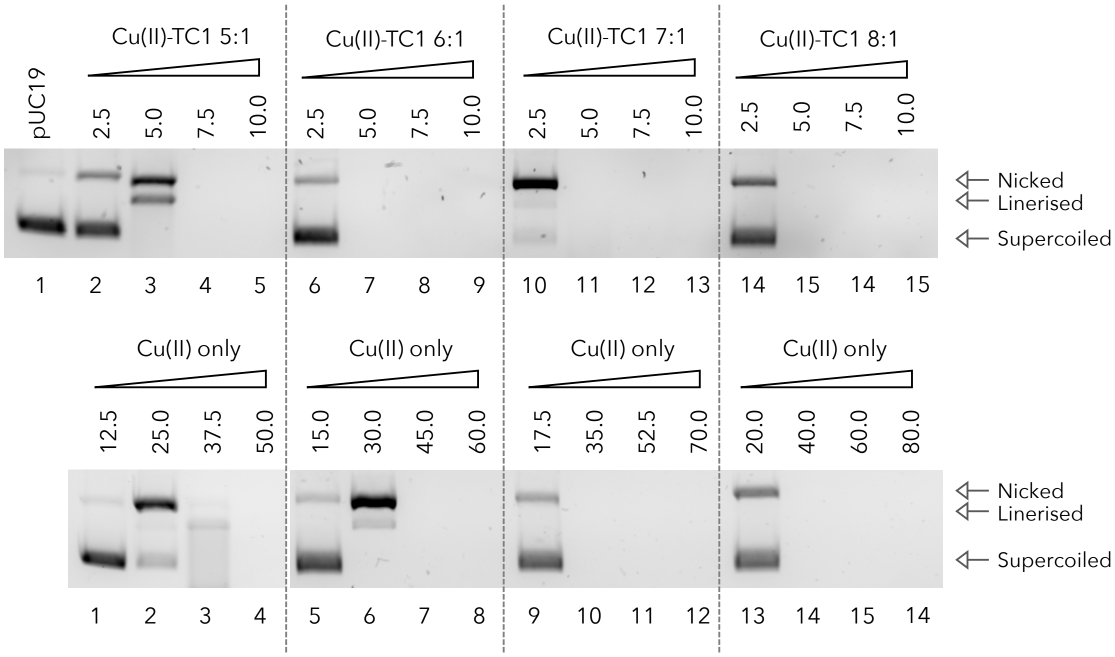

Figure S-5. Supercoiled (400 ng) pUC19 exposed to Cu(II)-TC1 at 5:1 – 8:1 Cu(II):TC1 ratios for 30 mins in the presence of reductant, Na-L-ascorbate (1 mM).

Table S-2. Accurate ESI-MS. Relative intensities corresponding to the formation of three polynuclear species; $[Cu(TC1)(NO_3)]^+$ $[Cu_2(TC1)(NO_3)_3]^+$; and $[Cu_3(TC1)(NO_3)_5]^+$. Solutions of Cu(II):TC1 were prepared *in situ* at 0:1 to 8:1 ratios. Relative intensities (accounting for isotopic abundance of ^{63}Cu and ^{65}Cu) were normalised to the sum of all species and represented as %.

$$\%I_x = \frac{I_x}{I_A + I_B + I_C} \text{ x } 100$$

	**Mononuclear species**			**Dinuclear species**				**Trinuclear species**					**All Species**	**Normalised Intensity (%I_X)**		
**Cu:TC1 ratio**	M+ (^{63}Cu)	M+2 (^{65}Cu)	**Total (I_A)**	M+ (^{63}Cu / ^{63}Cu)	M+2 (^{63}Cu / ^{65}Cu)	M+4 (^{65}Cu / ^{65}Cu)	**Total (I_B)**	M+ (^{63}Cu / ^{63}Cu / ^{63}Cu)	M+2 (^{63}Cu / ^{63}Cu / ^{65}Cu)	M+4 (^{63}Cu / ^{65}Cu / ^{65}Cu)	M+6 (^{65}Cu / ^{65}Cu / ^{65}Cu)	**Total (I_C)**	**Sum (I_A+I_B+I_C)**	**% Mono-**	**% Di-**	**%Tri-**
0:1	0	0	0	0	0	0	0	0	0	0	0	0	0	0.00	0.00	0.00
1:1	18.11	9.58	27.69	0	0	0	0	0	0	0	0	0	27.69	100.00	0.00	0.00
2:1	29.12	12.79	41.91	4.25	5.9	0.66	10.81	0.39	0.59	0.31	0.14	1.43	54.15	77.40	19.96	2.64
3:1	17.48	7.88	25.36	4.47	6.18	0.84	11.49	1.33	2.3	1.03	0.28	4.94	41.79	60.68	27.49	11.82
4:1	6.80	3.09	9.89	2.15	2.85	0.43	5.43	1.19	2.14	0.91	0.22	4.46	19.78	50.00	27.45	22.55
5:1	3.59	1.69	5.28	1.29	1.63	0.26	3.18	0.89	1.62	0.69	0.17	3.37	11.83	44.63	26.88	28.49
6:1	2.16	1.02	3.18	0.89	1.12	0.2	2.21	0.84	1.57	0.66	0.17	3.24	8.63	36.85	25.61	37.54
7:1	1.40	0.66	2.06	0.68	0.84	0.15	1.67	0.69	1.25	0.54	0.14	2.62	6.35	32.44	26.30	41.26
8:1	1.03	0.48	1.51	0.57	0.69	0.13	1.39	0.62	1.10	0.47	0.13	2.32	5.22	28.93	26.63	44.44

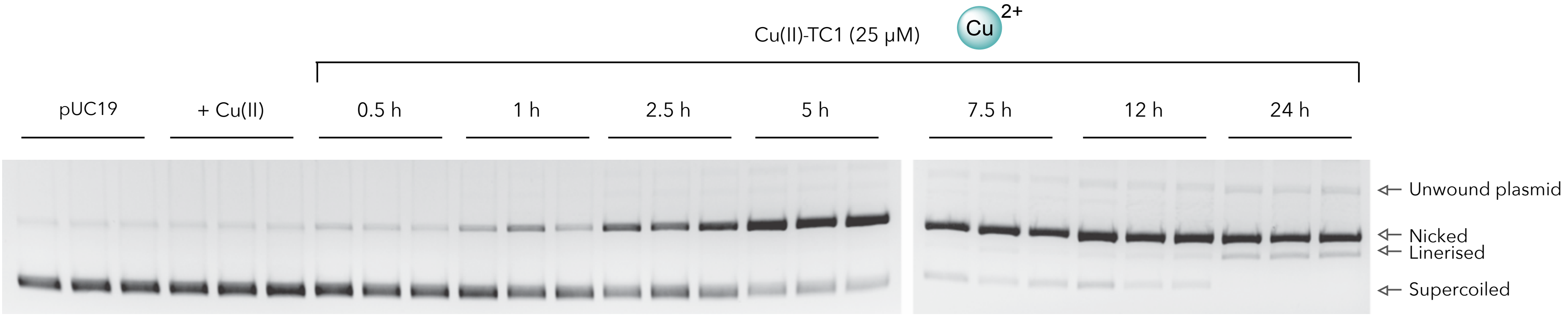

Figure S-6. Nuclease activity of Cu(II)-TC1 (Cu(II):TC1 75:25 μM) at various incubation times over 24 h. Band density was analysed and is shown in Figure 5D where n=3. Cu(II) only control was incubated for 24 h. All reactions were carried out in 80 mM HEPES in the absence of reductant.

A Oxidised or modified DNA bases recognised by repair endonucleases

BER enzyme	Selected recognised lesions	Cleavage site	DNA product	Enzymatic activity
**Fpg**	AP site (apurinic), oxidized purines, 8-oxo-dG/dA, FaPy-dG/dA	Glycosidic bond; phosphodiester bond 3′ + 5′ to AP site	AP site; 1nt gap	Glycosylase; AP lyase
**Endo III**	AP site (apyrimidinic), damaged pyrimidines, Urea	Glycosidic bond; 1st phosphodiester bond 3′ to AP site	1nt gap	Glycosylase; AP lyase
**Endo IV**	AP site	1st phosphodiester bond 5′ to AP site	1nt gap	Endonuclease
**APE1**	AP site	1st phosphodiester bond 5′ to AP site	1nt gap	Endonuclease
**Endo V**	dI, AP site, mismatch	2nd phosphodiester bond 3′ to dI	nick	Endonuclease
**hAAG**	Alkylated Pu, 3-mA, 7-mG	N-glycosidic bond	AP site	Glycosylase

B

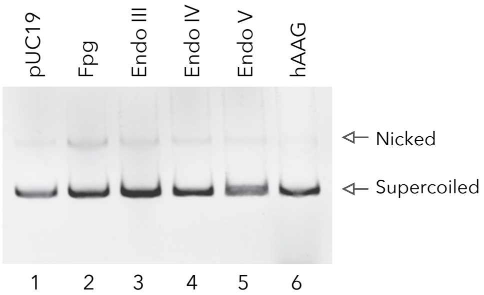

Figure S-7. A, Table highlighting base lesions recognised or excised by respective repair enzymes. Abbreviations are as follows:, AP site = apurininc/apyrimdininc site (also known as AB or abasic site), APE1 = apurinic /apyrimidinic endonuclease 1, dA = deoxyadenosine, dG = deoxyguanosine, dI = deoxyinosine, endo = endonuclease, FaPy = formamidopyrimidine, Fpg = formamidopyrimidine-DNA glycosylase, hAAG = human alkyladenine DNA glycosylase, m = methyl. **B**, pUC19 only controls in the presence of repair enzymes, Fpg, Endo III, Endo IV, Endo V, and hAAG in respective buffer and reaction conditions.

S-3. DNA damage detection in PBMCs

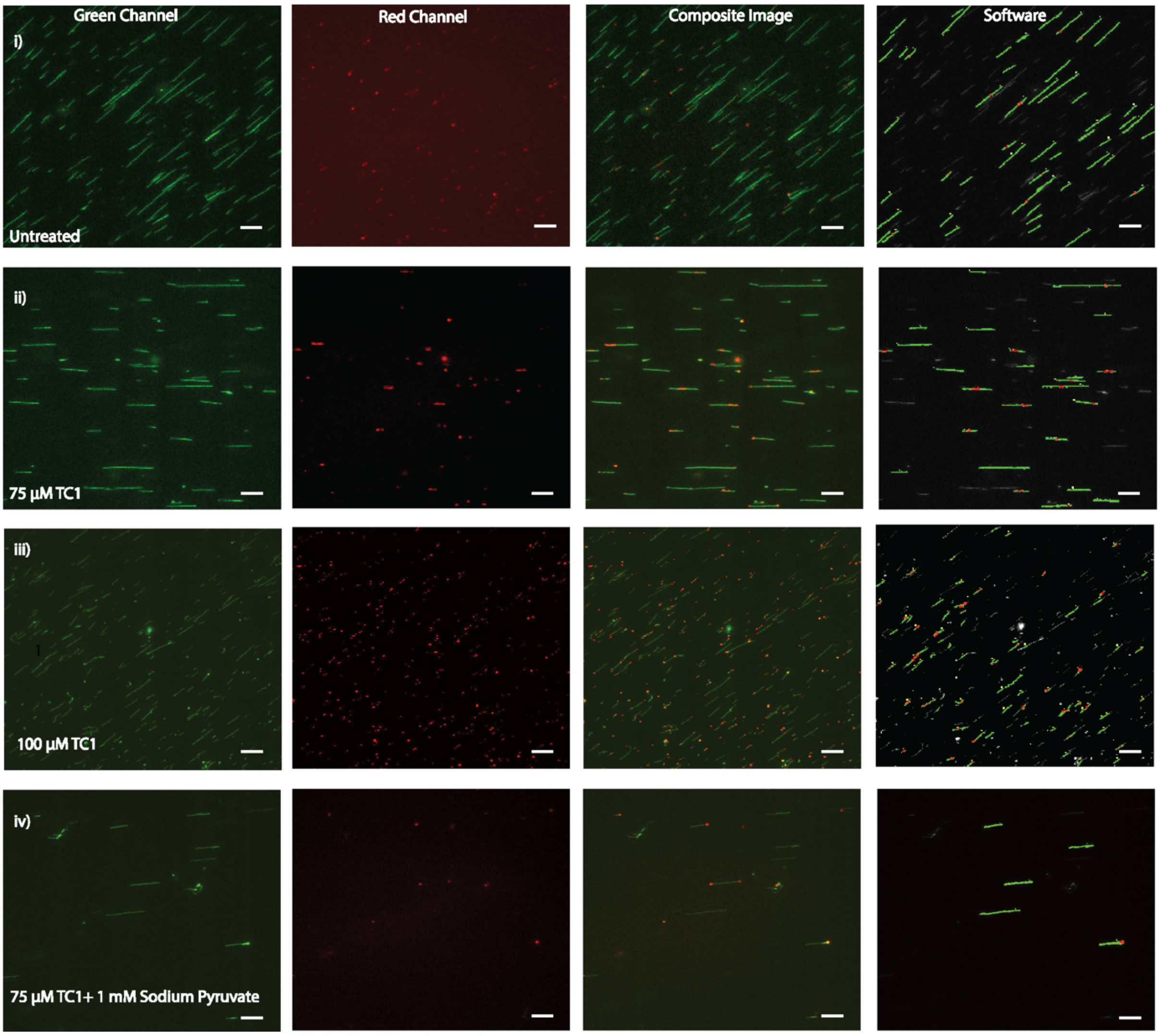

Figure S-8 Representative images for i) untreated, ii) Cu(II):TC1 ratio 300:75 μM (4:1), iii) Cu(II):TC1 ratio 300:100 μM (3:1), iv) Cu(II):TC1 ratio 300:75 μM (4:1) with sodium pyruvate. Scale bar = 10 μm.

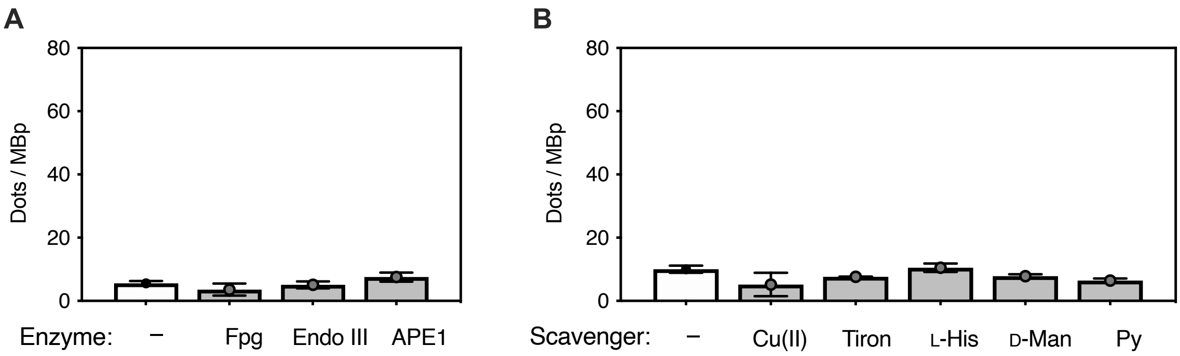

Figure S-9. **A**, Repair enzyme controls (Fpg, Endo III and APE1) and **B**, scavenger controls (tiron, L-histidine, D-mannitol and pyruvate) in PBMCs.

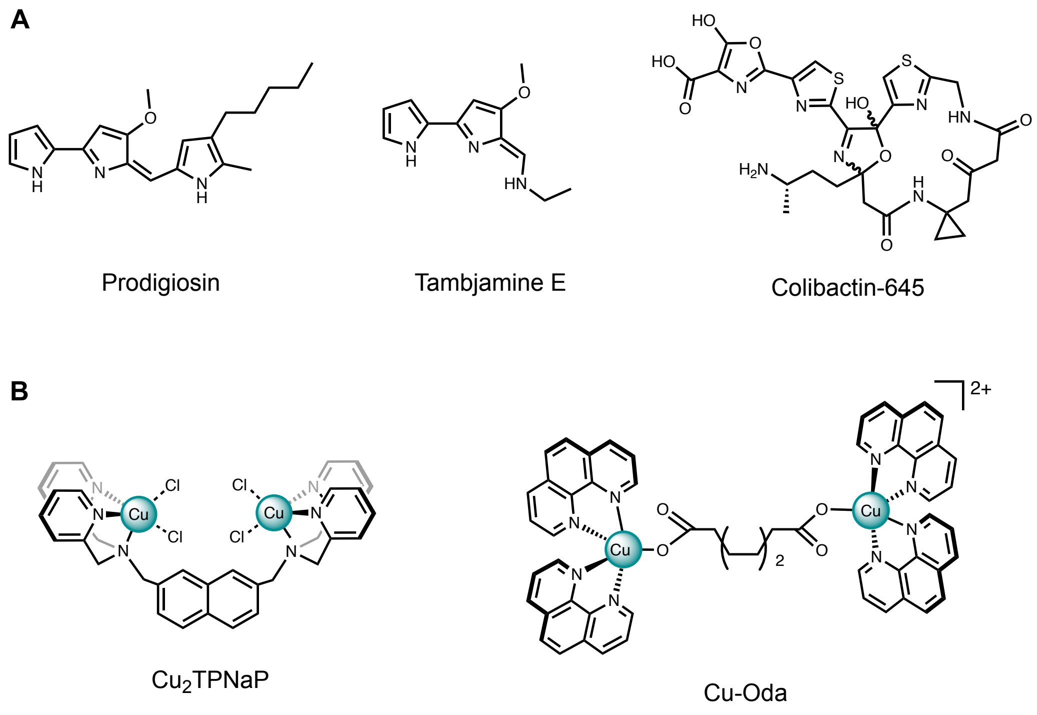

Figure S-10. **A**, Naturally occurring polypyrroles that chelate Cu(II); prodigiosin and tambjamine E marine alkaloids, and macrocyclic colibactin-645. **B**, Polynuclear complexes Cu_2TPNap and Cu-Oda.

S-4. NMR Spectroscopy

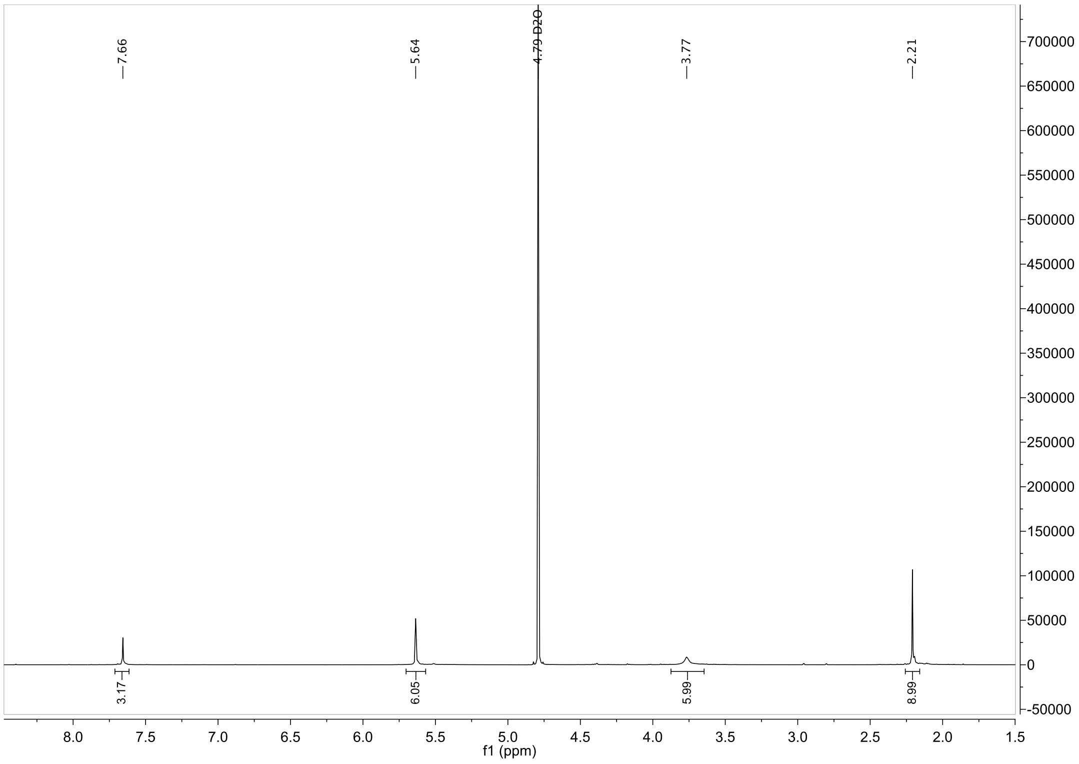

Figure S-11 ^{1}H NMR spectra for Tri-Click propargylamine (**TC1**).

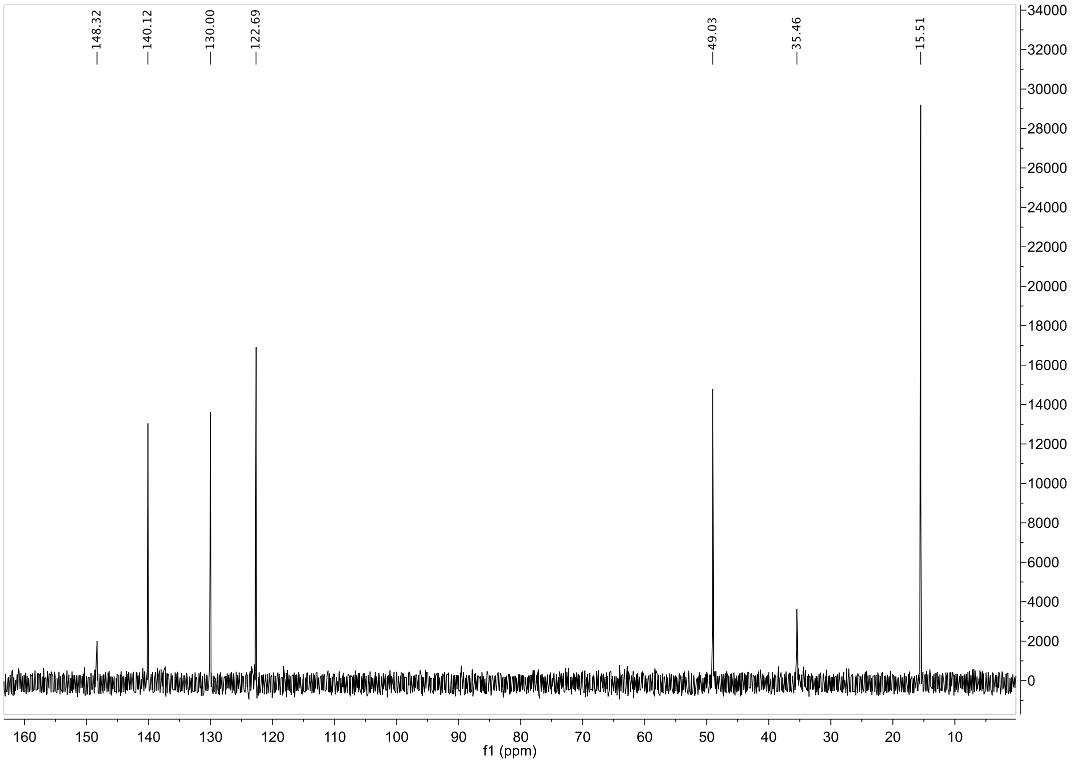

Figure S-12. ^{13}C NMR spectra for Tri-Click propargylamine (**TC1**).

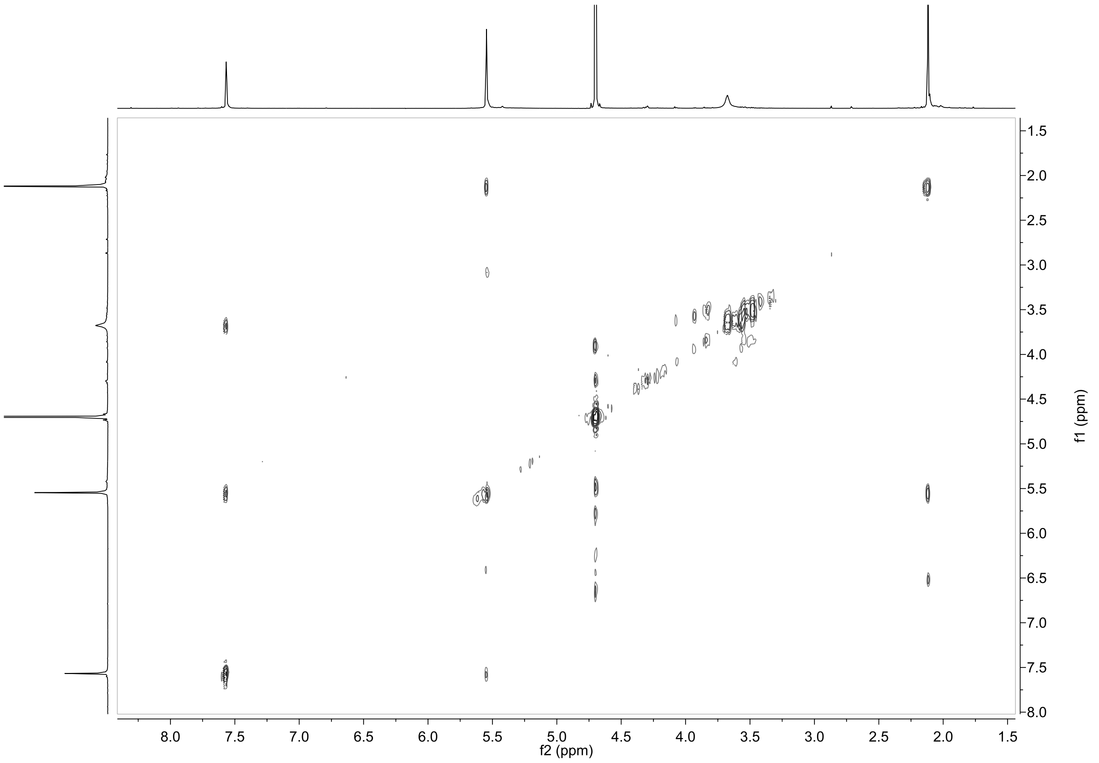

Figure S-13. COSY NMR spectra for Tri-Click propargylamine (**TC1**).

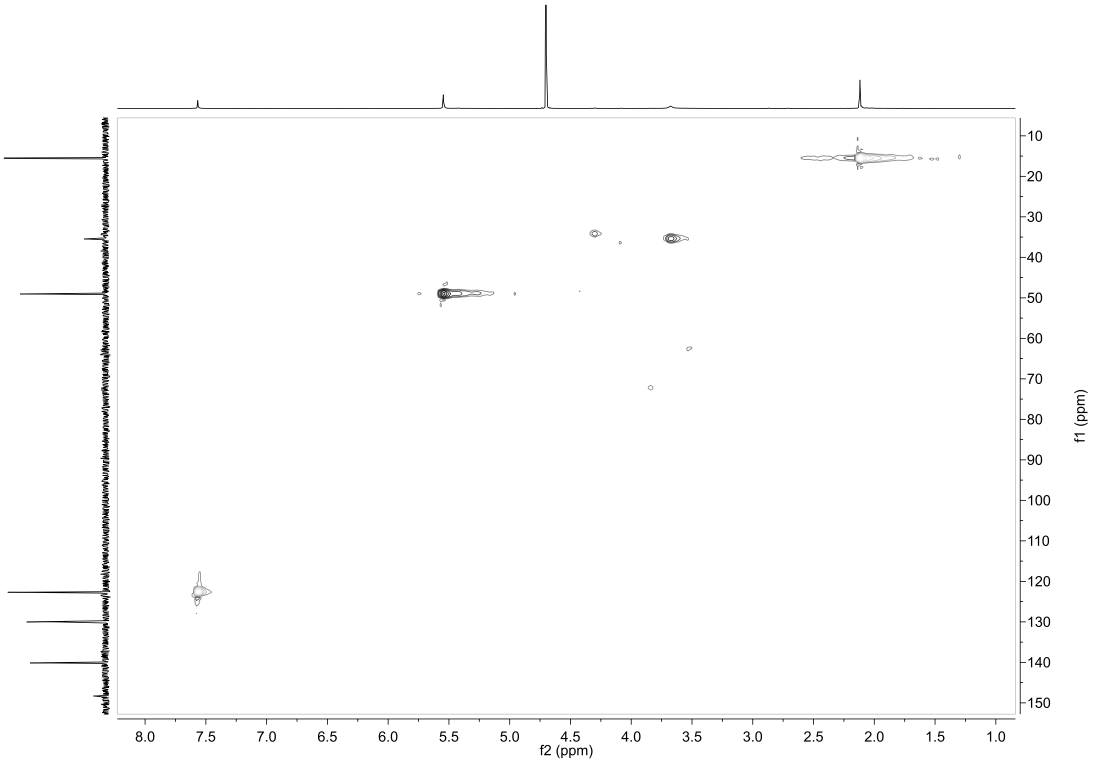

Figure S-14. HSQC NMR spectra for Tri-Click propargylamine (**TC1**).

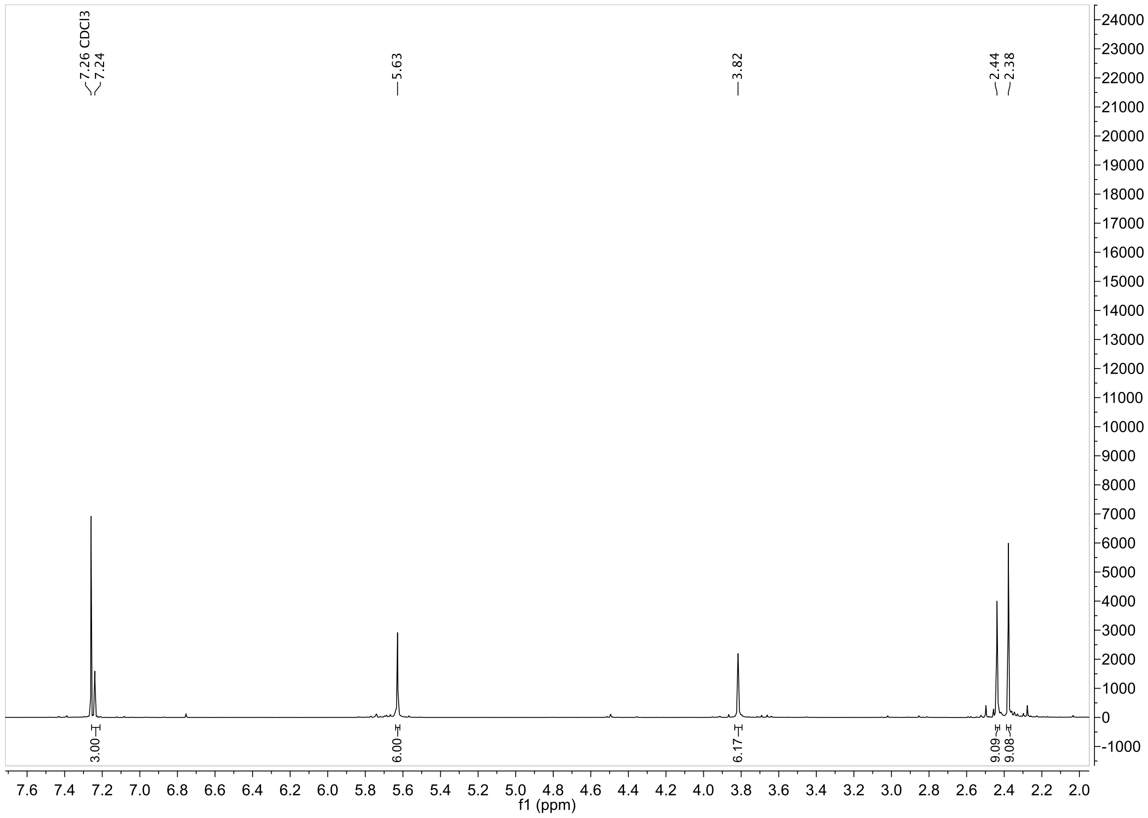

Figure S-15. ^{1}H NMR spectra for Tri-Click *N*-methyl propargylamine (**TC2**).

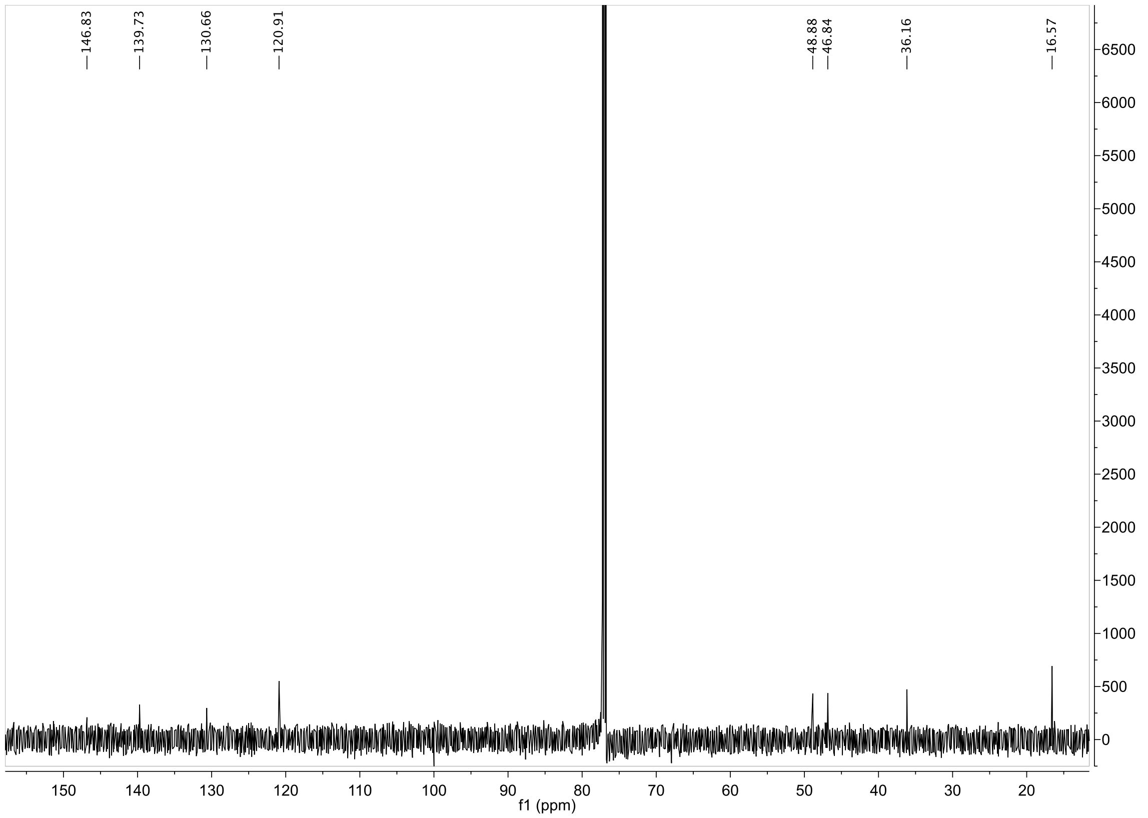

Figure S-16. ^{13}C NMR spectra for Tri-Click *N*-methyl propargylamine (**TC2**).

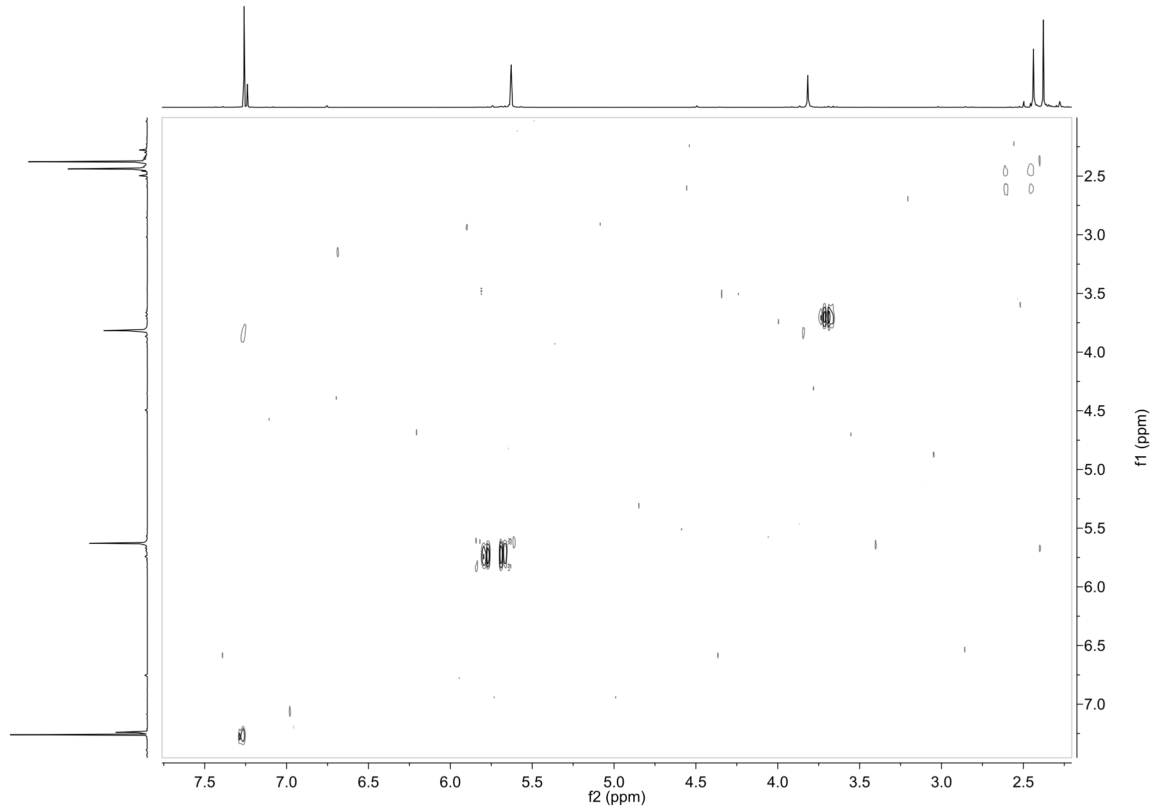

Figure S-17. COSY NMR spectra for Tri-Click *N*-methyl propargylamine (**TC2**).

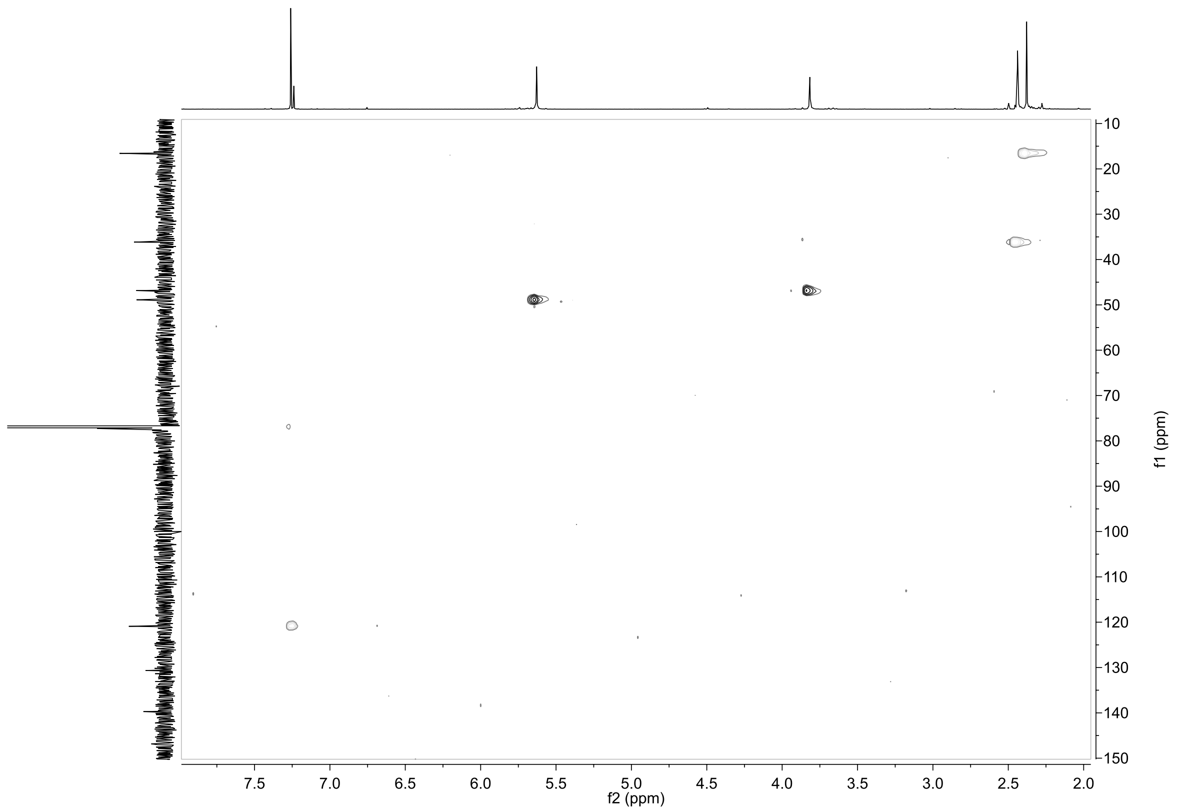

Figure S-18. HSQC NMR spectra for Tri-Click *N*-methyl propargylamine (**TC2**).

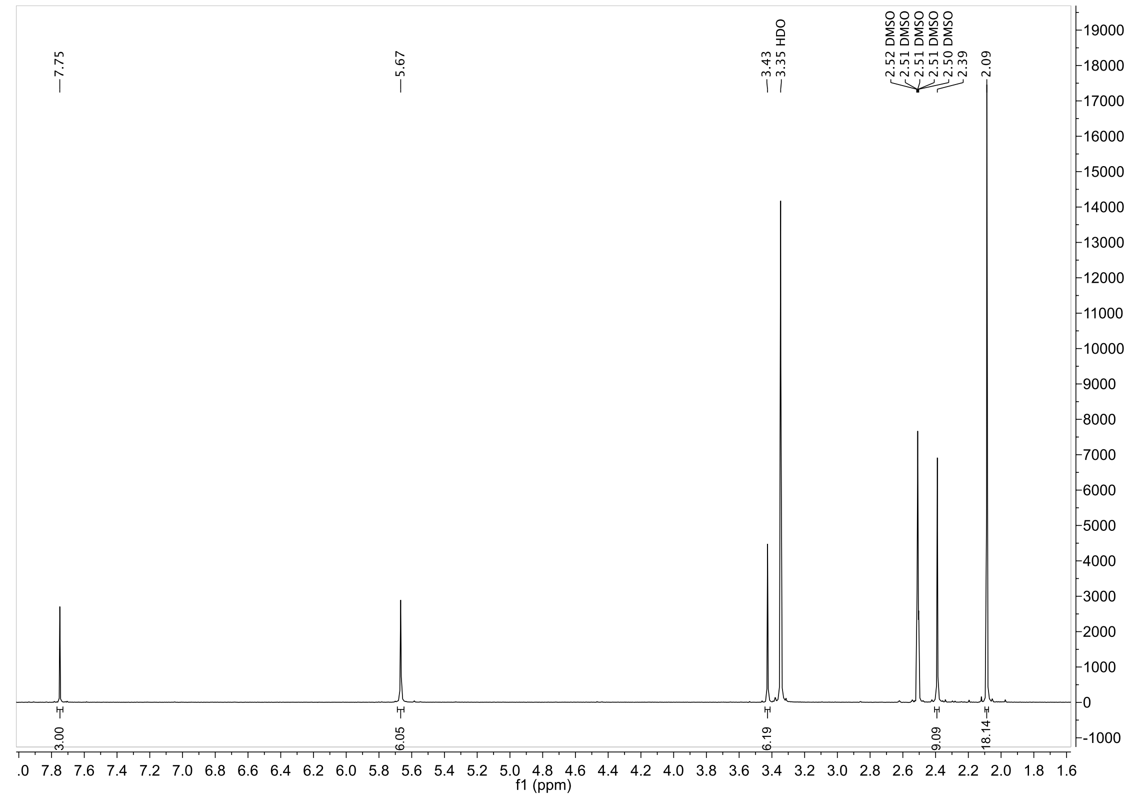

Figure S-19. ^{1}H NMR spectra for Tri-Click *N,N*-dimethylprop-2-yne-1-amine (**TC3**).

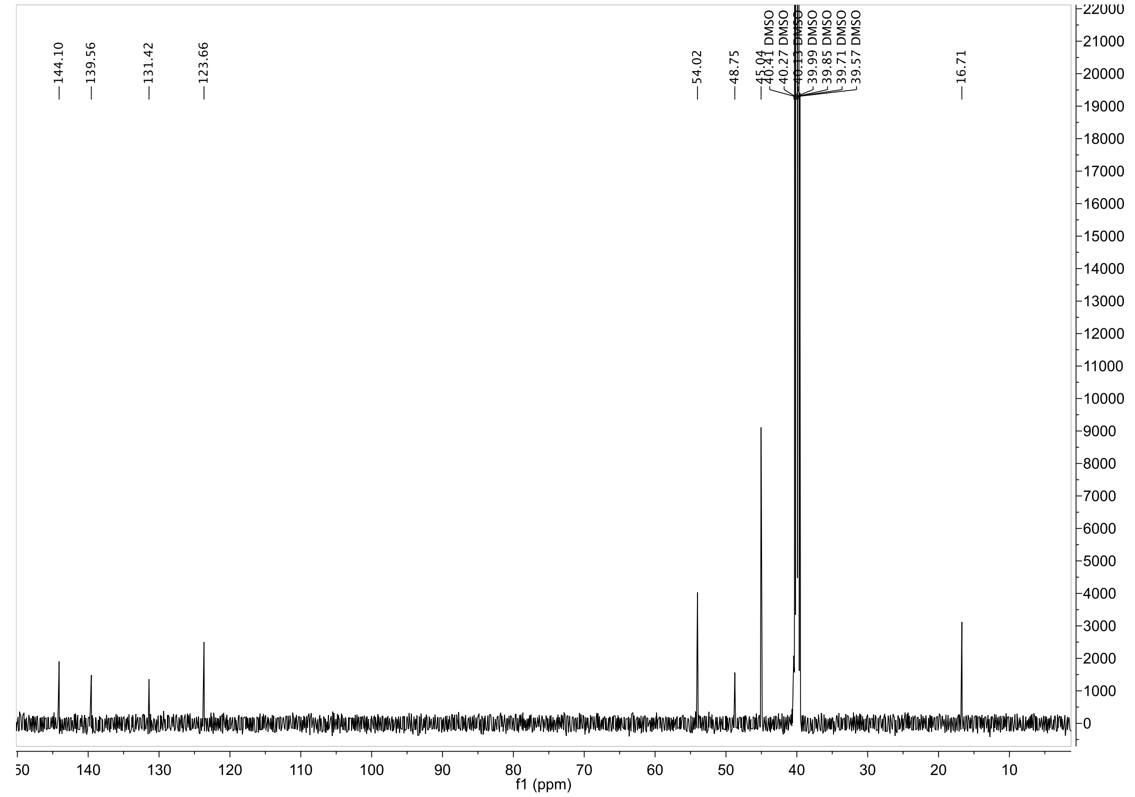

Figure S-20. ^{13}C NMR spectra for Tri-Click *N,N*-dimethylprop-2-yne-1-amine (**TC3**).

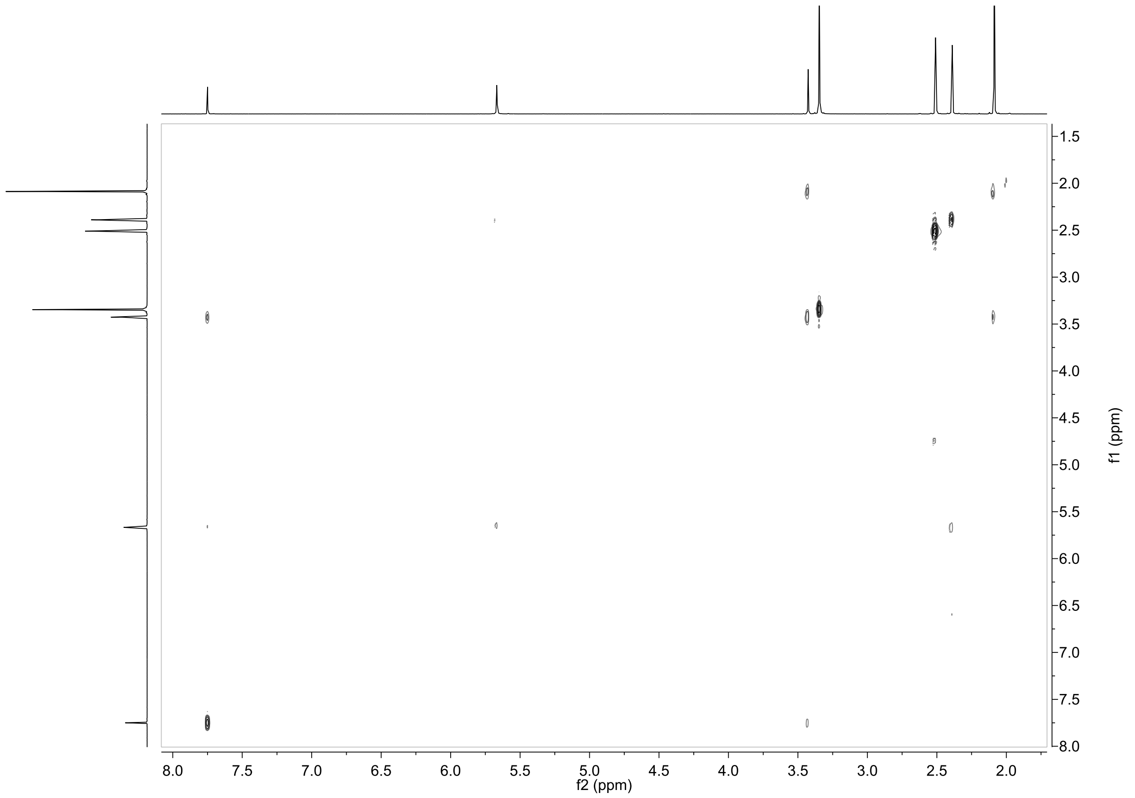

Figure S-21. COSY NMR spectra for Tri-Click *N,N*-dimethylprop-2-yne-1-amine (**TC3**).

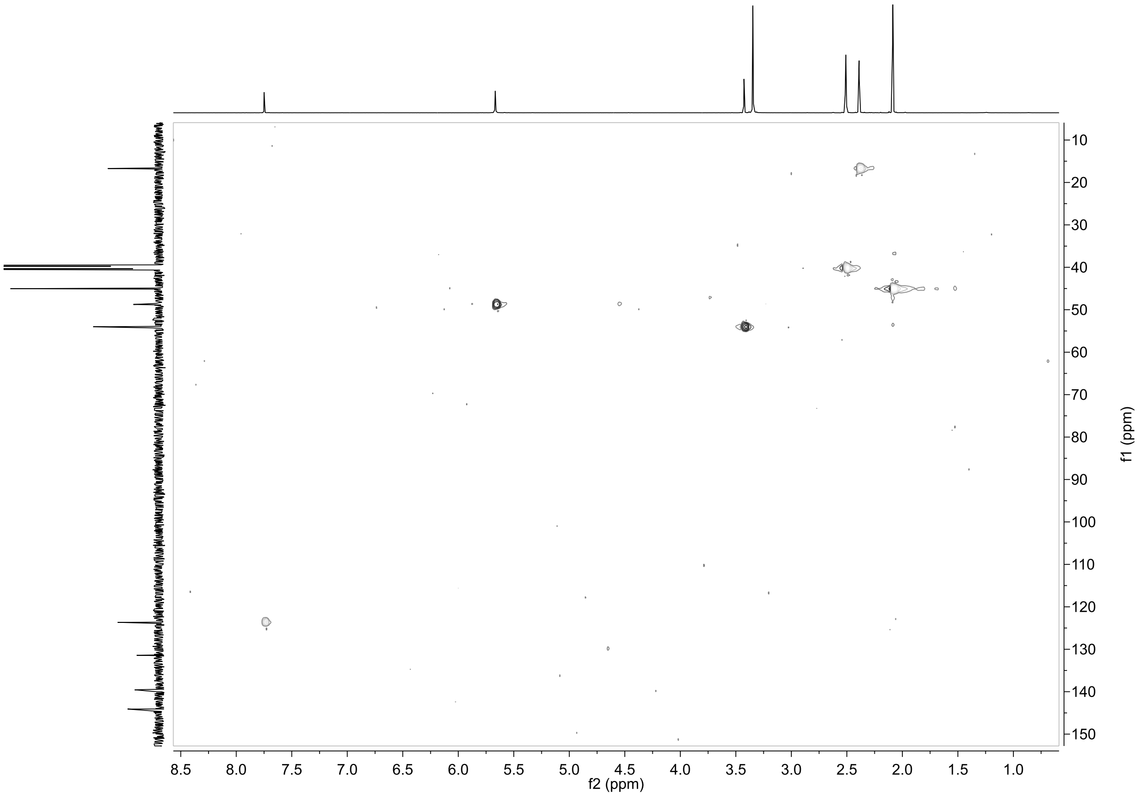

Figure S-22. HSQC NMR spectra for Tri-Click *N,N*-dimethylprop-2-yne-1-amine (**TC3**).

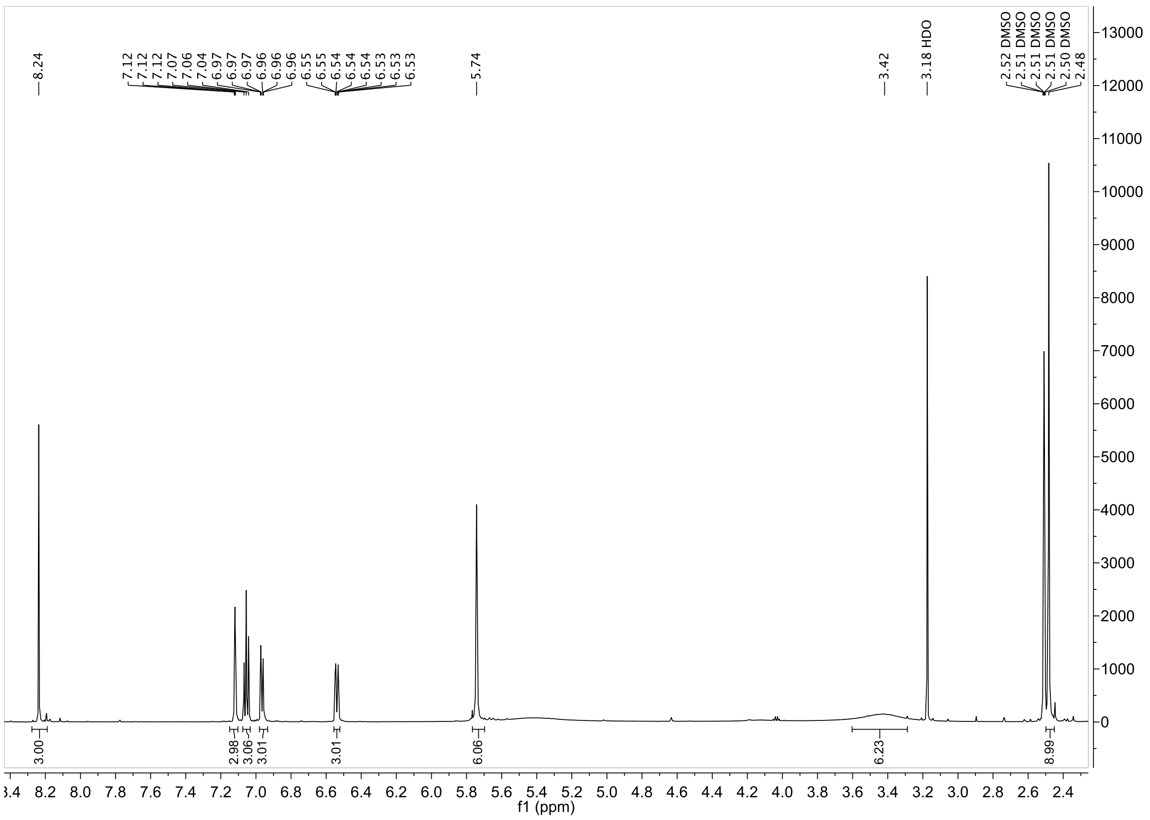

Figure S-23. ^{1}H NMR spectra for Tri-Click 3-ethynyl aniline (**TC4**).

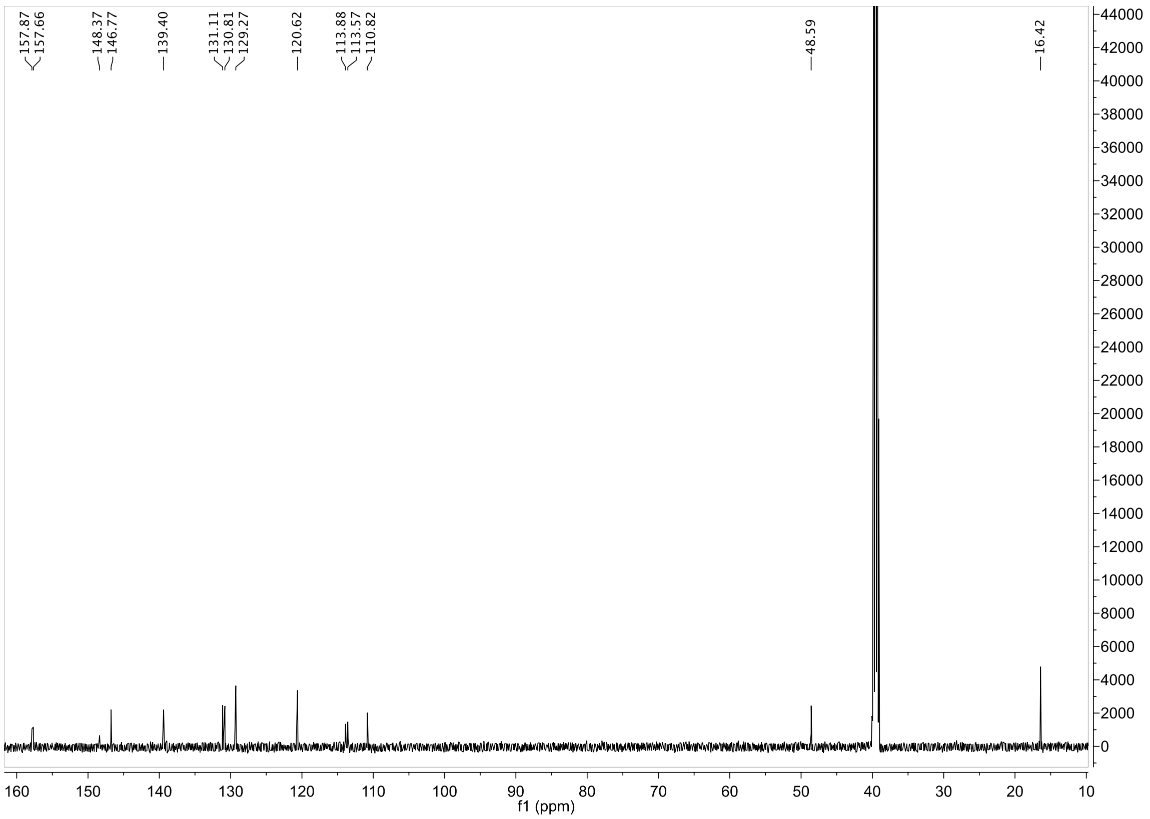

Figure S-24. ^{13}C NMR spectra for Tri-Click 3-ethynyl aniline (**TC4**).

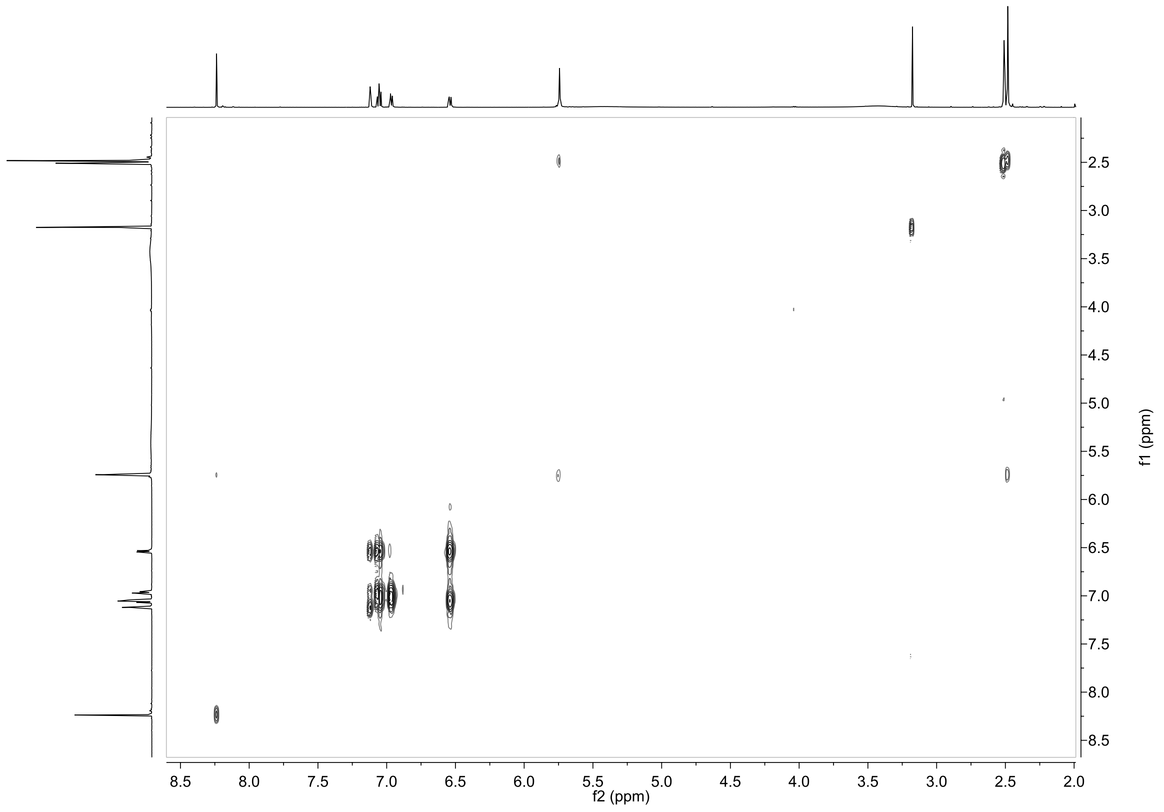

Figure S-25. Cosy NMR spectra for Tri-Click 3-ethynyl aniline (**TC4**).

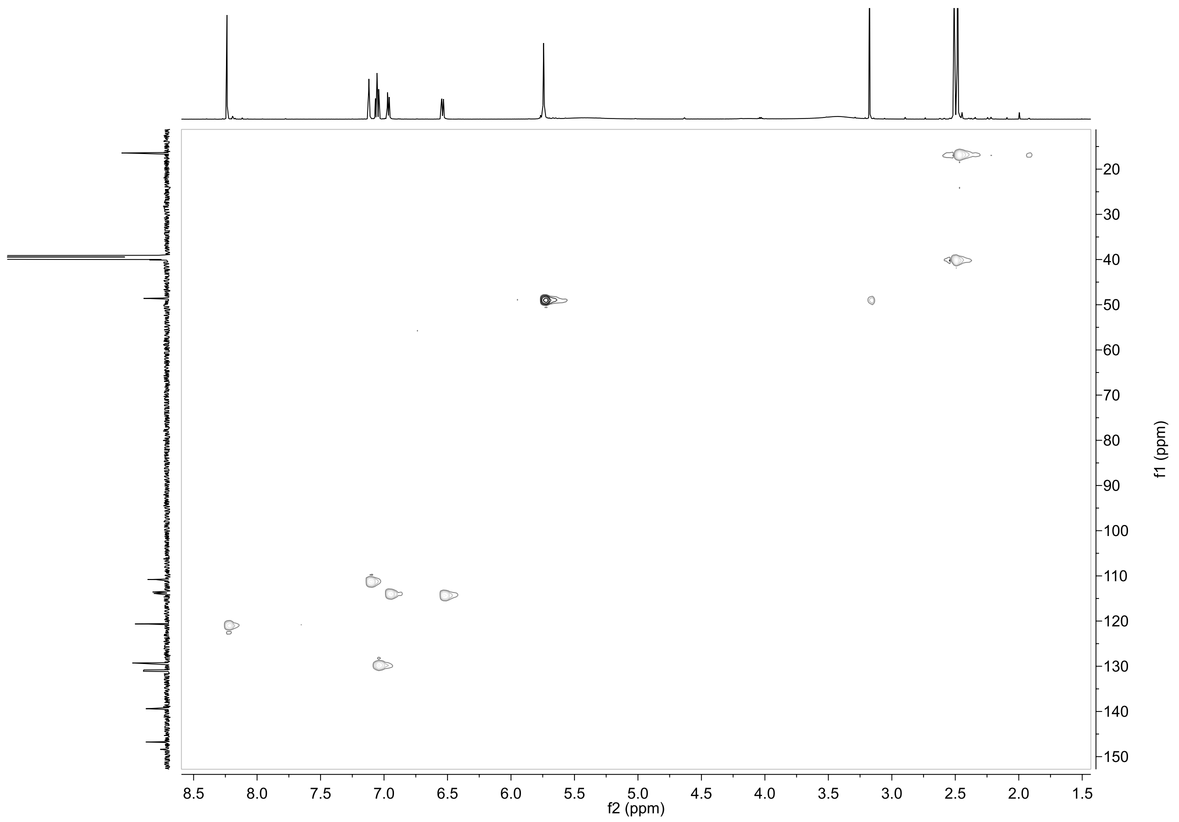

Figure S-26. HSQC NMR spectra for Tri-Click 3-ethynyl aniline (**TC4**).

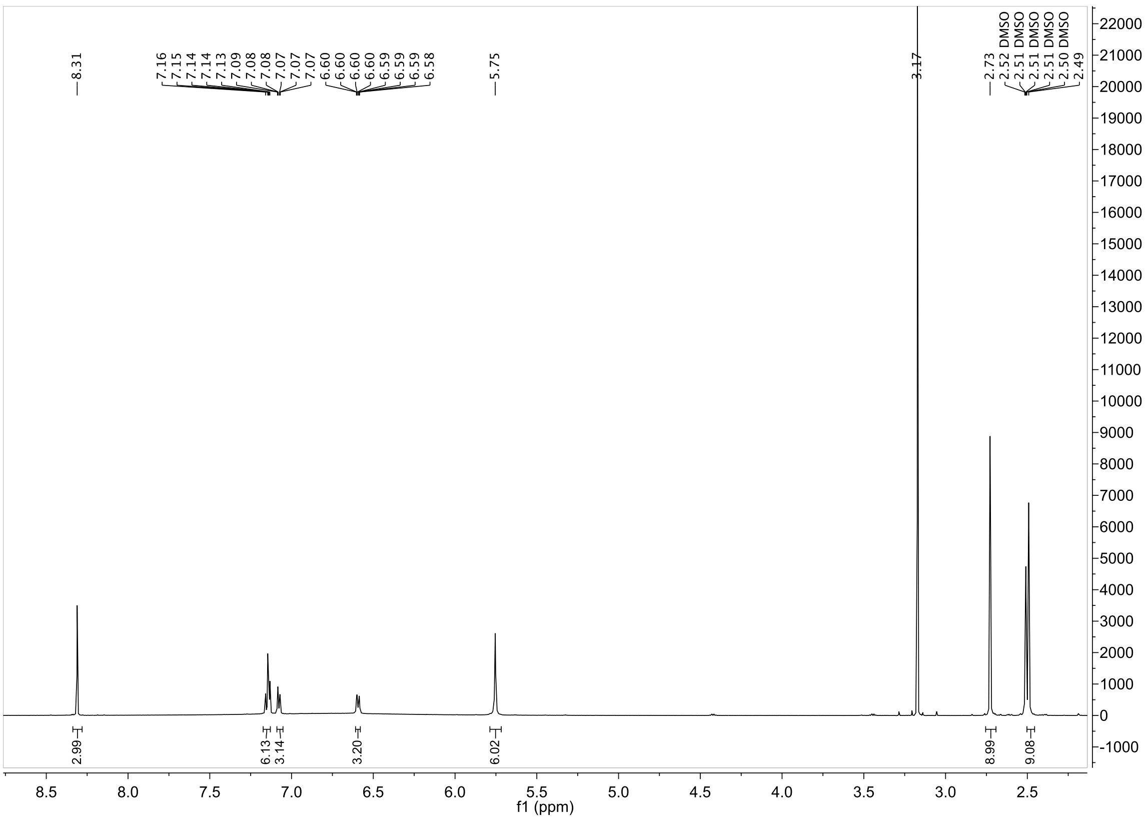

Figure S-27. ^{1}H NMR spectra of Tri-Click 3-ethynyl-methyl-aniline (**TC5**).

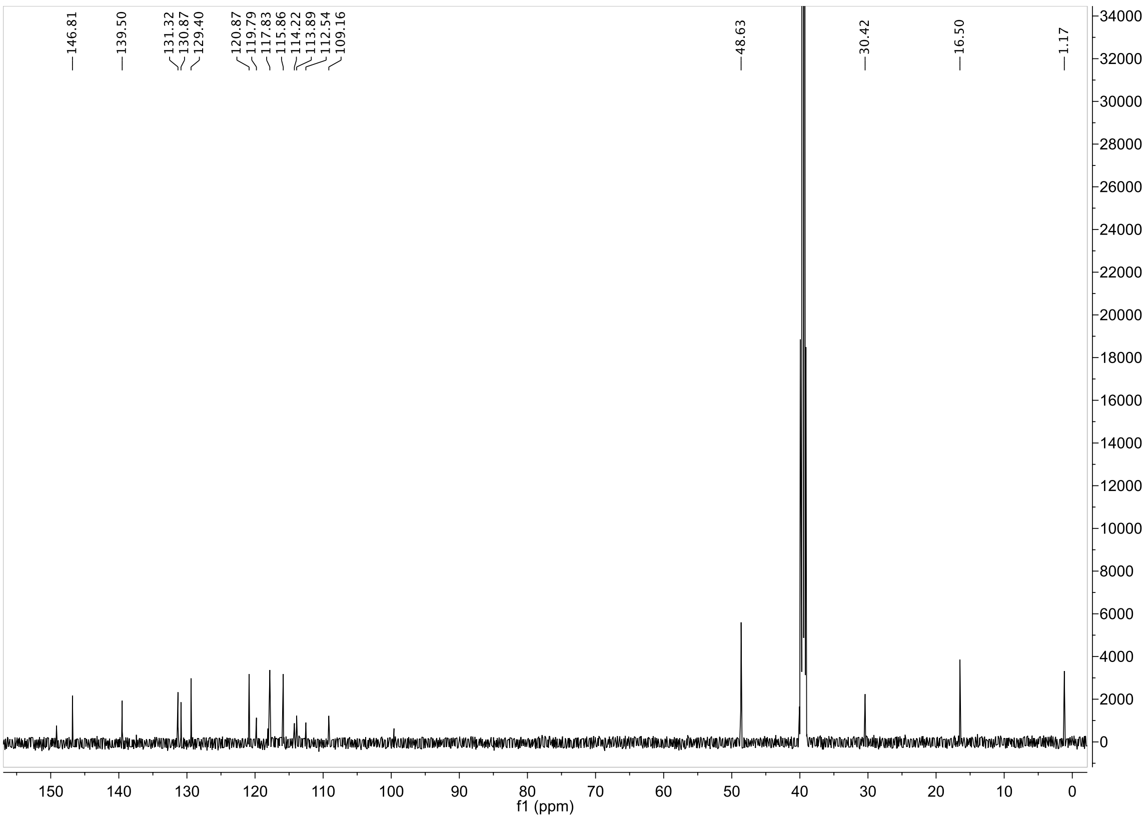

Figure S-28. ^{13}C NMR spectra of Tri-Click 3-ethynyl-methyl-aniline (**TC5**).

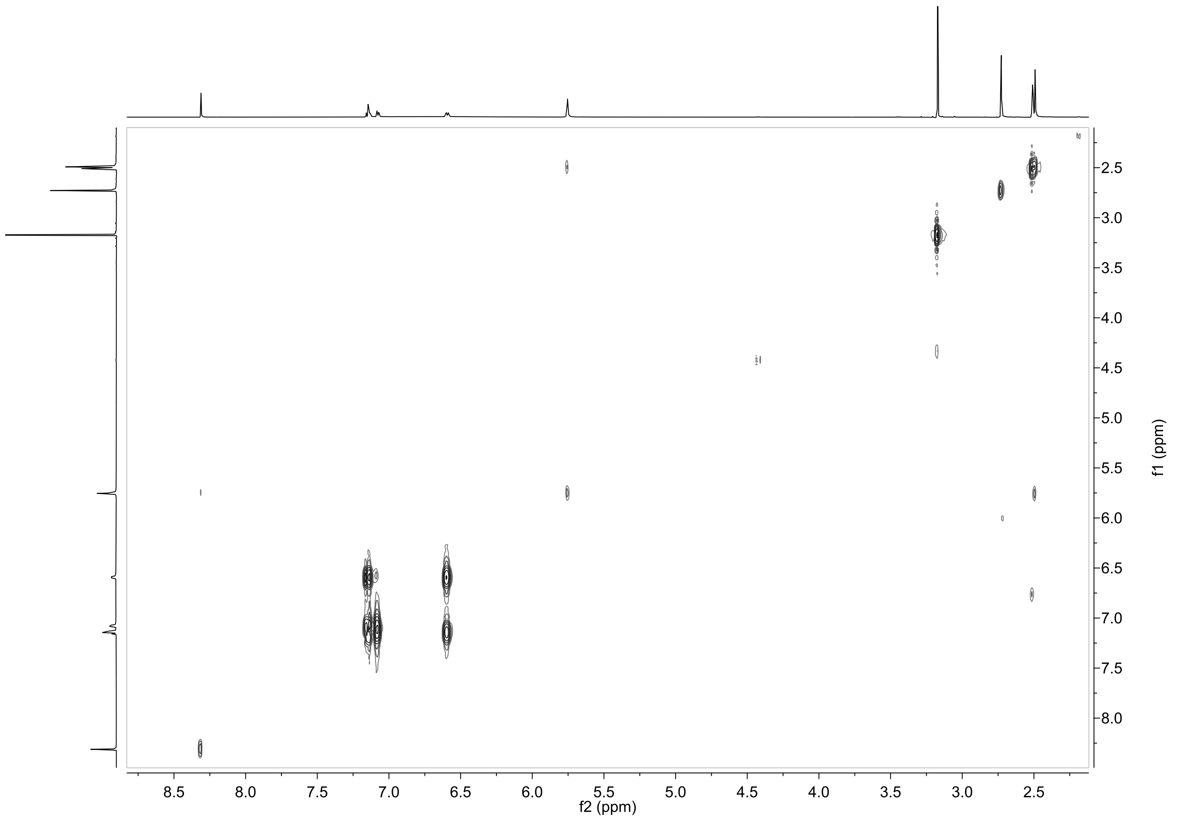

Figure S-29. COSY NMR spectra of Tri-Click 3-ethynyl-methyl-aniline (**TC5**).

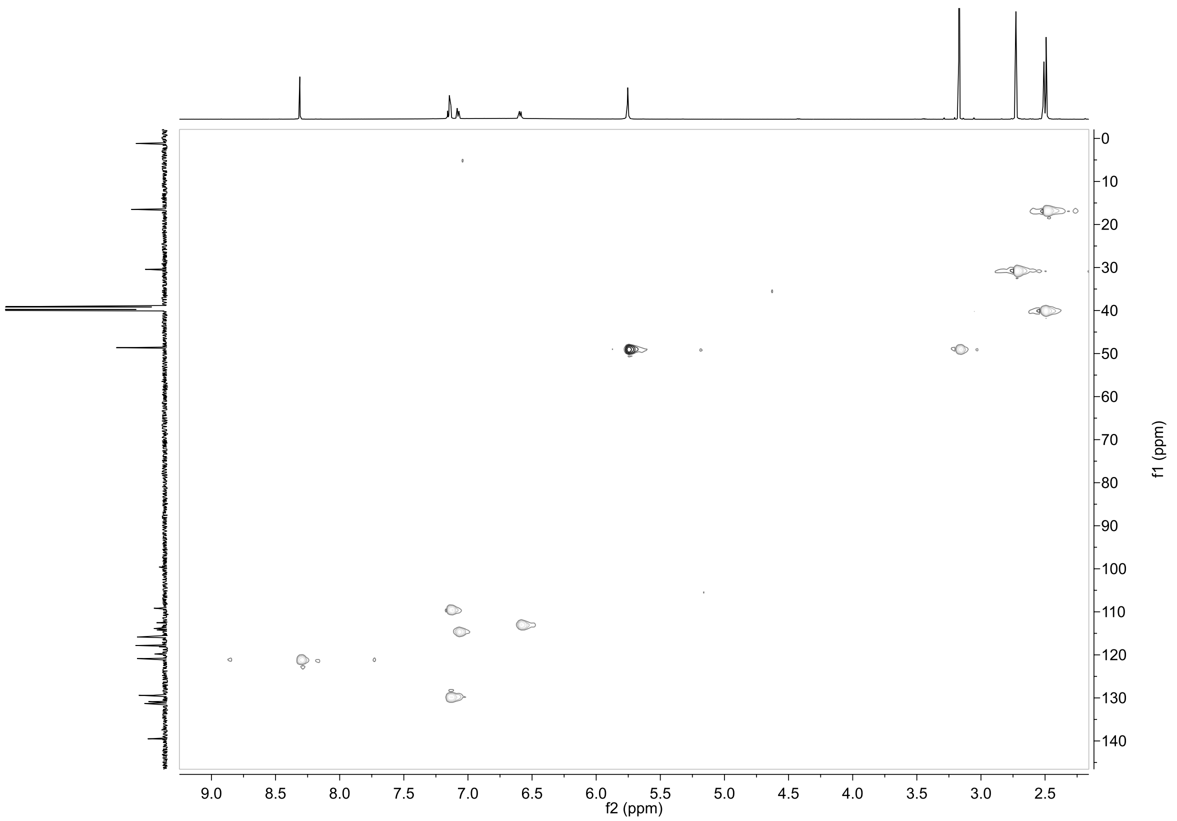

Figure S-30. HSQC NMR spectra of Tri-Click 3-ethynyl-methyl-aniline (**TC5**).

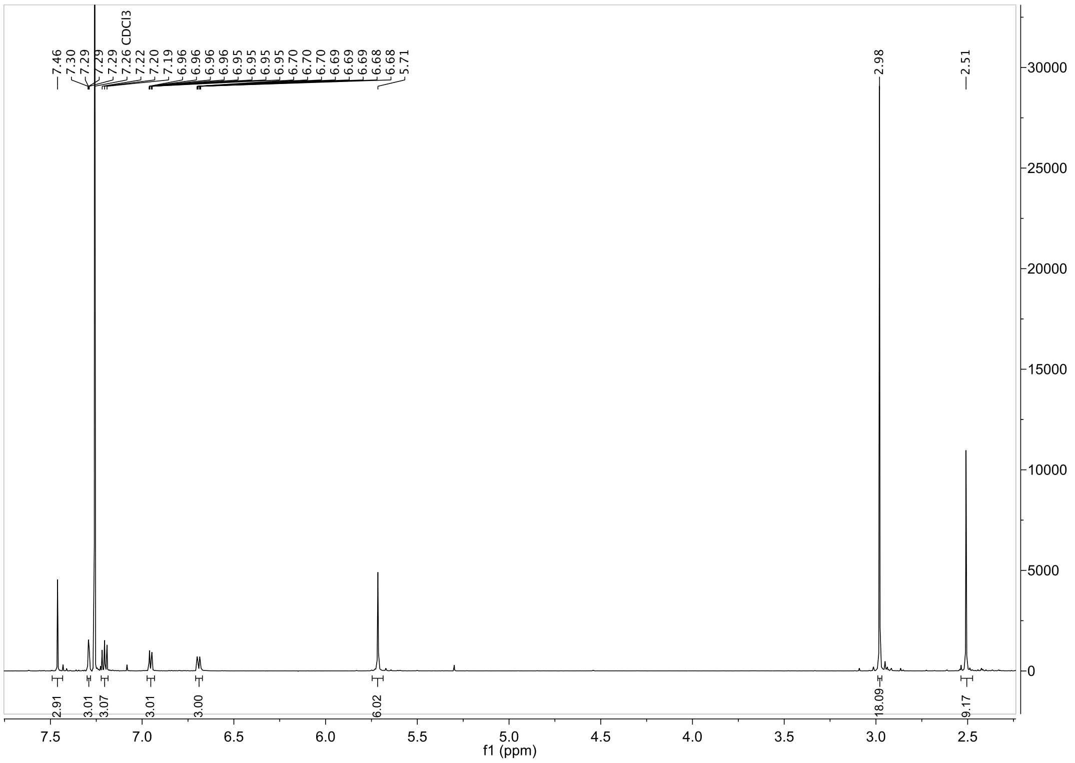

Figure S-31. ^{1}H NMR spectra for Tri-Click 3-ethynyl-*N,N*- dimethylaniline (**TC6**).

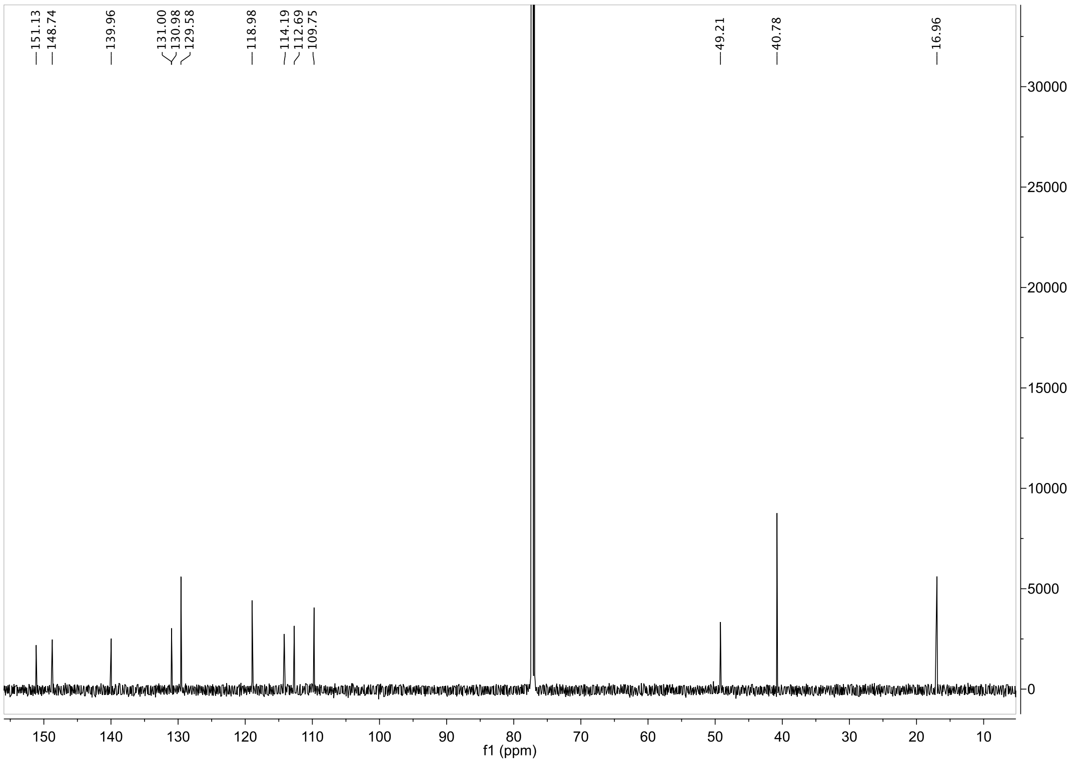

Figure S-32. ^{13}C NMR spectra for Tri-Click 3-ethynyl-*N,N*- dimethylaniline (**TC6**).

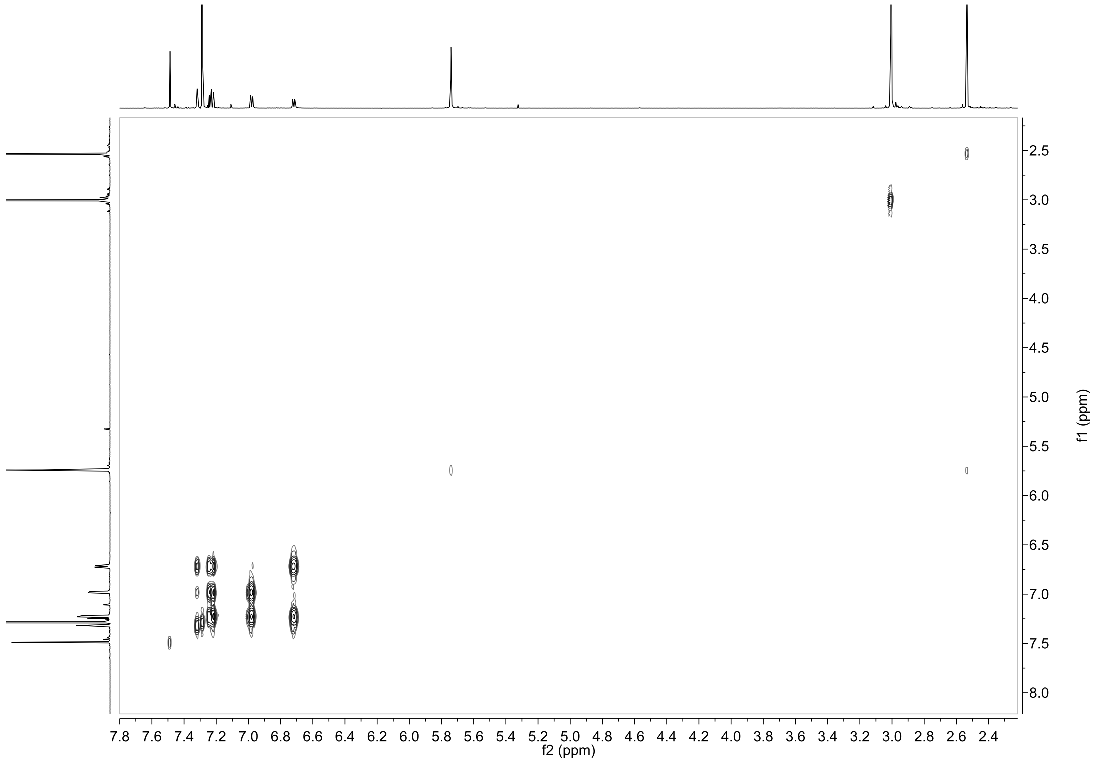

Figure S-33. COSY NMR spectra for Tri-Click 3-ethynyl-*N,N*- dimethylaniline (**TC6**).

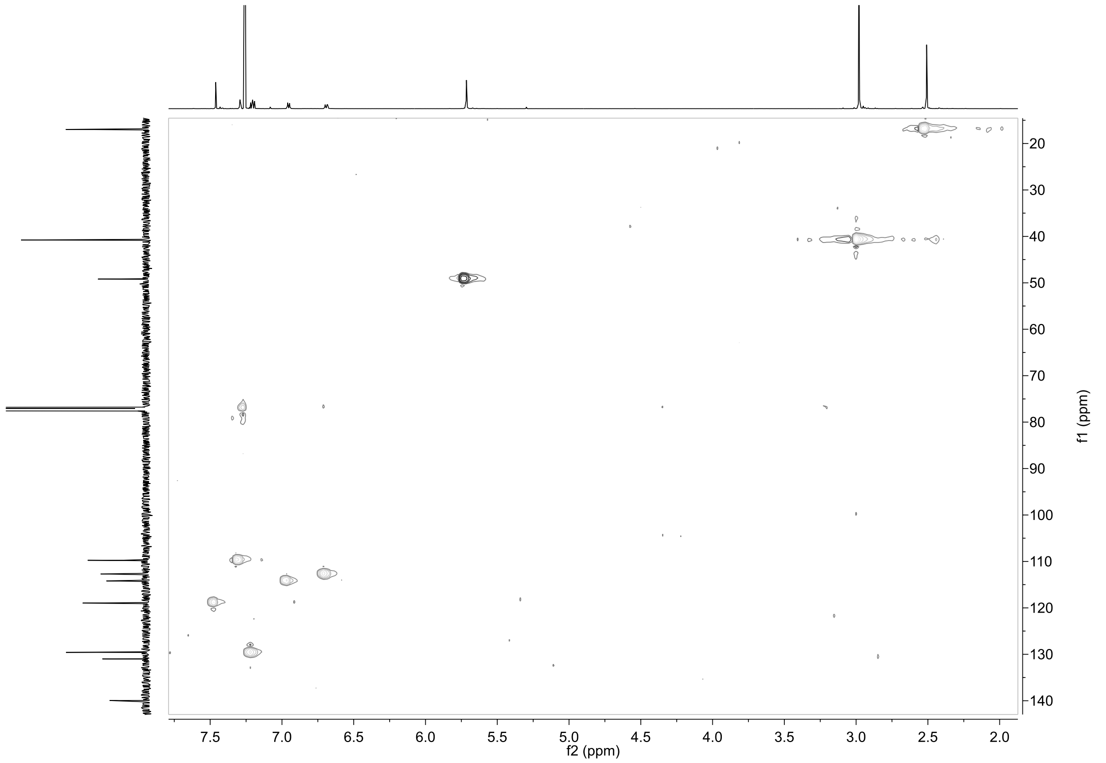

Figure S-34. HSQC NMR spectra for Tri-Click 3-ethynyl-*N,N*- dimethylaniline (**TC6**).

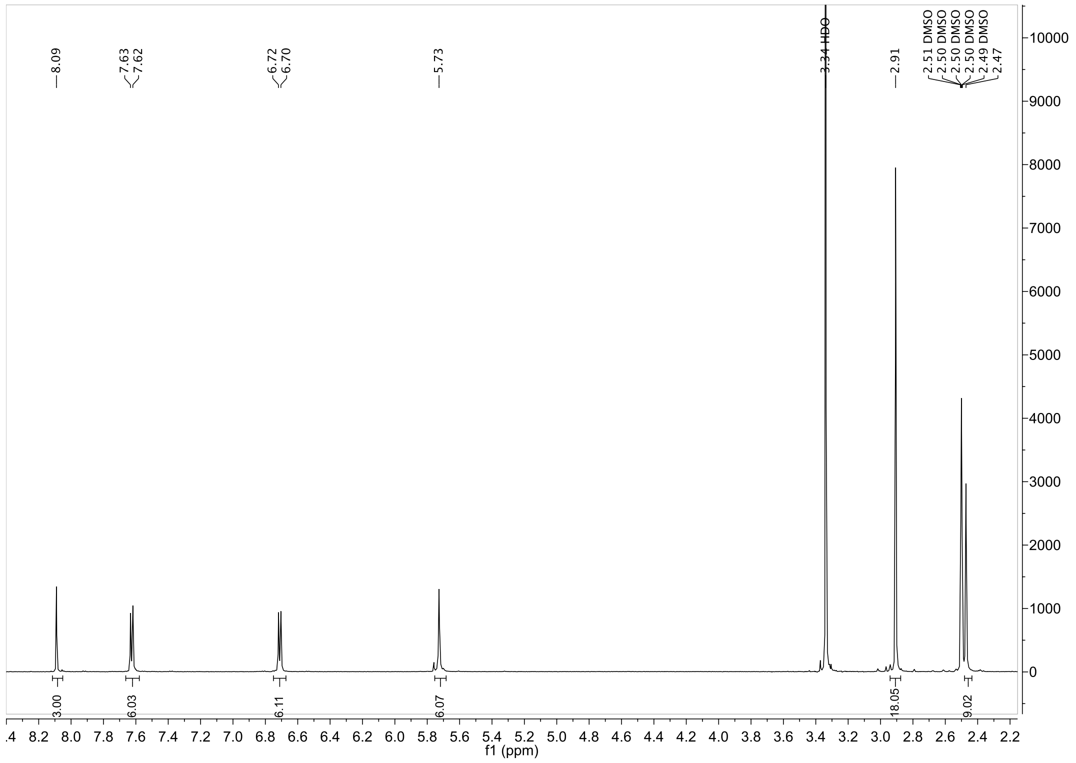

Figure S-35. ^{1}H NMR spectra for Tri-Click 4-ethynyl-*N*,*N*-dimethylaniline (**TC7**).

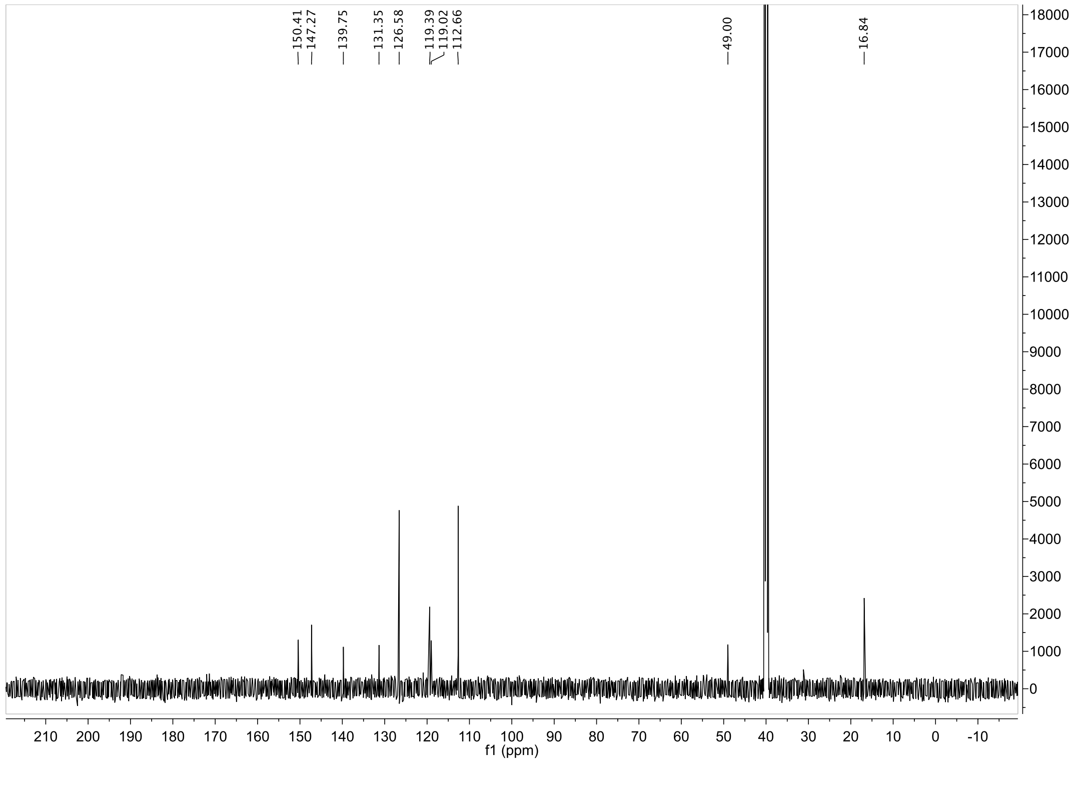

Figure S-36. ^{13}C NMR spectra for Tri-Click 4-ethynyl-*N*,*N*-dimethylaniline (**TC7**).

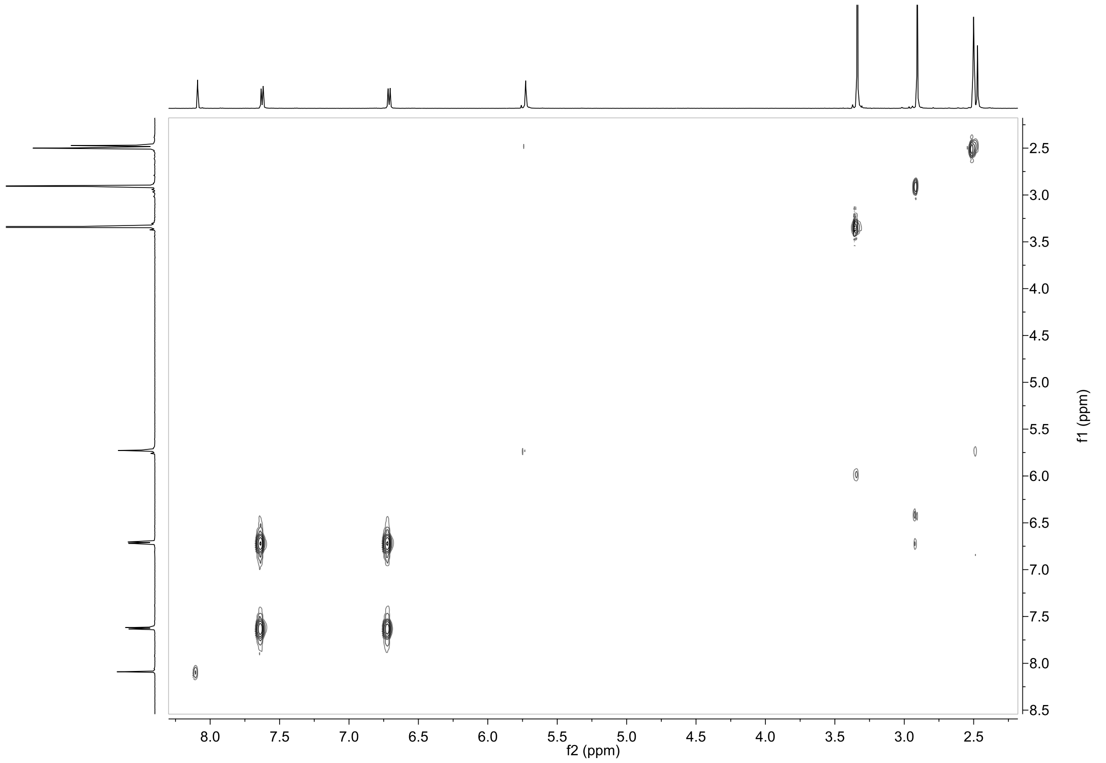

Figure S-37. COSY NMR spectra for Tri-Click 4-ethynyl-*N*,*N*-dimethylaniline (**TC7**).

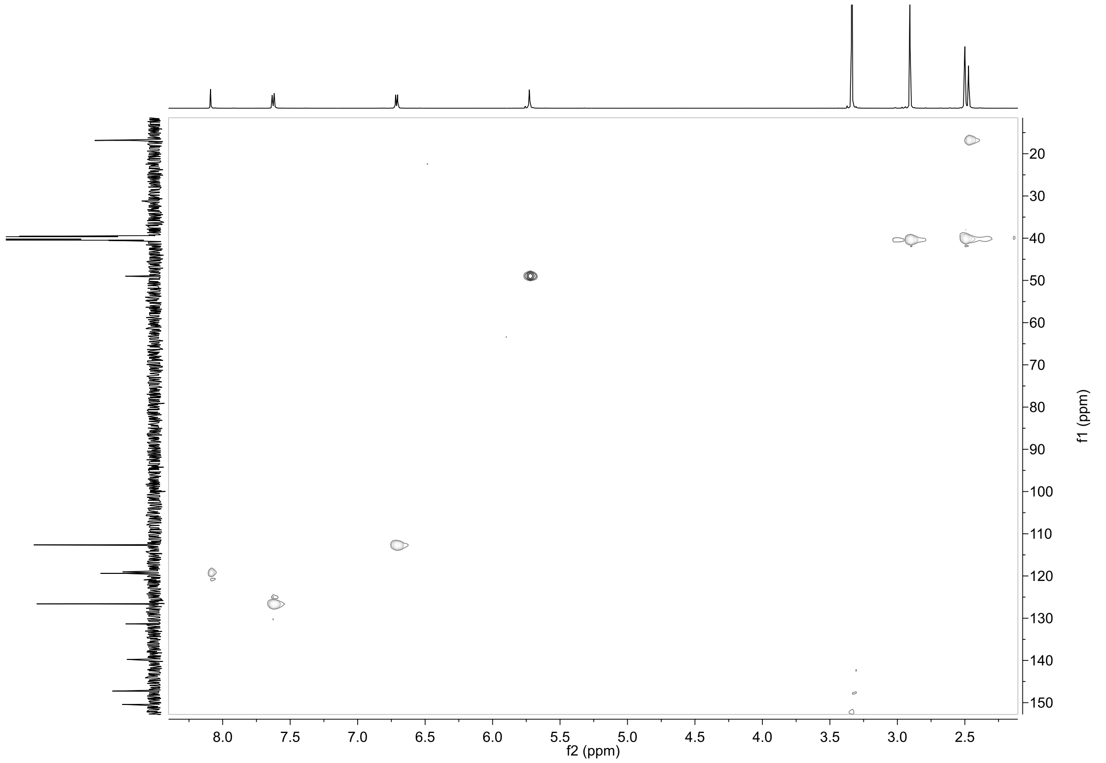

Figure S-38. HSQC NMR spectra for Tri-Click 4-ethynyl-*N*,*N*-dimethylaniline (**TC7**).

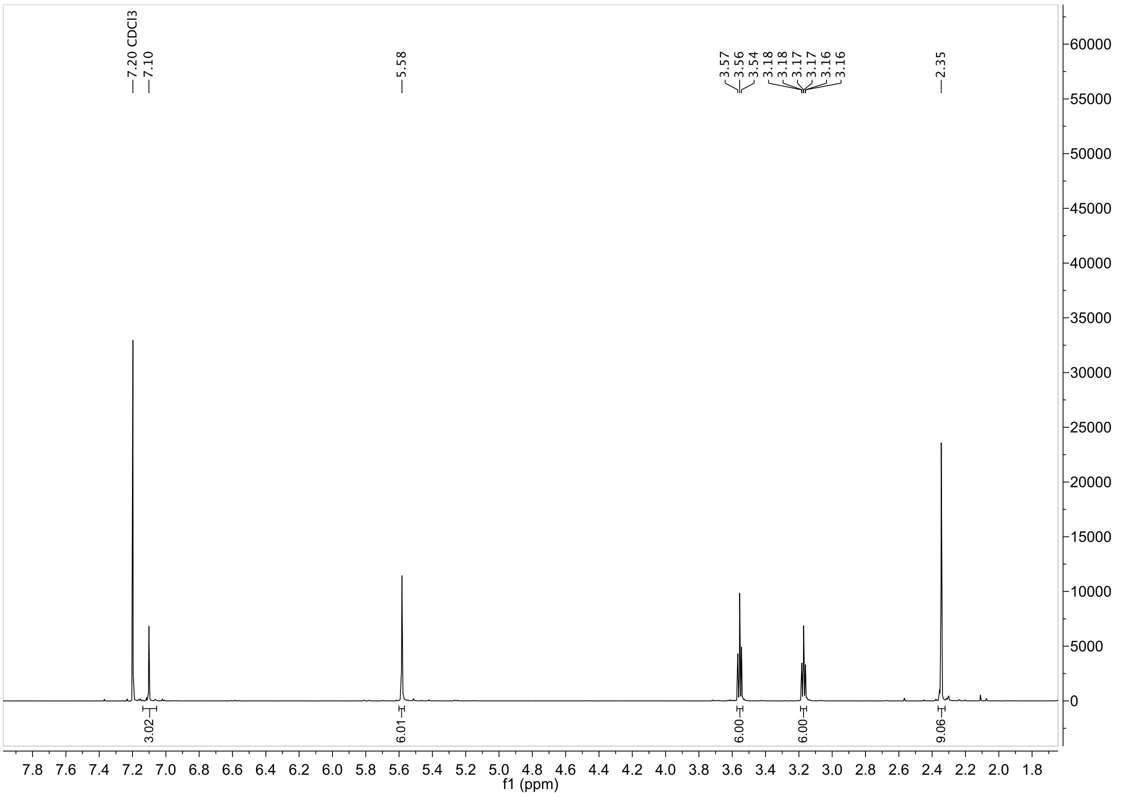

Figure S-39. ^{1}H NMR spectra for Tri-Click 4-bromo-1-butyne (**TC-Br**).

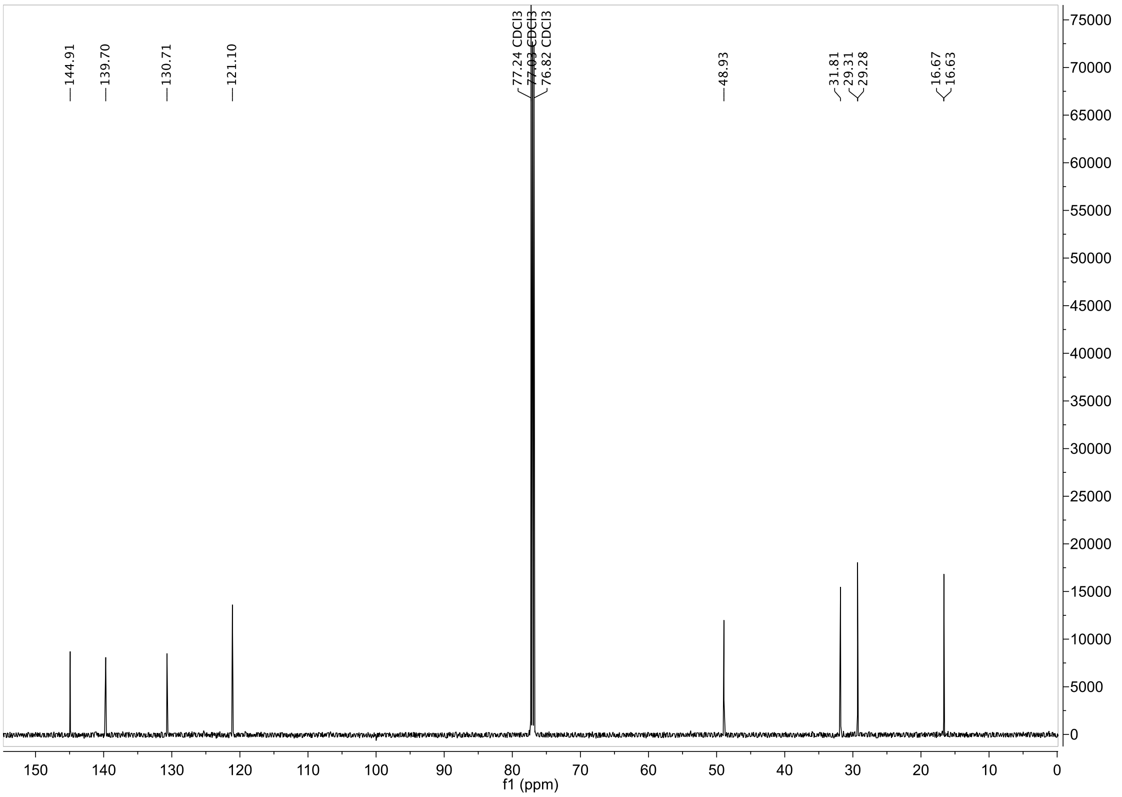

Figure S-40. ^{13}C NMR spectra for Tri-Click 4-bromo-1-butyne (**TC-Br**).

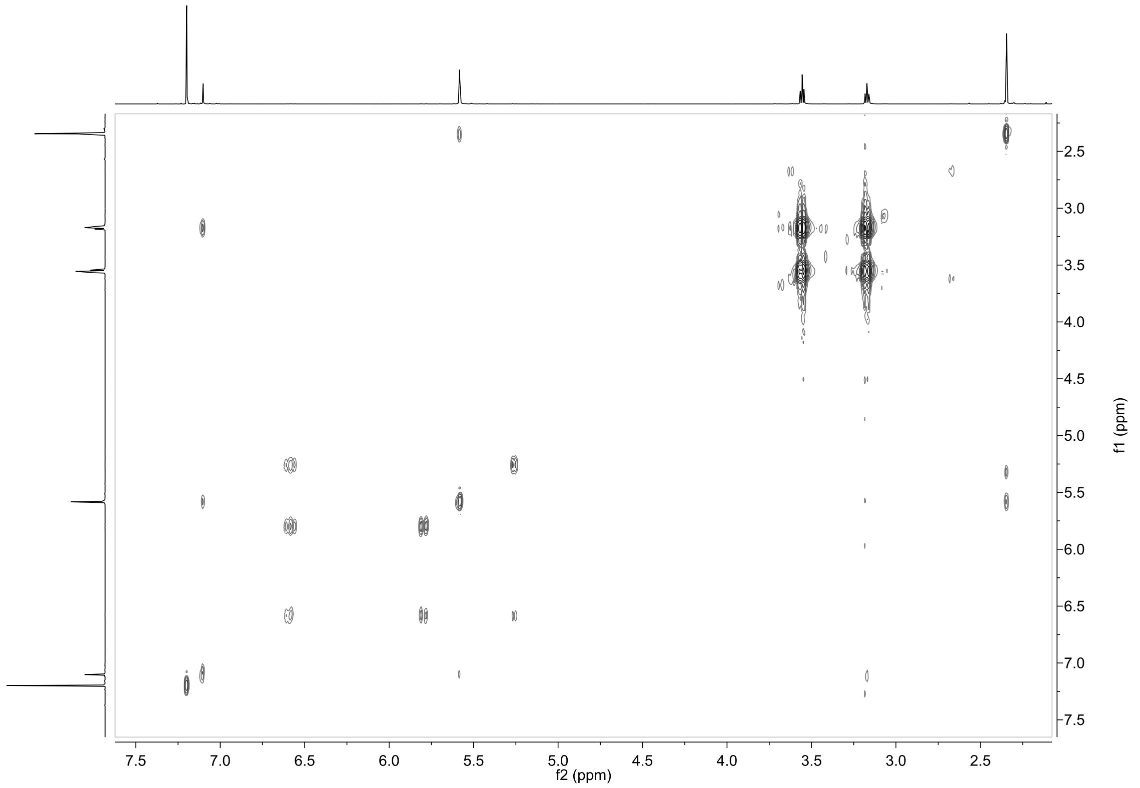

Figure S-41. COSY NMR spectra for Tri-Click-4-bromo-1-butyne (**TC-Br**).

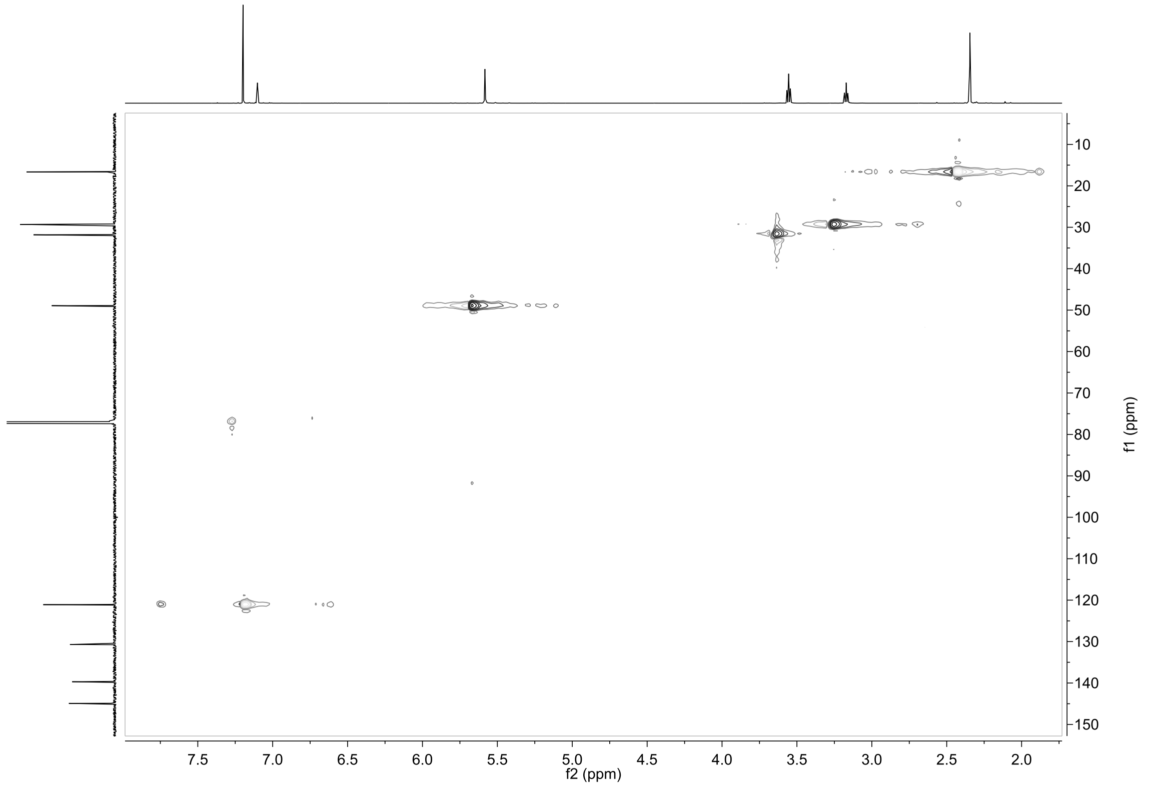

Figure S-42. HSQC NMR spectra for Tri-Click-4-bromo-1-butyne (**TC-Br**).

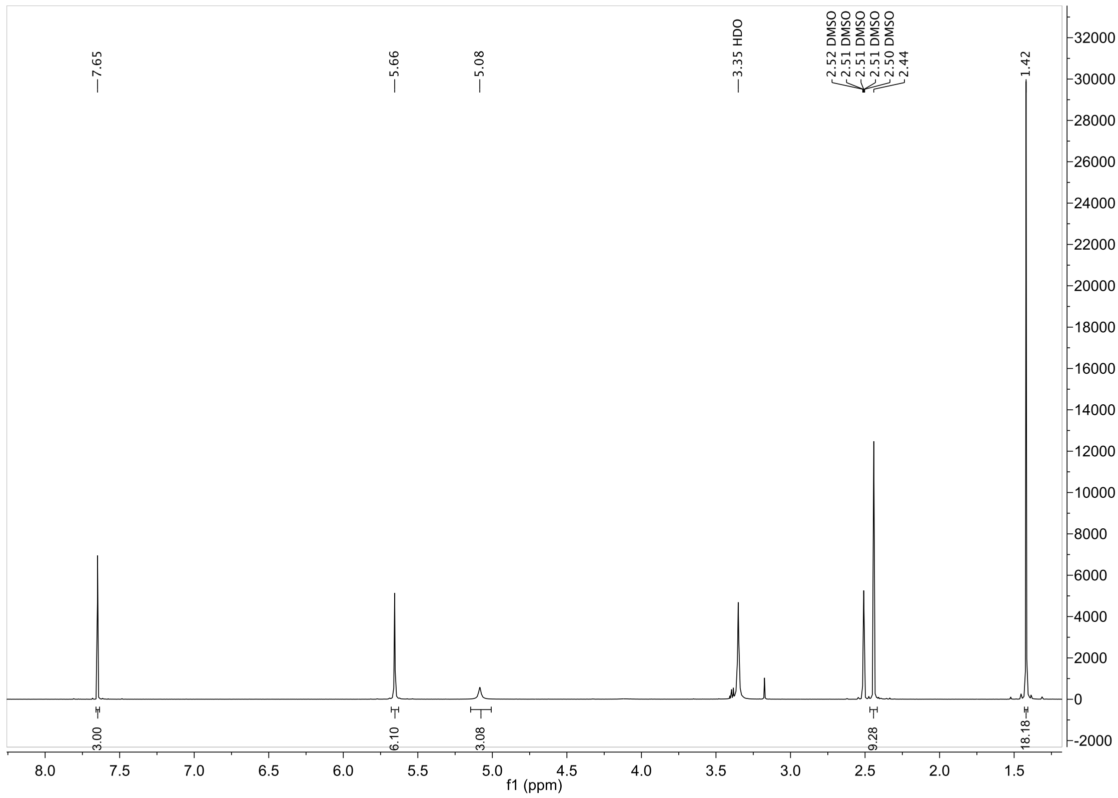

Figure S-43. ^{1}H NMR spectra for Tri-Click-2-methyl-3-butyne-2-ol (**TC-iPrOH**).

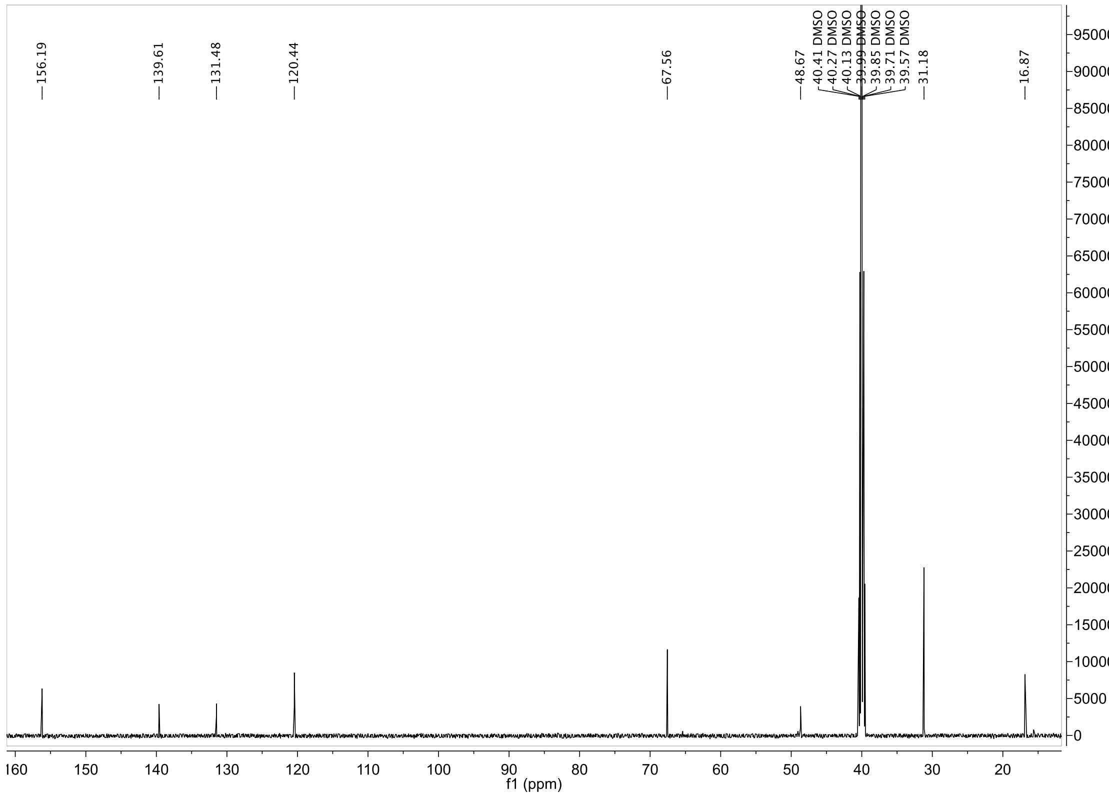

Figure S-44. ^{13}C NMR spectra for Tri-Click-2-methyl-3-butyne-2-ol (**TC-iPrOH**).

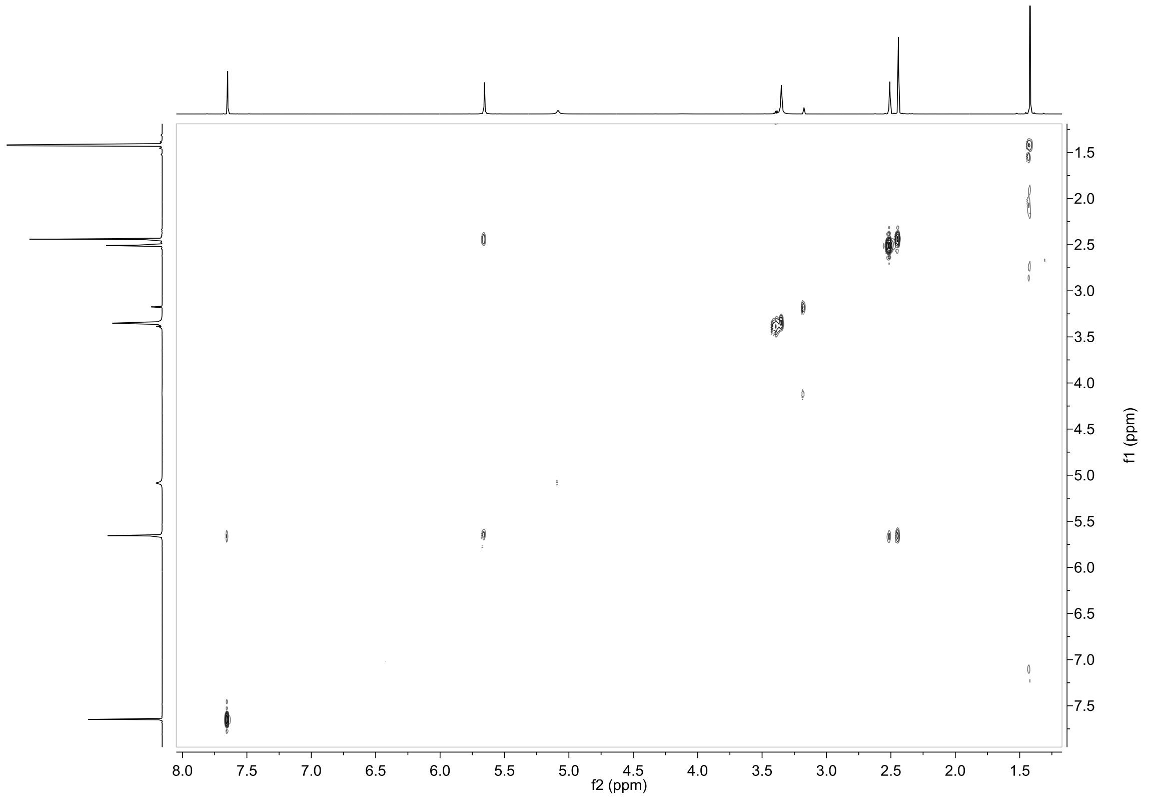

Figure S-45. COSY NMR spectra for Tri-Click 2-methyl-3-butyne-2-ol (**TC-iPrOH**).

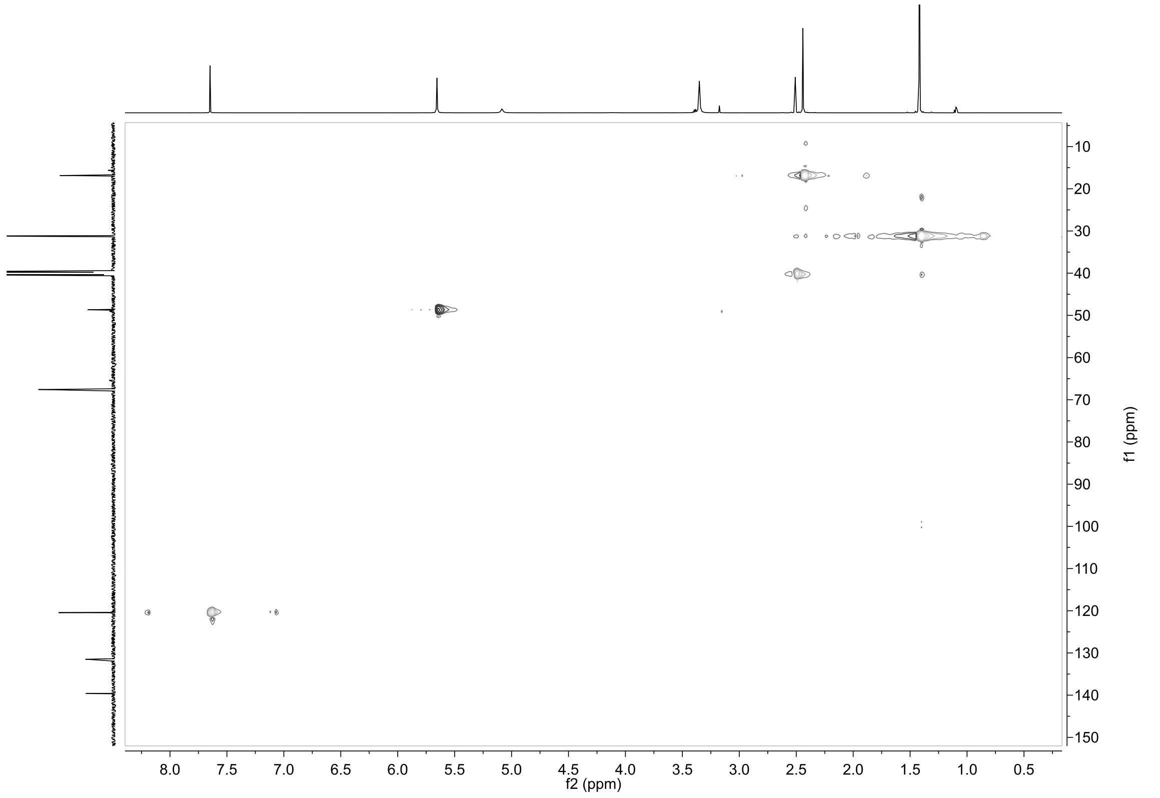

Figure S-46. HSQC NMR spectra for Tri-Click 2-methyl-3-butyne-2-ol (**TC-iPrOH**).
